# Antibody-lectin chimeras for glyco-immune checkpoint blockade

**DOI:** 10.1038/s41587-025-02884-6

**Published:** 2025-12-16

**Authors:** Jessica C. Stark, Melissa A. Gray, Itziar Ibarlucea-Benitez, Marta Lustig, Annalise Bond, Brian Cho, Ishika Govil, Tran Luu, Megan J. Priestley, Tim S. Veth, Wesley J. Errington, Bence Bruncsics, Mikaela K. Ribi, Leo A. Williams, Casim A. Sarkar, Simon Wisnovsky, Nicholas M. Riley, Meghan A. Morrissey, Thomas Valerius, Jeffrey V. Ravetch, Carolyn R. Bertozzi

**Affiliations:** 1 Department of Biological Engineering, MIT, Cambridge, MA USA; 2 Department of Chemical Engineering, MIT, Cambridge, MA USA; 3 Koch Institute for Integrative Cancer Research, MIT, Cambridge, MA USA; 4 Department of Chemistry and Sarafan ChEM-H, Stanford University, Stanford, CA USA; 5 Laboratory of Molecular Genetics and Immunology, The Rockefeller University, New York, NY USA; 6 Division of Stem Cell Transplantation and Immunotherapy, Department of Medicine II, Christian Albrechts University Kiel and University Medical Center Schleswig-Holstein, Kiel, Germany; 7 Department of Molecular Cellular and Developmental Biology, University of California, Santa Barbara, CA USA; 8 Department of Chemistry, University of Washington, Seattle, WA USA; 9 Department of Biomedical Engineering, University of Minnesota, Minneapolis, MN USA; 10 Department of Measurement and Information Systems, Budapest University of Technology and Economics, Budapest, Hungary; 11 Faculty of Pharmaceutical Sciences, University of British Columbia, Vancouver, British Columbia Canada; 12 Howard Hughes Medical Institute, Stanford, CA USA

**Keywords:** Molecular engineering, Applied immunology

## Abstract

Despite the curative potential of checkpoint blockade immunotherapy, many patients remain unresponsive to existing treatments. Glyco-immune checkpoints, which involve interactions of cell-surface glycans with lectin, or glycan-binding, immunoreceptors, have emerged as prominent mechanisms of immune evasion and therapeutic resistance in cancer. Here, we describe antibody-lectin chimeras (AbLecs), a modular system for glyco-immune checkpoint blockade. AbLecs are bispecific antibody-like molecules comprising a cell-targeting antibody domain and a lectin ‘decoy receptor’ domain that directly binds glycans and blocks their ability to engage inhibitory lectin receptors. AbLecs potentiate cancer cell destruction by primary human immune cells in vitro and reduce tumour burden in a humanized, immunocompetent mouse model, outperforming most existing therapies and combinations tested. By targeting a distinct axis of immunological regulation, AbLecs synergize with blockade of established immune checkpoints. AbLecs can be readily designed to target numerous tumours and immune cell subsets as well as glyco-immune checkpoints, thus representing a potential modality for cancer immunotherapy.

## Abstract (web summary)

Cancer immunotherapy benefits from antibody-lectin chimeras that block glyco-immune checkpoints.

## Main

Checkpoint blockade immunotherapies (for example, PD-1/PD-L1 or CTLA-4 antagonist antibodies) are now used to treat nearly half of all cancer patients in the United States^1^. However, these therapies are effective in only a small fraction (<20%) of treated patients^1^ and, of those who respond, at least 25% will develop resistance^2^. As a result, there is an urgent need for therapies targeting additional immune checkpoints that drive cancer progression.

Altered cell surface glycosylation is a hallmark of cancer that is associated with poor disease prognosis^3–5^. These altered glycan structures modulate immune cell function through interactions with lectin (glycan-binding) immunoreceptors, including sialic-acid-binding immunoglobulin-like lectins (Siglecs)^6^, selectins^7^ and galectins^8,9^. In particular, multiple recent studies indicate that upregulation of cell surface sialoglycans allows tumours to engage inhibitory Siglec receptors on immune cells and evade immune surveillance^10–34^. Taken together, glyco-immune checkpoints are emerging as attractive targets for cancer immunotherapy.

**Fig. 1:**
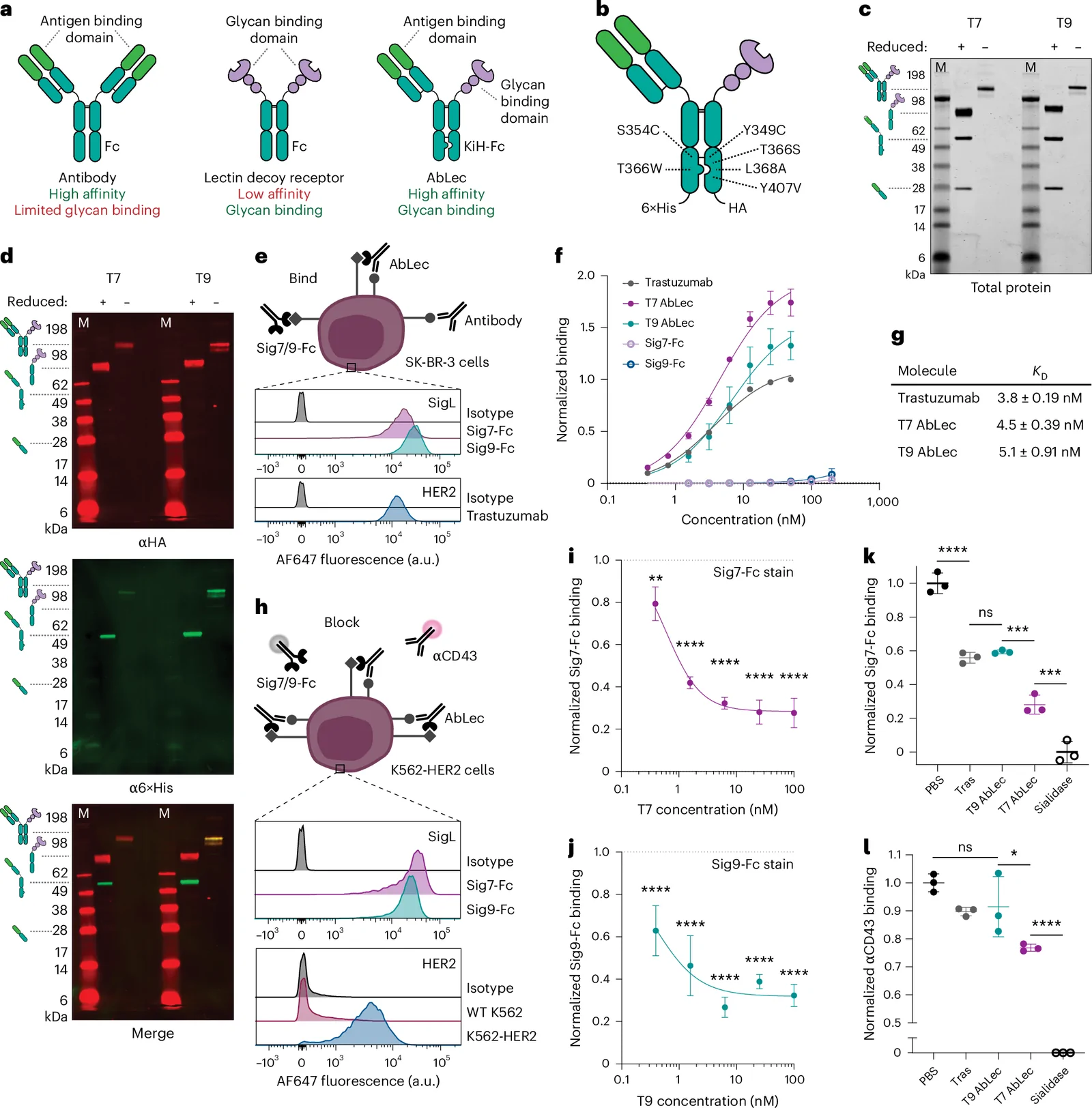
AbLecs enable targeted glyco-immune checkpoint blockade. **a**, AbLecs couple tumour-targeting antibodies to glycan-binding domains from lectin immunoreceptors, enabling blockade of immunosuppressive glycans at therapeutically relevant concentrations. **b**, Fc engineering in a human IgG1 antibody framework facilitates self-assembly of AbLecs. **c**, Reducing (+) and non-reducing (−) SDS–PAGE analysis of purified trastuzumab (αHER2) × Siglec-7 (T7) AbLec and trastuzumab × Siglec-9 (T9) AbLec. Gels are representative of *n* = 3 replicates. **d**, Western blot analysis of T7 and T9 AbLecs revealed that they are composed of trastuzumab light + heavy chains (α6×His) and Siglec-7/9-Fc chains (αHA), respectively. Blots are representative of *n* = 3 biological replicates. **e**, SK-BR-3 cells express the HER2 antigen bound by trastuzumab as well as ligands for Siglec-7 and Siglec-9 (Sig7L and Sig9L, respectively) as measured by flow cytometry. Histograms are representative of *n* = 3 biological replicates (quantified in Extended Data Fig. 1c,d). a.u., arbitrary units. **f**, Binding of T7 AbLec, T9 AbLec, trastuzumab, Siglec-7-Fc and Siglec-9-Fc to SK-BR-3 cells was quantified via flow cytometry. Data are means ± s.d. of *n* = 3 biological replicates, normalized to staining with 50 nM trastuzumab for each replicate. **g**, Apparent dissociation constants (*K*_D_) ±95% confidence intervals for binding of bivalent trastuzumab and T7/9 AbLecs were determined by fitting experimental data from **f** to one-site total binding curves. **h**, K562-HER2 cells express the HER2 antigen as well as Sig7L and Sig9L via flow cytometry. Histograms are representative of *n* = 3 biological replicates (quantified in Extended Data Fig. 1i,j). **i**,**j**, T7 (**i**) or T9 (**j**) AbLec treatment blocks Siglec-7 or Siglec-9 decoy receptor binding, respectively, in competitive binding assays with dye-labelled Siglec-7/9-Fc. Experimental data were fit using a four-parameter [inhibitor] vs response curve. Data are mean ± s.d. of *n* = 3 biological replicates, normalized to Siglec-Fc staining without AbLec. **k**, T7 AbLec, but not trastuzumab or T9 AbLec (25 nM each), blocks Siglec-7 decoy receptor binding in competitive binding assays with dye-labelled Siglec-7-Fc. Data are mean ± s.d. of *n* = 3 biological replicates, normalized to Siglec-7-Fc staining without AbLec or antibody. Sialidase-treated cells stained with Siglec-7-Fc are shown as a negative control. **l**, Detection of αCD43 antibody (MEM-59) binding in competitive binding assays with 25 nM T7 AbLec, T9 AbLec or trastuzumab. The MEM-59 antibody binds to the same epitope on CD43 recognized by the Siglec-7 receptor. Data are mean ± s.d. of *n* = 3 biological replicates, normalized to αCD43 staining without AbLec or antibody. Sialidase-treated cells stained with the MEM-59 antibody are shown as a negative control. *P* values were determined by Tukey-corrected one-way ANOVA in **i**–**l**; ns, not significant (*P* > 0.05); *P < 0.05; **P < 0.01; ***P < 0.001; ****P < 0.0001. Source data

However, multiple challenges have prevented therapeutic targeting of glyco-immune checkpoints in cancer (Fig. 1a). The weak immunogenicity of mammalian glycan structures can undermine the development of specific and high-affinity anti-glycan antibodies. To put this in perspective, a recent report catalogued 417 commercially available antibodies targeting glycans^35^. The same study noted that a single supplier offers 287 different antibodies to tubulin and 269 different antibodies to CD4, meaning that there are more antibodies to these two proteins available from a single supplier than all of the commercially available anti-glycan antibodies combined. Further complicating monoclonal antibody development is the fact that many lectin receptors have multiple ligands that are expressed in the context of cancer^10,15,17,23,36^, and the precise identities of their ligands are often unknown. ‘Decoy receptor’ molecules, comprising a lectin glycan-binding domain fused to an antibody Fc domain, address this complexity by retaining native glycan-binding specificities; however, their low binding affinities (typically with dissociation constants in the μM to mM range^37^) preclude their use as therapeutics.

We recently developed enzyme conjugates for targeted degradation of glycans^12,16^ or glycoproteins^38^ as an alternative approach to potentiate antitumor immunity; however, these molecules require substantial engineering of enzyme kinetics to target new types of glycans. In addition, such approaches are currently restricted to degradation of broad classes of glycans (for example, sialoglycans^12,16^) or glycoproteins (for example, mucins^38^) rather than the specific molecules involved in immune regulation. Finally, although lectin-targeted antagonist antibodies, such as those directed against galectin-9 (ref. ^39^) and Siglec-15 (ref. ^40^) as well as a sialidase-Fc fusion for sialoglycan degradation^41^, are being tested in the clinic, these untargeted checkpoint inhibitors could elicit immune-related adverse events, which are observed in at least 50% of patients treated with existing checkpoint blockade immunotherapies^42^. Indeed, ~42% of patients with cancer who were treated with a Siglec-15 blocking antibody experienced treatment-related adverse events in a recent clinical trial^43^, despite the more restricted expression profile of this receptor on immune cell subsets compared to other members of the Siglec family^6^. By contrast, targeted immunotherapies (for example, monoclonal antibodies targeting tumour-associated antigens) generally offer more favourable safety profiles^44^. Overall, our inability to specifically and directly block immunomodulatory glycans, even after the development of multiple therapeutic modalities, highlights the significance of this technological gap.

Here, we describe antibody-lectin chimeras (AbLecs) as a modular system to target glycans for cancer immunotherapy. AbLecs couple glycan-binding domains from lectin receptors to antibodies directed against an antigen on the target cell surface (Fig. 1a). This approach enables blockade of immunomodulatory glycans at nanomolar concentrations agnostic of their specific identities. We demonstrate proof-of-concept for the AbLec platform through the development and characterization of AbLecs combining tumour-targeting antibodies with decoy receptor domains from Siglec-7 and Siglec-9, which have been shown to mediate immune suppression in multiple cancers^11–13,16–25,28–30,33^. We show that tumour-targeting AbLecs enhance antibody-dependent phagocytosis and cytotoxicity of cancer cells in vitro and reduce tumour burden in vivo compared to the parent monoclonal antibody. Notably, we found that the chimeric AbLec architecture results in a gain-of-function arising from their ability to block Siglec engagement at the immunological synapse. As a result, AbLecs amplify inflammatory signalling, outperforming most existing therapies and combinations we tested in functional assays. We demonstrate that the AbLec platform can be applied to target several tumour-associated antigens, including HER2, CD20 and EGFR, and cancer cells with varying levels of antigen expression. Furthermore, we designed and validated dual blockade AbLecs that simultaneously block protein-based immune checkpoints (for example, PD-1/PD-L1, CD47/SIRPα) as well as glyco-immune checkpoints that restrain productive anticancer immune responses (for example, Siglec-7 and Siglec-9 (refs. ^11–13,16–25,28–30,33^), galectin-9 (refs. ^45,46^)). We found that glyco-immune checkpoint blockade was synergistic with blockade of these established immune checkpoints. These results indicate that AbLecs target a non-redundant axis of immune suppression in cancer and could expand the subset of patients who respond to immunotherapy. In sum, AbLecs represent a generalizable approach for glyco-immune checkpoint blockade and an alternative modality for cancer immunotherapy.

## Results

### AbLecs bind to targeted cells at nanomolar concentrations

We reasoned that high-affinity binding of an antibody Fab domain to cancer antigens would cause AbLecs to accumulate at a high local concentration on the cell surface. This high local concentration of the AbLec would then permit binding of a relatively low-affinity lectin domain to inhibitory glycans on the same cell. As proof-of-concept, we created AbLecs that combine the Fab domain from trastuzumab, an FDA-approved monoclonal antibody that binds the tumour-associated antigen HER2, with the extracellular domains of Siglec-7 or Siglec-9 fused to an Fc domain. Trastuzumab is used to treat HER2^+^ malignancies of diverse tissue origins, including breast, gastric and colon cancers. Trastuzumab exhibits a dual mechanism of action involving inhibition of oncogenic HER2 signalling as well as activation of innate and adaptive immune responses^47^. A select subset of patients treated with trastuzumab experience durable disease remission, indicating its potential to elicit lasting immunity to cancer^48,49^. However, acquired resistance to trastuzumab limits therapeutic efficacy in most patients^50,51^. Recent approval of margetuximab, a glycoengineered trastuzumab with improved ability to recruit and activate natural killer (NK) cells, shows that an enhanced immune response has the potential to overcome resistance to HER2-targeted therapies^52^. We suspected that trastuzumab-hybrid AbLecs could further amplify immune responses to HER2^+^ cancers through Siglec blockade^12,16,22^.

**Extended Data Fig. 1:**
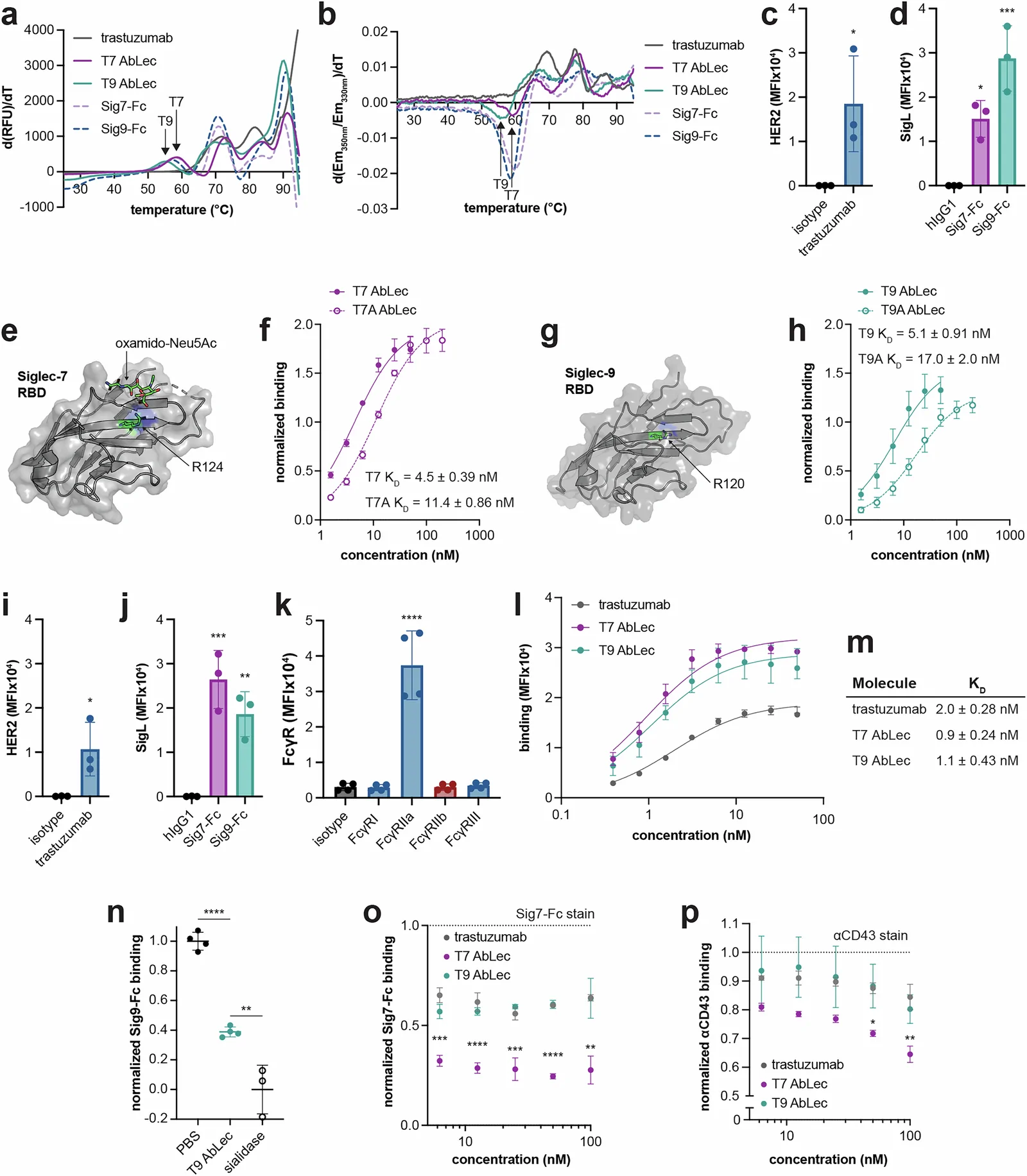
Characterization of AbLec stability, binding, and Siglec receptor blockade functionality. (**a, b**) Thermal stability of Siglec-7/9-Fc, trastuzumab, and T7/9 AbLecs was measured via the first derivative of SYPRO Orange fluorescence (**a**) or intrinsic tryptophan fluorescence (**b**) with respect to temperature. Both assays indicate melting temperatures for T7 and T9 AbLecs between 55–60 °C, similar to their corresponding Siglec-7/9-Fc arms and well above physiological temperature (37 °C). Traces represent averages of n = 3 biological replicates. (**c**) HER2 expression on SK-BR-3 cells measured via trastuzumab staining compared to hIgG1 isotype antibody by flow cytometry. Data are mean ± s.d. of n = 3 biological replicates. (**d**) Sig7L and Sig9L expression measured via Siglec-7/9-Fc staining of SK-BR-3 cells compared to hIgG1 isotype control. Data are mean ± s.d. of n = 3 biological replicates. (**e**) Crystal structure of Siglec-7 bound to the sialic acid mimetic, oxamido-Neu5Ac (PDB Code: 2G5R). The arginine at position 124 (R124), which mediates receptor binding to sialic acid-containing ligands, is highlighted on the protein backbone in green. (**f**) Binding of T7 AbLec or R124A AbLec mutant with reduced sialic acid binding (T7A AbLec) to SK-BR-3 cells was quantified via flow cytometry (n = 3, binding normalized to 50 nM trastuzumab for each experimental replicate). Apparent dissociation constants (K_D_) ± 95% confidence intervals for bivalent T7 and T7A AbLec binding to SK-BR-3 cells were determined by fitting experimental data to one-site total binding curves. Data are mean ± s.d. of n = 3 biological replicates. (**g**) Structure of Siglec-9 predicted using AlphaFold 3. The arginine at position 120 (R120), which mediates receptor binding to sialic acid-containing ligands, is highlighted on the protein backbone in green. (**h**) Binding of T9 AbLec or R120A AbLec mutant with reduced sialic acid binding (T9A AbLec) to SK-BR-3 cells was quantified via flow cytometry (n = 3, binding normalized to 50 nM trastuzumab for each experimental replicate). Apparent dissociation constants (K_D_) ± 95% confidence intervals for bivalent T9 and T9A AbLec binding to SK-BR-3 cells were determined by fitting experimental data to one-site total binding curves. Data are mean ± s.d. of n = 3 biological replicates. (**i**) HER2 expression on K562-HER2 cells measured via trastuzumab staining compared to hIgG1 isotype antibody by flow cytometry. Data are mean ± s.d. of n = 3 biological replicates. (**j**) Sig7L and Sig9L expression measured via Siglec-7/9-Fc staining of K562-HER2 cells compared to hIgG1 isotype control. Data are mean ± s.d. of n = 3 biological replicates. (**k**) Expression of human Fc gamma receptors FcγRI, FcγRIIa, FcγRIIb, and FcγRIII on K562-HER2 cells measured by flow cytometry. Data are mean ± s.d. of n = 4 biological replicates. (**l**) Binding of trastuzumab and T7/9 AbLecs to K562-HER2 cells was quantified via flow cytometry. Data are mean ± s.d. of n = 3 biological replicates. Experimental data were fit using one-site total binding curves. (**m**) Apparent dissociation constants (K_D_) ± 95% confidence intervals for bivalent trastuzumab and T7/9 AbLecs were determined by fitting experimental data from (**l**) to one-site total binding curves. (**n**) T9 AbLec (25 nM), blocks Siglec-9 receptor binding in competitive binding assays with dye labeled Siglec-9-Fc. Data are mean ± s.d. of n = 3 (sialidase) or n = 4 (PBS, T9 AbLec) biological replicates, normalized to Siglec-9-Fc staining without AbLec. Sialidase-treated cells stained with Siglec-9-Fc are shown as a negative control. (**o**) T7 AbLec, but not trastuzumab or T9 AbLec, blocks Siglec-7 receptor binding in competitive binding assays with dye labeled Siglec-7-Fc. Data are mean ± s.d. of n = 3 biological replicates, normalized to Siglec-7-Fc staining without AbLec or antibody. (**p**) Detection of αCD43 antibody (MEM-59) binding in competitive binding assays with increasing concentrations of T7 AbLec, T9 AbLec, or trastuzumab. The MEM-59 antibody binds to the same epitope on CD43 recognized by the Siglec-7 receptor. Data are mean ± s.d. of n = 3 biological replicates, normalized to αCD43 staining without AbLec or antibody. *P*-values were determined by two-tailed unpaired t test (**c**, **i**) or Tukey-corrected one-way ANOVA (**d**, **j**, **k**, **n**, **o**, **p**); ns p>0.05, *p<0.05, **p<0.01, ***p<0.001, and ****p<0.0001. Source data

We used a modified knobs-into-holes Fc engineering approach^53^ to facilitate self-assembly of heterotrimeric AbLecs in a single expression culture (Fig. 1b and Supplementary Table 1). Indeed, coexpression of trastuzumab heavy and light chains with Siglec-7-Fc or Siglec-9-Fc in Expi293 cells resulted in expression of predominant protein species with molecular weights consistent with heterotrimeric AbLecs (Fig. 1c). Reducing SDS–PAGE analysis of purified proteins showed that trastuzumab × Siglec-7 (T7) and trastuzumab × Siglec-9 (T9) AbLecs were composed of three disulfide bonded protein chains consistent with the molecular weights of the Siglec-Fc and the trastuzumab heavy and light chains (Fig. 1c). Western blotting against HA and 6×His tags on the Siglec-Fc and antibody heavy chains, respectively, demonstrated that full-length AbLecs are composed of both antibody and decoy receptor arms (Fig. 1d), which was confirmed by mass spectrometry (MS) (Supplementary Table 2). Thermal shift assays determined that T7 and T9 AbLec melting temperatures are between 55 °C and 60 °C, indicating stability at physiological temperatures (Extended Data Fig. 1a,b).

We next characterized binding of T7 and T9 AbLecs to SK-BR-3 cells compared to trastuzumab and Siglec decoy receptor controls. SK-BR-3 cells express the HER2 antigen bound by trastuzumab as well as ligands for Siglec-7 and Siglec-9 (Sig7L/Sig9L) (Fig. 1e and Extended Data Fig. 1c,d). As expected, the decoy receptor controls (Siglec-7/9-Fc) bind only at low levels to SK-BR-3 cells, even at the highest concentrations tested (200 nM), despite the fact that SK-BR-3 cells express Siglec-7 and Siglec-9 ligands (Fig. 1f). However, by combining the Siglec decoy receptor arm with the high-affinity trastuzumab antibody arm, AbLecs bind to SK-BR-3 cells with apparent dissociation constant (*K*_D_) values in the low nM range, similar to the parent bivalent antibody trastuzumab (Fig. 1f,g). Thus, AbLecs enable recruitment of otherwise low-affinity lectin binding domains to cell surfaces at nanomolar concentrations.

Despite the low affinity of the Siglec domain in comparison to the antibody arm, we wondered whether AbLec binding to cell surfaces was partially glycan-dependent. By mutating a conserved arginine residue in the Siglec-Fc binding site to an alanine, we created T7A and T9A AbLec mutants that are reported to exhibit significantly reduced affinities for Siglec ligands^54^. Arginine mutant AbLecs exhibited reduced binding to SK-BR-3 cells, measured by an approximately twofold to threefold increase in apparent *K*_D_ values compared to wild-type AbLecs (Extended Data Fig. 1e–h). These results indicate that Siglec–sialoglycan interactions contribute to AbLec binding, supporting the idea that AbLecs could occupy Siglec receptor binding sites on target cells.

### AbLecs selectively block lectin receptor binding epitopes

We next asked whether AbLecs could successfully block engagement of their targeted lectin receptors. To this end, we tested the ability of T7 and T9 AbLecs to compete with fluorescently labelled Siglec decoy receptors for binding to K562-HER2 cells (Fig. 1h). This cell line expresses a higher ratio of Sig7L and Sig9L to the targeted antigen HER2, offering a more challenging target for glycan blockade (Fig. 1h and Extended Data Fig. 1i,j). Notably, although K562-HER2 cells express the Fc gamma receptor FcγRIIa (Extended Data Fig. 1k), we conclude from comparison of Siglec-7/9-Fc and isotype control staining by flow cytometry that decoy receptor binding to the cell surface is primarily glycan-dependent (Fig. 1h and Extended Data Fig. 1j). Treatment of cells with T7 or T9 AbLec significantly reduced binding of fluorescently labelled Siglec-7-Fc or Siglec-9-Fc, respectively (Fig. 1i,j and Extended Data Fig. 1l–n). AbLecs further selectively block binding of the targeted lectin receptor. Treatment with the T7 AbLec blocked binding of the cognate Siglec-7 decoy receptor to a greater extent than trastuzumab or the non-cognate T9 AbLec at all concentrations tested (Fig. 1k and Extended Data Fig. 1o). Moreover, both T7 and T9 AbLecs reduced binding of their cognate Siglec decoy receptors almost to the level of a sialidase-treated control, in which target cells were pre-treated with a sialidase enzyme to degrade Siglec ligands before staining with fluorescently labelled Siglec-7/9-Fc (Fig. 1k and Extended Data Fig. 1n). In sum, we find that AbLecs can block most of the glycan-dependent binding of their cognate Siglec decoy receptor to target cells.

We wanted to test whether AbLecs block the same epitopes bound by their cognate lectin receptors. We recently reported that the predominant ligand for Siglec-7 expressed on K562 cells is the sialomucin CD43 (ref. ^17^). Furthermore, we found that the commercially available MEM-59 antibody binds to the same sialylated epitope on CD43 that is bound by Siglec-7 (ref. ^17^). We tested the ability of AbLecs to compete with the MEM-59 antibody for binding to K562-HER2 cells. We observed that the T7 AbLec blocked binding of MEM-59 to a greater extent than trastuzumab or the non-cognate T9 AbLec, suggesting that the T7 AbLec binds and blocks the same CD43 glycoform bound by the endogenous Siglec-7 immunoreceptor (Fig. 1l and Extended Data Fig. 1p). This evidence supports our hypothesis that AbLecs selectively block engagement of the targeted lectin receptors by competing for the same binding epitopes.

### Computational modelling provides insights into AbLec binding and Siglec receptor blockade

We further interrogated the mechanism of AbLec binding and Siglec receptor blockade by developing a structure-guided computational model of Siglec-7-Fc, trastuzumab and T7 AbLec binding trained using our experimental data (Supplementary Fig. 1a)^55,56^. This model allowed us to better understand the cooperative, bivalent binding of each reagent to multiple HER2 antigens and/or Siglec-7 ligands on K562-HER2 cells. Cooperativity in these bivalent interactions can be quantified by their avidities (*K*_D,eff_) and the effective concentration of the second arm relative to its target after binding of the first arm ([*C*_eff_]; Supplementary Fig. 1b). A higher [*C*_eff_] indicates a higher probability that an antibody or AbLec can engage in a cooperative, bivalent interaction at the cell surface. Our computational model estimated that the T7 AbLec [*C*_eff_] was more than two orders of magnitude higher than that of trastuzumab (Supplementary Fig. 1b; [*C*_eff, tras_] = 0.12 μM; [*C*_eff, T7_] = 25 μM), suggesting that cooperative binding of T7 AbLec to one HER2 and one Sig7L is much more likely than cooperative binding of trastuzumab to two HER2 antigens. The strong cooperativity of the trastuzumab and Siglec-7 decoy receptor arms helps explain why the apparent AbLec *K*_D_s are of the same order of magnitude as the apparent trastuzumab *K*_D_, despite having only one high-affinity antibody arm.

We next used the model-derived [*C*_eff_] values to compare the ability of T7 AbLec and Siglec-7-Fc to deliver Siglec-7 binding domains to the cell surface as a measure of their potency as Siglec-7 blockade reagents. By combining trastuzumab and Siglec-7 decoy receptor arms, the T7 AbLec is nearly 400 times more effective at delivering Siglec-7 binding domains than Siglec-7-Fc (Supplementary Fig. 1c), enabling the use of a decoy receptor approach for Siglec receptor blockade.

Finally, we used the modelled binding parameters to simulate the competitive binding experiments with T7 AbLec and Siglec-7-Fc (Supplementary Fig. 1d,e). Without any additional parameter fitting, our model closely approximates the experimentally determined inhibition curve (Fig. 1i and Supplementary Fig. 1f), building confidence in its description of T7 AbLec and Siglec-7-Fc binding. The model also provides deeper insights into why AbLecs are so effective in outcompeting Siglec decoy receptors for binding to K562-HER2 cells. T7 AbLec treatment shifts the Siglec-7-Fc binding mode from mostly bivalent to mostly monovalent (Supplementary Fig. 1g), the latter being a very weak interaction that readily dissociates. This finding is physiologically relevant, as clustering of Siglec receptors is thought to be required for effective inhibitory signalling^54,57,58^. Taken together, AbLecs decrease Siglec receptor binding by reducing their binding valency, and thus have the potential to disrupt inhibitory Siglec signalling.

### AbLecs enhance antibody effector functions by blocking glyco-immune checkpoints

**Fig. 2:**
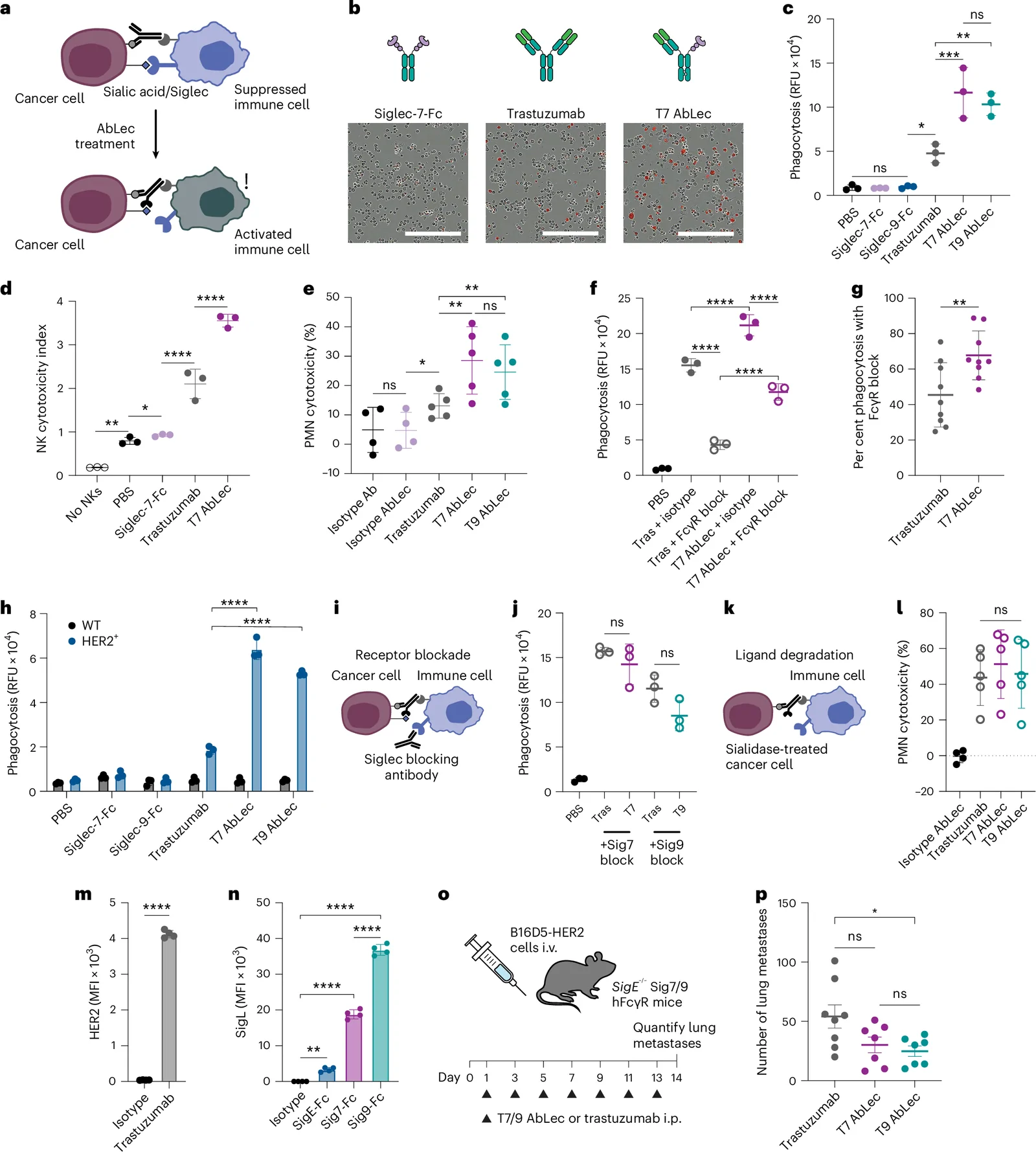
AbLecs enhance antibody effector functions in vitro and reduce tumour burden in vivo. **a**, Siglec engagement at immune synapses restrains anticancer immune responses. We reasoned that AbLecs would recruit FcγR^+^ immune cells to cancer cells and simultaneously block Siglec engagement, relieving inhibitory signalling and potentiating cancer cell killing. **b**, Phase contrast and fluorescence microscopy images of primary human macrophage/SK-BR-3 cell co-cultures treated with Siglec-7-Fc, trastuzumab or T7 AbLec at *t* = 5 h. SK-BR-3 cells were stained with the pHrodo red pH-sensitive dye, which fluoresces upon SK-BR-3 phagocytosis by macrophages. Images are representative of *n* = 3 biological replicates. Scale bar, 400 μm. **c**, Phagocytosis of SK-BR-3 cells by primary human macrophages elicited by T7/9 AbLecs, trastuzumab or Siglec-7/9-Fc. Data are mean ± s.d. of *n* = 3 biological replicates. RFU, relative fluorescence units. **d**, Cytotoxicity of SK-BR-3 cells elicited by primary human NK cells treated with T7 AbLec, trastuzumab or Siglec-7-Fc. Data are mean ± s.d. of *n* = 3 biological replicates. **e**, Cytotoxicity of SK-BR-3 cells elicited by primary human granulocytes (PMNs) treated with T7/9 AbLecs, trastuzumab or Siglec-7/9-Fc. Data are mean ± s.d. of biological replicates from *n* = 4 (isotype Ab/AbLec) or *n* = 5 (trastuzumab, T7/9 AbLec) donors. **f**, Primary human macrophages were pre-treated with FcγR blocking or isotype control antibodies before co-culture with SK-BR-3 cells and addition of trastuzumab (tras) or T7 AbLec. Data are mean ± s.d. of *n* = 3 biological replicates. **g**, Per cent phagocytosis observed upon FcγR blockade (+FcγR block RFU/+isotype RFU). Data are mean ± s.d. of *n* = 3 biological replicates from each of *n* = 3 donors. **h**, Phagocytosis of K562-HER2 cells compared to wild-type (WT) K562 cells, which are HER2^−^ (Fig. 1h and Extended Data Fig. 3c), elicited by T7/9 AbLecs, trastuzumab or Siglec-7/9-Fc. Data are mean ± s.d. of *n* = 3 biological replicates. **i**, Siglec-7 and Siglec-9 blocking antibodies were used to assess whether AbLec-mediated immune enhancement was dependent on the targeted Siglec receptor. **j**, Macrophage ADCP of SK-BR-3 cells elicited by trastuzumab or T7/9 AbLecs in the presence of Siglec-7 or Siglec-9 blocking antibodies (αSig7 or αSig9, respectively). Primary human macrophages were pre-treated with αSig7 or αSig9 before co-culture with SK-BR-3 cells. Data are mean ± s.d. of *n* = 3 biological replicates. **k**, Sialidase was used to degrade Siglec ligands on SK-BR-3 cells before use in immune cell assays to assess whether AbLec-mediated immune enhancement was glycan-dependent. **l**, Granulocyte (PMN) ADCC of SK-BR-3 cells pre-treated with sialidase to degrade Siglec ligands elicited by trastuzumab or T7/9 AbLecs. Data are mean ± s.d. of biological replicates from *n* = 4 (isotype AbLec) or *n* = 5 (trastuzumab, T7/9 AbLecs) donors. **m**,**n**, Expression of HER2 (**m**) and ligands for Siglec-E, Siglec-7 and Siglec-9 (**n**) on B16D5-HER2 cells measured by flow cytometry. Data are mean ± s.d. from *n* = 4 biological replicates. **o**, We established a humanized mouse model of metastasis to assess AbLec efficacy in vivo. This model uses B16D5-HER2 cancer cells injected intravenously (i.v.), which form metastatic nodules in the lungs. The lung colonization experiment is run using transgenic *SigE*^−/−^ Sig7/9 hFcγR mice that we developed, which are humanized for both Siglec-7 and Siglec-9 (ref. ^20^) and human FcγR expression^63^, with the corresponding murine ortholog receptors (Siglec-E, murine FcγRs) knocked out. Mice were dosed intraperitoneally (i.p.; black arrows) with trastuzumab or T7/9 AbLecs every other day following B16D5-HER2 inoculation, and lungs were dissected and analysed for surface metastatic foci on day 14 after inoculation. **p**, Quantification of lung metastases in mice inoculated with B16D5-HER2 cells i.v. and treated with trastuzumab or T7/9 AbLecs. Data are mean ± s.d. from *n* = 7 (T7/9 AbLec) or *n* = 8 (trastuzumab) biological replicates. *P* values were determined by Tukey-corrected one-way ANOVA (**c**, **d**, **f**, **h**, **j**, **n** and **p**), Hierarch’s many-sample, multiple hypothesis test with Benjamini–Hochberg multiple comparisons correction (**e** and **l**) or two-tailed unpaired *t*-test (**g** and **m**); ns, not significant (*P* > 0.05); *P < 0.05; **P < 0.01; ***P < 0.001; ****P < 0.0001. Source data

Having demonstrated that AbLecs block the binding of Siglec receptors to target cells, we investigated whether AbLec treatment could elicit immune responses against cancer cells. Therapeutic tumour-targeting antibodies create immune synapses between antigens on the cancer cell surface and Fc gamma receptors (FcγRs) on immune cells. Engagement of FcγRs activates the immune cell to perform antibody effector functions, including antibody-dependent cellular phagocytosis (ADCP) and antibody-dependent cellular cytotoxicity (ADCC), which result in the destruction of the targeted cancer cell. However, interactions between Siglec receptors on immune cells and Siglec ligands on the cancer cell surface negatively regulate antibody effector functions^12,16,59^. By contrast, we reasoned that AbLecs would block Siglec engagement at the immune synapse and enhance antibody effector functions (Fig. 2a).

**Extended Data Fig. 2:**
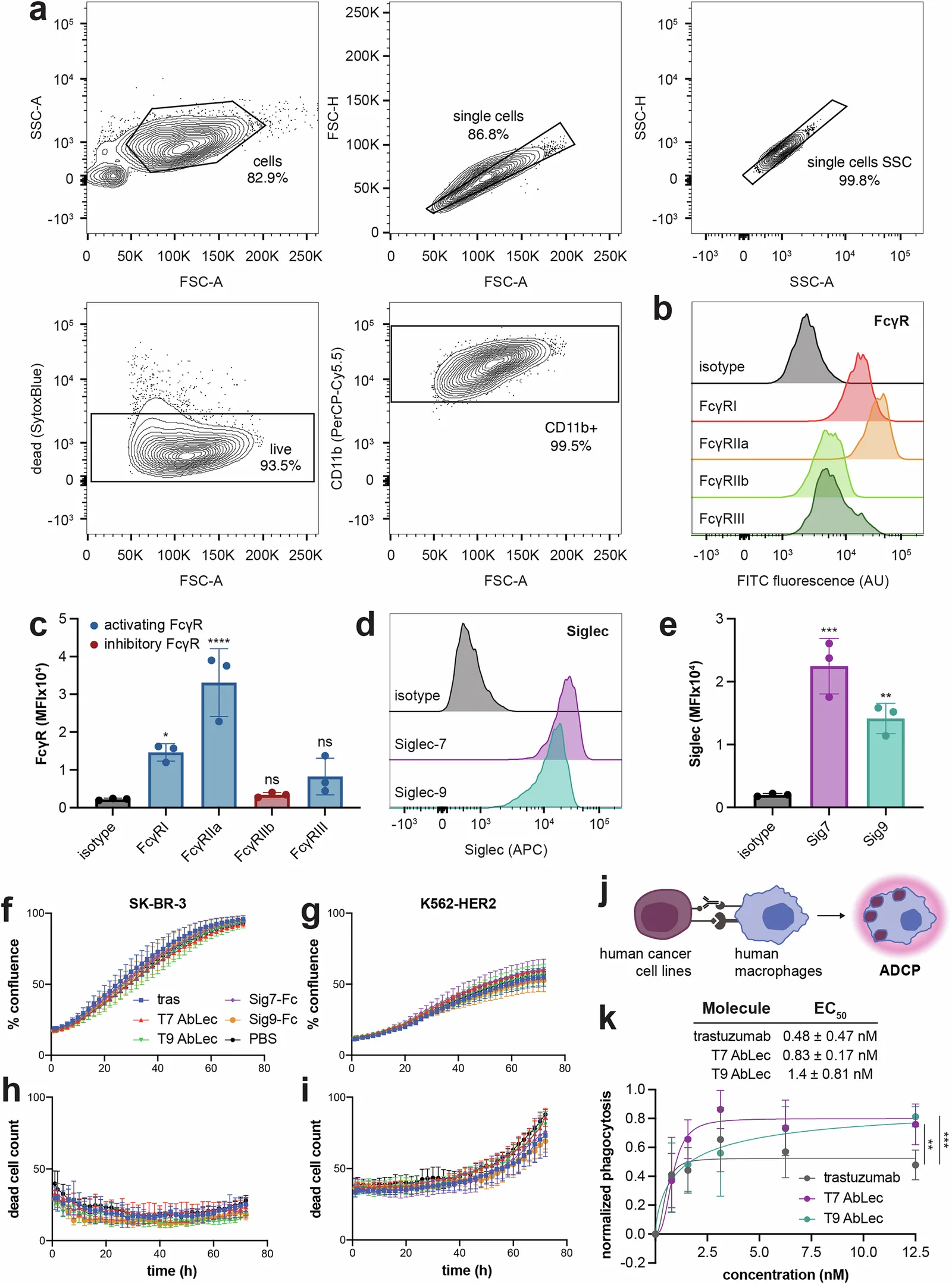
AbLecs elicit enhanced macrophage ADCP *in vitro.* (**a**) Macrophages were defined as CD11b+ by flow cytometry. (**b**) Macrophages were profiled for expression of human Fc gamma receptors FcγRI, FcγRIIa, FcγRIIb, and FcγRIII by flow cytometry. Histograms are representative of macrophages from n = 3 human donors (quantified in **c**). (**c**) Expression of FcγRI and FcγRIIa on macrophages measured via flow cytometry was significant compared to staining with an isotype antibody. Data are mean ± s.d. from n = 3 human donors. (**d**) Macrophages expressed Siglec-7 and Siglec-9 receptors. Histograms are representative of macrophages from n = 3 human donors (quantified in **e**). (**e**) Expression of Siglec-7 and Siglec-9 receptors on macrophages measured via flow cytometry was significant compared to staining with an isotype antibody. Data are mean ± s.d. from n = 3 human donors. (**f-i**) No statistically significant difference in cell growth (**f, g**) or cell death (**h, i**) was observed in SK-BR-3 or K562-HER2 cultures treated with T7 or T9 AbLecs compared to vehicle, trastuzumab, or Siglec-7/9-Fc controls. Data are mean ± s.d. of n = 3 biological replicates. (**j**) Macrophages were co-cultured with human cancer cell lines labeled with the pH-sensitive pHrodo red dye that fluoresces red in acidic phagosomes. This enabled quantification of phagocytosis via fluorescence microscopy. (**k**) Quantification of macrophage phagocytosis of K562-HER2 cells. Co-cultures were treated with increasing concentrations of T7/T9 AbLec or trastuzumab. EC_50_ values ± 95% confidence intervals for trastuzumab and T7/9 AbLecs were determined by fitting experimental data to a four-parameter [agonist] vs response curve. Data are mean ± s.d. of n = 6 (T9 AbLec) or n = 9 (T7 AbLec, trastuzumab) biological replicates. *P*-values were determined by Tukey-corrected one-way ANOVA (**c**, **e**, **k**); ns p>0.05, *p<0.05, **p<0.01, ***p<0.001, and ****p<0.0001. Source data

**Extended Data Fig. 3:**
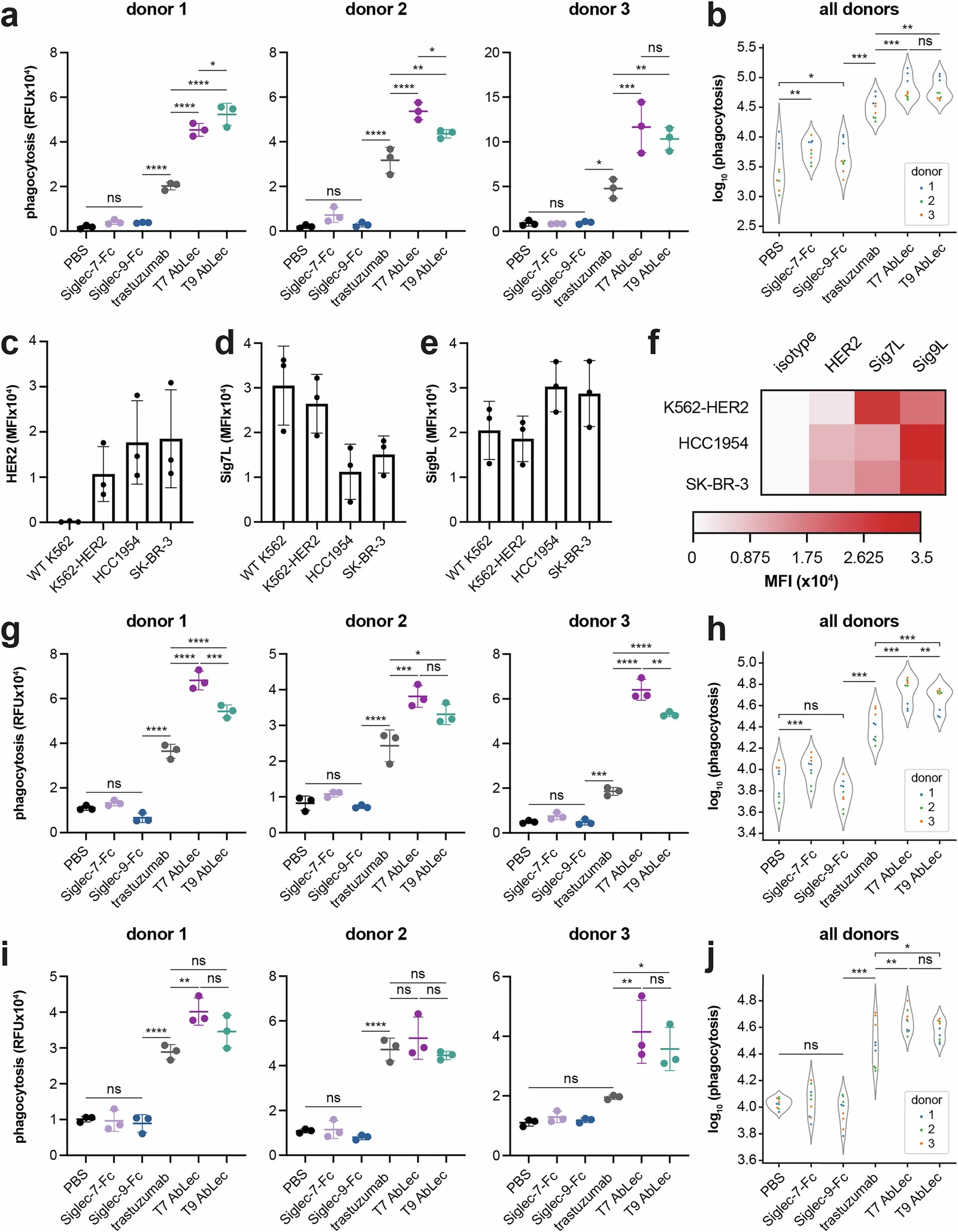
AbLec-mediated enhancement of ADCP is observed across cell lines with varying levels of HER2 and Siglec ligand expression. (**a**) ADCP of SK-BR-3 cells by primary human macrophages treated with T7/9 AbLecs, trastuzumab, or Siglec-7/9-Fc. Donor 3 data are also shown in Fig. 2c. Data are mean ± s.d. of n = 3 biological replicates. (**b**) Statistical analysis of experimental data from (**a**) was performed using Heirarch^143^ to calculate significant differences in treatment conditions across donors (n = 3 biological replicates from each of n = 3 donors). (**c-e**) K562-HER2, HCC-1954, and SK-BR-3 cells express varying levels and ratios of the HER2 antigen (**c**), Sig7L (**d**), and Sig9L (**e**) as measured via flow cytometry. Data are mean ± s.d. from n = 3 biological replicates. (**f**) Heat map representation of average HER2 and Sig7/9L expression measured in (**c-e**) in K562-HER2, HCC-1954, and SK-BR-3 cell lines. (**g**) ADCP of K562-HER2 cells by primary human macrophages treated with T7/9 AbLecs, trastuzumab, or Siglec-7/9-Fc. Data are mean ± s.d. of n = 3 biological replicates. (**h**) Statistical analysis of experimental data from (**g**) was performed using Heirarch^143^ to calculate significant differences in treatment conditions across donors (n = 3 biological replicates from each of n = 3 donors). (**i**) ADCP of HCC-1954 cells by primary human macrophages treated with T7/9 AbLecs, trastuzumab, or Siglec-7/9-Fc. Data are mean ± s.d. of n = 3 biological replicates. (**j**) Statistical analysis of experimental data from (**i**) was performed using Heirarch^143^ to calculate significant differences in treatment conditions across donors (n = 3 biological replicates from each of n = 3 donors). *P*-values were determined by Tukey-corrected one-way ANOVA (**a**, **g**, **i**), or Heirarch’s many-sample, multiple hypothesis test with Benjamini-Hochberg multiple comparisons correction (**b**, **h**, **j**); ns p>0.05, *p < 0.05, **p < 0.01, ***p < 0.001, ****p<0.0001. Source data

We first tested our hypothesis using in vitro phagocytosis assays with primary human macrophages. At the time of use in our assays, macrophages expressed FcγRI and FcγRIIa, as well as Siglec-7 and Siglec-9 (Extended Data Fig. 2a–e). Notably, AbLecs alone are not cytotoxic: we did not observe any defects in SK-BR-3 or K562-HER2 cell growth over 72 h following AbLec treatment (Extended Data Fig. 2f–i). We co-cultured these macrophages with SK-BR-3 cells labelled with a pH-sensitive dye that fluoresces red in acidic phagosomes (Extended Data Fig. 2j), enabling quantification of phagocytosis using fluorescence. After 5 h of co-culture, fluorescence microscopy images show that SK-BR-3 cells in co-cultures treated with the T7 or T9 AbLec are more readily phagocytosed by macrophages than those treated with trastuzumab or Siglec-7/9-Fc decoy receptor (Fig. 2b,c and Supplementary Videos 1–3). We observed the same enhancement of phagocytosis using macrophages from three donors and in assays with the HCC-1854 and K562-HER2 cell lines, which express varying levels of HER2 and/or Sig7L/Sig9L compared to SK-BR-3 cells (Extended Data Fig. 2k and Extended Data Fig. 3).

**Extended Data Fig. 4:**
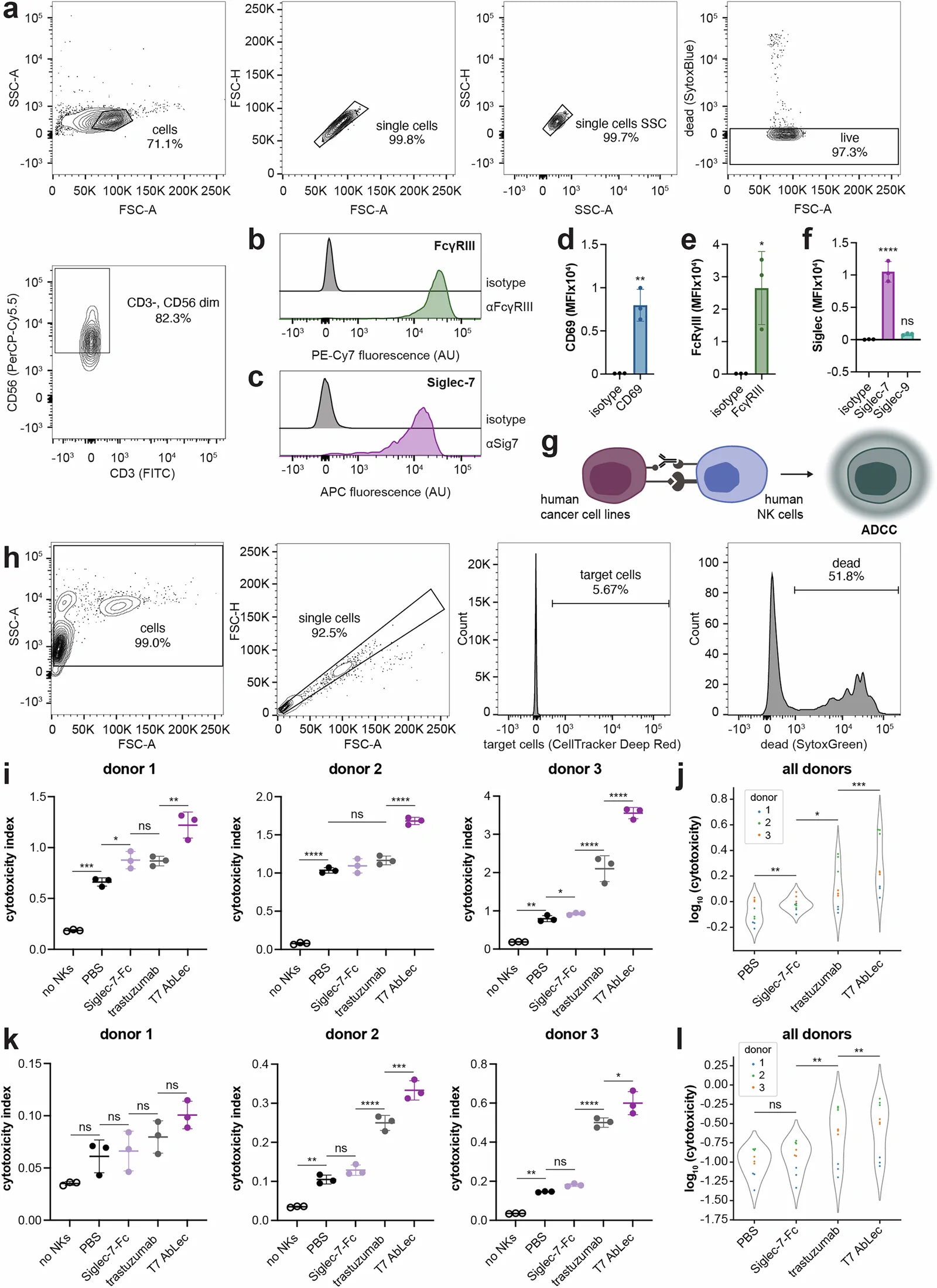
AbLecs elicit enhanced NK cell ADCC *in vitro.* (**a**) NK cells were defined as CD3- CD56 dim by flow cytometry. (**b-c**) NK cells were profiled for expression of FcγRIII (**b**) and Siglec-7 (**c**) by flow cytometry. Histograms are representative of NK cells from n = 3 human donors (quantified in **e, f**). (**d-f**) Quantification of CD69 (activation marker; **d**), FcγRIII (**e**), and Siglec-7 (**f**) expression on NK cells via flow cytometry compared to staining with an isotype antibody. Data are mean ± s.d. from n = 3 human donors (representative histograms shown in **b, c**). (**g**) We co-cultured primary human NK cells with CellTracker Deep Red-labeled human cancer cell lines in the presence of the Sytox Green DNA intercalating dye that permeates the compromised membranes of dying cells, enabling quantification of NK cell killing of target cancer cells via flow cytometry. (**h**) Gating strategy for quantification of NK cell killing of target cancer cells via flow cytometry. (**i**) ADCC of SK-BR-3 cells by NK cells treated with T7/9 AbLecs, trastuzumab, or Siglec-7/9-Fc. Donor 3 data are also shown in Fig. 2d. Data are mean ± s.d. of n = 3 biological replicates. (**j**) Statistical analysis of experimental data from (**i**) was performed using Heirarch^143^ to calculate significant differences in treatment conditions across donors (n = 3 biological replicates from each of n = 3 donors). (**k**) ADCC of K562-HER2 cells by NK cells treated with T7/9 AbLecs, trastuzumab, or Siglec-7/9-Fc. Data are mean ± s.d. of n = 3 biological replicates. (**l**) Statistical analysis of experimental data from (**k**) was performed using Heirarch^143^ to calculate significant differences in treatment conditions across donors (n = 3 biological replicates from each of n = 3 donors). *P*-values were determined by two-tailed unpaired t test (**d**, **e**), Tukey-corrected one-way ANOVA (**f**, **i**, **k**), or Heirarch’s many-sample, multiple hypothesis test with Benjamini-Hochberg multiple comparisons correction (**j**, **l**); ns p>0.05, *p < 0.05, **p < 0.01, ***p < 0.001, ****p<0.0001. Source data

**Extended Data Fig. 5:**
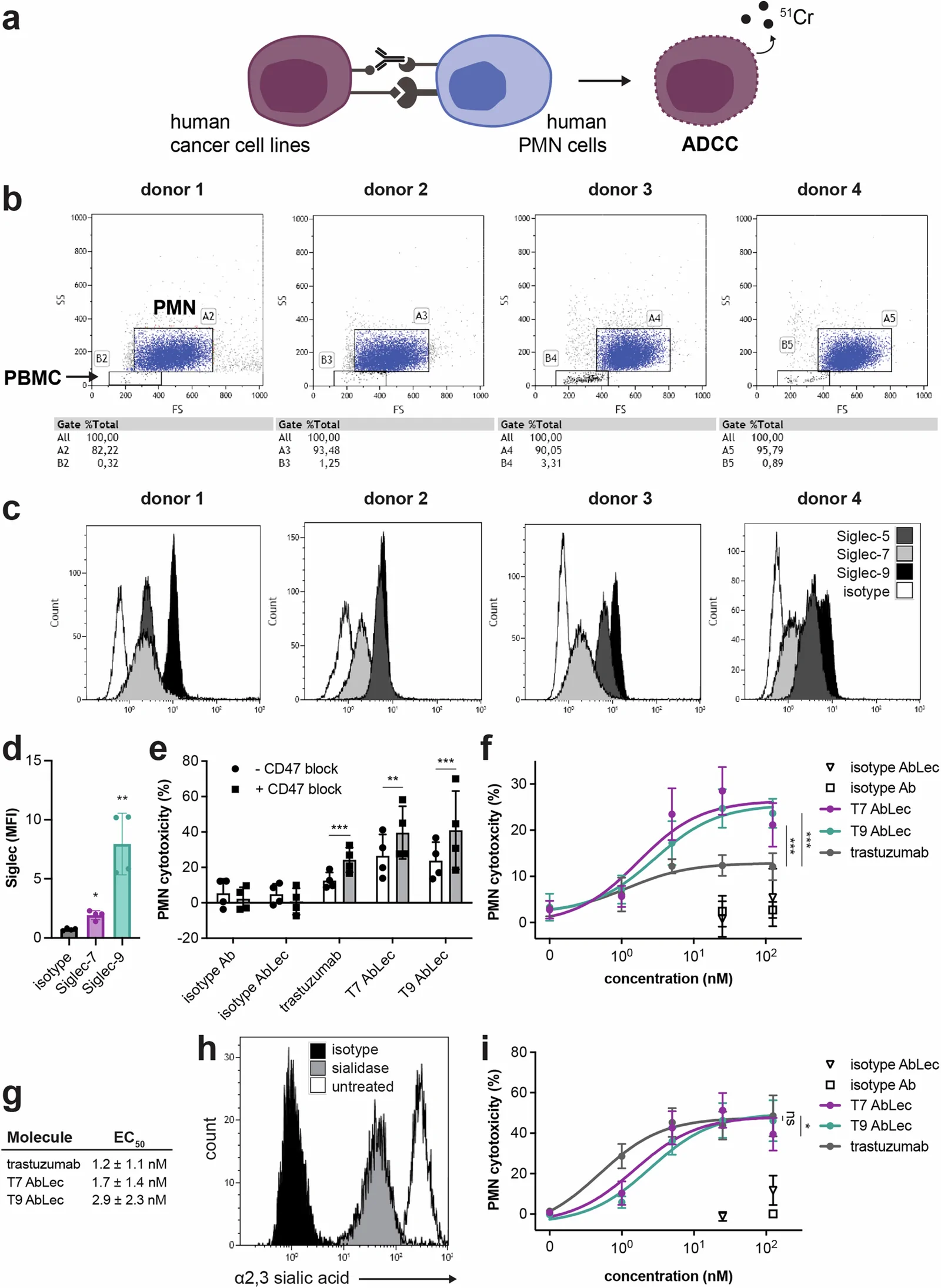
AbLecs elicit enhanced PMN ADCC *in vitro.* (**a**) Primary human granulocytes (PMNs) were co-cultured with SK-BR-3 cells labeled with radioactive chromium (^51^Cr). Tumor cell lysis is quantified by measuring chromium in the supernatant released by dying cells. (**b**) PMNs were defined by distinctive forward and side scatter profiles compared to peripheral blood mononuclear cells (PBMCs). (**c**) PMNs from n = 4 human donors expressed the Siglec-5, -7, and -9 receptors. (**d**) Quantification of Siglec-7 and -9 expression on PMNs. Data are mean ± s.d. from n = 4 human donors. (**e**) Quantification of PMN ADCC of SK-BR-3 cells. Co-cultures were treated with T7/T9 AbLec, trastuzumab, isotype antibody (rituximab), or isotype AbLec (rituximab x Siglec-7 AbLec) controls alone or in combination with a CD47 antagonist antibody. Data are mean ± s.d. of replicates from n = 5 human donors. (**f**) Quantification of PMN ADCC against SK-BR-3 cells. Co-cultures were treated with increasing concentrations of T7/T9 AbLec, trastuzumab, isotype antibody (rituximab), or isotype AbLec (rituximab x Siglec-7 AbLec) controls. Experimental data were fit using sigmoidal dose response curves. Data are mean ± s.d. of replicates from n = 5 human donors. (**g**) EC_50_ values ± 95% confidence intervals for trastuzumab and T7/9 AbLecs were determined by fitting experimental data from (**f**) to sigmoidal dose response curves. (**h**) Cell surface α2,3 linked sialic acid expression measured via staining sialidase treated or untreated SK-BR-3 cells with the MALII lectin that detects α2,3 linked sialic acid and analysis by flow cytometry. (**i**) Quantification of PMN ADCC against sialidase treated SK-BR-3 cells. Co-cultures were treated with increasing concentrations of T7/T9 AbLec, trastuzumab, isotype antibody (rituximab), or isotype AbLec (rituximab x Siglec-7 AbLec) controls. Experimental data were fit using sigmoidal dose response curves. Data are mean ± s.d. of replicates from n = 5 human donors. *P*-values were determined by two-tailed unpaired t test (**d**) or Heirarch’s many-sample, multiple hypothesis test with Benjamini-Hochberg multiple comparisons correction (**e**, **f**, **i**); ns p>0.05, *p < 0.05, **p < 0.01, ***p < 0.001, ****p<0.0001. Source data

We next asked whether AbLecs could potentiate cancer cell killing by additional subsets of immune cells. We co-cultured primary human NK cells expressing FcγRIII and Siglec-7 with SK-BR-3 tumour cells and analysed cytotoxicity by flow cytometry (Extended Data Fig. 4a–h). Across three donors, the T7 AbLec significantly enhanced NK cell cytotoxicity compared to trastuzumab or Siglec-7-Fc decoy receptor (Fig. 2d and Extended Data Fig. 4i,j). We observed the same effect in assays with the K562-HER2 cell line (Extended Data Fig. 4k,l). We performed similar assays with polymorphonuclear leukocytes (PMNs), which express FcγRIIa and FcγRIII^29^ as well as Siglec-7 and Siglec-9 (Extended Data Fig. 5a–d). We found that T7 and T9 AbLecs elicited increased cancer cell killing by PMNs compared to trastuzumab and AbLec isotype controls (Fig. 2e and Extended Data Fig. 5e–i).

**Extended Data Fig. 6:**
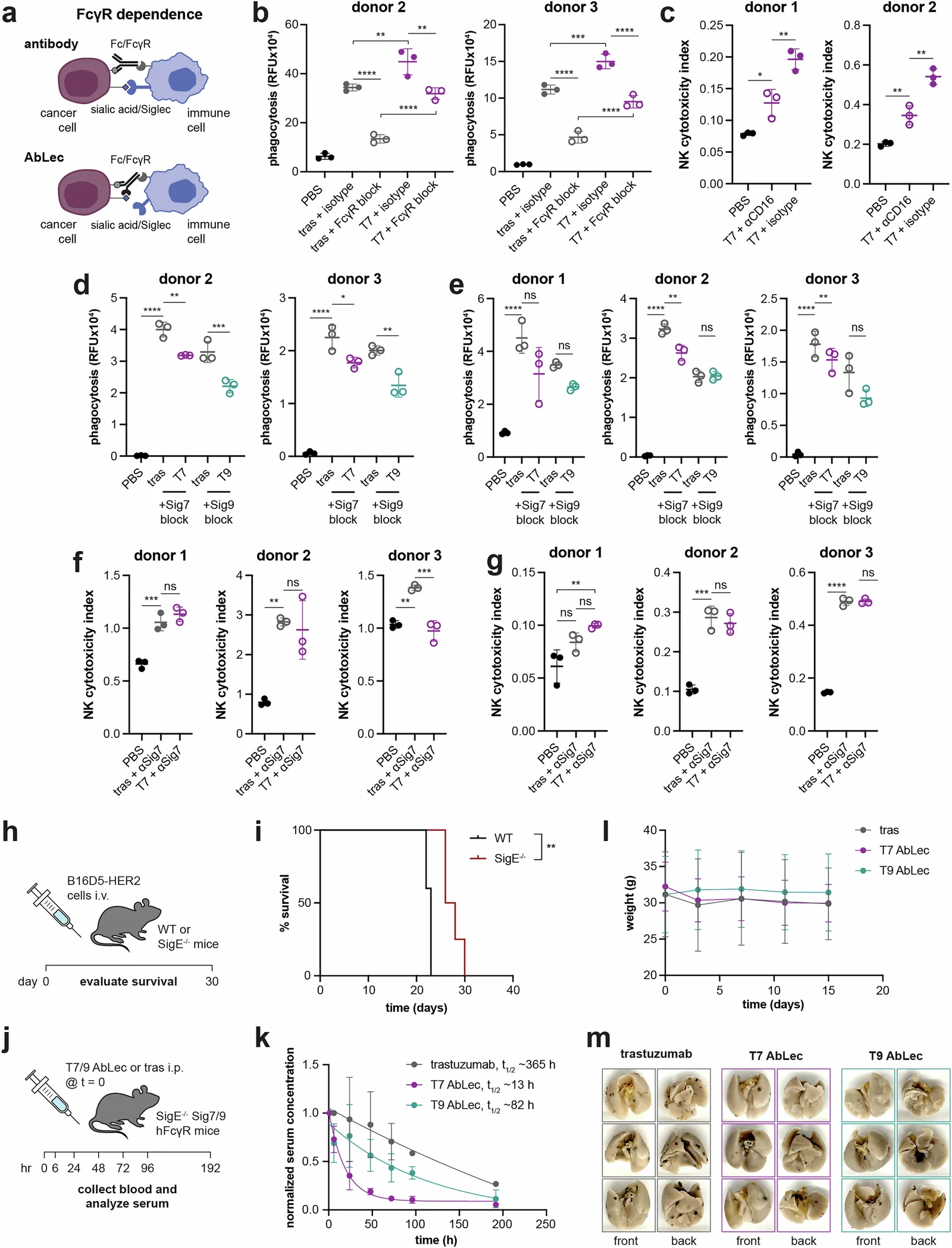
AbLecs enhance immune control of cancer *in vitro* and *in vivo* via a dual mechanism of action. (**a**) We used Fc gamma receptor (FcγR) blocking antibodies to assess the extent to which trastuzumab and T7 AbLec activity are FcγR-dependent. (**b**) Primary human macrophages were pre-treated with FcγR blocking or isotype control antibodies prior to co-culture with SK-BR-3 cells and treatment with trastuzumab (tras) or T7 AbLec. Data are mean ± s.d. of n = 3 biological replicates. (**c**) Primary human NK cells were pre-treated with an FcγRIII/CD16-blocking antibody (clone 3G8; αCD16) or an isotype control antibody prior to co-culture with SK-BR-3 cells and treatment with T7 AbLec. Data are mean ± s.d. of n = 3 biological replicates. (**d, e**) Macrophage ADCP of SK-BR-3 (**d**) or K562-HER2 (**e**) cells induced by trastuzumab or T7/9 AbLecs in the presence of Siglec-7/9 blocking antibodies (αSig7 or αSig9, respectively). Primary human macrophages were pre-treated with αSig7 or αSig9 prior to co-culture with cancer cells. Data are mean ± s.d. of n = 3 biological replicates. (**f, g**) NK cell ADCC of SK-BR-3 (**f**) or K562-HER2 (**g**) cells induced by trastuzumab or T7 AbLec in the presence or absence of a Siglec-7 blocking antibody (αSig7). Primary human NK cells were pre-treated with αSig7 prior to co-culture with cancer cells. Data are mean ± s.d. of n = 3 biological replicates. (**h**) We used SigE^−/−^ mice to understand the therapeutic effect of Siglec blockade in a model of metastatic cancer. WT or SigE^−/−^ mice were inoculated intravenously (i.v.) with B16-HER2 cancer cells and mice were monitored over a period of 30 days. (**i**) Overall survival of n = 5 WT (black) and n = 4 SigE^−/−^ (red) mice inoculated i.v. with B16-HER2 cells. (**j**) We assessed pharmacokinetics of trastuzumab and T7/9 AbLecs in SigE^−/−^ Sig7/9 hFcγR mice by monitoring serum concentration for each molecule at t = 6, 24, 48, 72, and 96 hr after intraperitoneal (i.p.) injection. (**k**) Concentration of trastuzumab and T7/9 AbLecs in serum of SigE^−/−^ Sig7/9 hFcγR mice following i.p. injection at t = 0 hr and normalized to serum concentration at t = 6 hr. The circulating half-life (t_1/2_) for each molecule was estimated by fitting experimental data to a one phase decay curve. Data are mean ± s.e. from n = 2 biological replicates. (**l**) Weights of SigE^−/−^ Sig7/9 hFcγR mice used in Fig. 2o, p were recorded every 3–4 days during the study. Data are mean ± s.d. from n = 7 (T7/9 AbLec) or n = 8 (trastuzumab) biological replicates. (**m**) Representative images of lungs of SigE^−/−^ Sig7/9 hFcγR mice inoculated with B16-HER2 cells i.v. and treated with trastuzumab or T7/9 AbLecs. Images are representative of n = 7 (T7/9 AbLec) or n = 8 (trastuzumab) biological replicates, quantified in Fig. 2p. *P*-values were determined by Tukey-corrected one-way ANOVA (**b-g**) or log-rank (Mantel-Cox) test (**i**); ns p>0.05, *p<0.05, **p<0.01, ***p<0.001, and ****p<0.0001. Source data

It was important to demonstrate that AbLec activity is at least partially antibody-dependent; that is, mediated by antigen binding and FcγR engagement. To test FcγR dependence, we incubated macrophages with FcγR blocking or isotype control antibodies before co-culture with SK-BR-3 cells. We observed lower levels of SK-BR-3 phagocytosis for both trastuzumab and T7 AbLec when macrophages were pre-treated with FcγR blocking antibodies, indicating that both antibody and AbLec activities are FcγR-dependent (Fig. 2f and Extended Data Fig. 6a,b). We further found that an antibody blocking FcγRIII/CD16 significantly reduced AbLec-mediated ADCC by NK cells, confirming that AbLec activity is FcγR-dependent (Extended Data Fig. 6c). Notably, FcγR blockade appeared to reduce trastuzumab-mediated phagocytosis to a greater extent than the T7 AbLec. Indeed, when we quantified the reduction in phagocytosis observed upon FcγR blockade across three donors, we found that trastuzumab exhibited a greater dependence on FcγRs than the T7 AbLec (Fig. 2g). These data support our hypothesis that the AbLec mechanism of action is bifunctional, with contributions from activation of antibody effector functions and glyco-immune checkpoint blockade. We further observed that T7/9 AbLec-mediated phagocytosis of target cells was dependent on expression of the HER2 antigen in phagocytosis assays with wild-type (HER2^−^) K562 and K562-HER2 cells (Fig. 2h). Overall, these results show that T7 and T9 AbLecs elicit enhanced antibody effector functions, including ADCP and ADCC, compared to the parent monoclonal antibody, trastuzumab.

We tested whether the AbLec-mediated enhancement of anticancer immune responses was a result of blockade of Siglec–sialoglycan interactions. In one approach, we pre-incubated macrophages or NK cells with Siglec-7 or Siglec-9 blocking antibodies before co-culture with SK-BR-3 or K562-HER2 cells (Fig. 2i). When the targeted Siglec receptor was blocked, AbLecs and trastuzumab elicited similar levels of ADCP (Fig. 2j and Extended Data Fig. 6d,e) and ADCC (Extended Data Fig. 6f,g), suggesting that AbLec-mediated enhancement of antibody effector functions is Siglec-dependent. Similarly, pretreatment of target cells with sialidase to degrade Siglec ligands also abolished AbLec-mediated enhancement of PMN ADCC over trastuzumab (Fig. 2k,l and Extended Data Fig. 5f,h,i), further supporting the idea that AbLec activity is Siglec-dependent. These results help to rule out any contribution of knobs-in-hole Fc engineering to enhancement of ADCP and ADCC, consistent with previous studies showing that these mutations preserve the native structure and effector functions of wild-type hIgG1 Fc^53,60,61^. Taken together, these results suggest that the increased levels of ADCP and ADCC observed with AbLec treatment are a direct result of glycan blockade.

### AbLec treatment reduces tumour burden in vivo

We next set out to investigate whether AbLecs could improve immune control of cancer in vivo. However, we faced the challenge that Siglec biology is not conserved from mice to humans. Human Siglec-7 and Siglec-9 share a common murine ortholog, Siglec-E, but neither expression patterns on immune cells nor binding preferences of Siglec-7 or Siglec-9 completely match those of Siglec-E^62^. To address this gap, we leveraged recently developed humanized mice in which the *Siglece* gene is inactivated and *SIGLEC7* and *SIGLEC9* transgenes are stably expressed under the control of their endogenous promoters^20^. As a result, the expression patterns of Siglec-7 and Siglec-9 on leucocytes in these mice closely mirror those on human peripheral blood mononuclear cells (PBMCs)^20^. We crossed these mice with established humanized FcγR mice^63^ to yield animals that recapitulate both human Siglec-7/9 and FcγR biology that together mediate the dual mechanism of action of T7/9 AbLecs, providing the ideal genetic background for evaluation of in vivo efficacy. We established a lung colonization model by intravenous inoculation of syngeneic B16D5-HER2 cancer cells, which express the HER2 antigen, high levels of Sig9L and intermediate levels of Sig7L (Fig. 2m,n), and found that this model exhibited Siglec-dependent progression (Extended Data Fig. 6h,i). We therefore set out to evaluate the effect of T7 and T9 AbLec in this model, using a dosing strategy informed by their half-lives in circulation in our humanized Siglec-7/9 hFcγR mice (Fig. 2o and Extended Data Fig. 6j,k). We found significantly reduced metastatic burden in the lungs of mice treated with T9 AbLec compared to those treated with the parent antibody, trastuzumab (Fig. 2p and Extended Data Fig. 6l,m). Metastatic burden was overall lower in T7 AbLec-treated mice than in trastuzumab-treated mice, but this difference was not statistically significant (Fig. 2p and Extended Data Fig. 6l,m). We speculate that Siglec-9 may be the dominant glyco-immune checkpoint in our lung colonization model, owing to the relatively low level of Sig7L compared to Sig9L expression on the B16D5-HER2 cell line and the fact that neutrophils, which express higher levels of Siglec-9 than Siglec-7, are the dominant Siglec-expressing immune cell population in the lung^20^. Overall, AbLec treatment reduced tumour burden in a translationally relevant animal model.

### The chimeric AbLec architecture mediates potent glyco-immune checkpoint antagonism

**Fig. 3:**
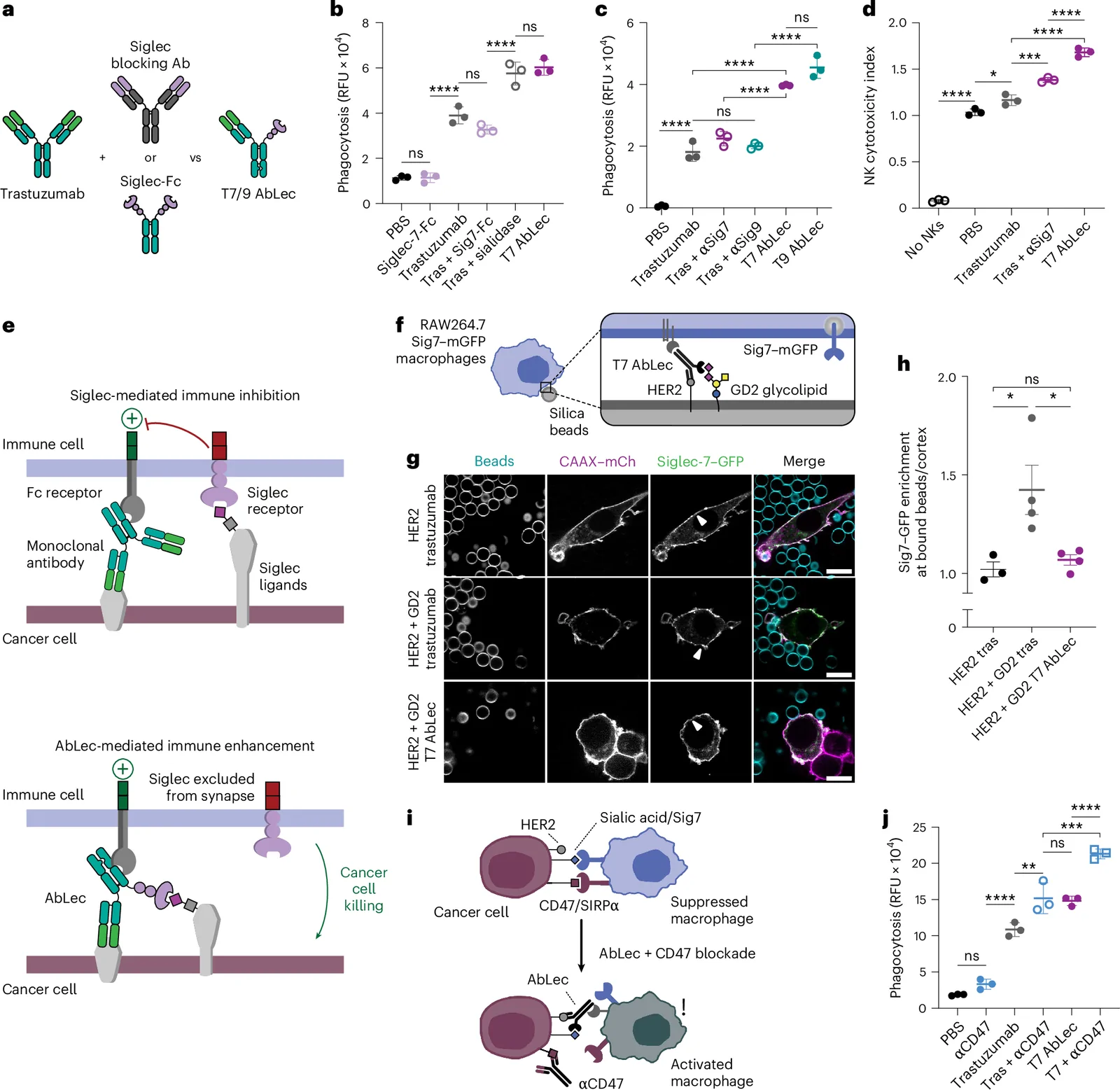
AbLecs act at the immunological synapse to target a distinct axis of immune regulation. **a**, We compared the combination of trastuzumab with Siglec-7/9 blocking antibodies (αSig7 or αSig9, respectively), Siglec-7-Fc or sialidase to T7 AbLec treatment in their ability to activate ADCP and ADCC. **b**, Macrophage ADCP of K562-HER2 cells treated with Siglec-7-Fc, trastuzumab, T7 AbLec or combinations of trastuzumab with Siglec-7-Fc or sialidase. Data are mean ± s.d. of *n* = 3 biological replicates. **c**, Macrophage ADCP of SK-BR-3 cells treated with trastuzumab, T7/9 AbLec or combinations of trastuzumab with Siglec-7/9 blocking antibodies (αSig7 or αSig9, respectively). Data are mean ± s.d. of *n* = 3 biological replicates. **d**, NK cell ADCC of SK-BR-3 cells treated with trastuzumab, T7 AbLec or a combination of trastuzumab with Siglec-7 blocking antibody (αSig7). Data are mean ± s.d. of *n* = 3 biological replicates. **e**, We reasoned that the chimeric AbLec architecture simultaneously elicits antibody effector functions through FcγR engagement and blocks recruitment of Siglecs to the immunological synapses formed. **f**, RAW264.7 cells were engineered to express a Siglec-7–mGFP fusion (RAW264.7-Siglec-7–mGFP macrophages) and co-cultured with silica beads functionalized with HER2 and the GD2 glycolipid as a model Siglec-7 ligand. Immune synapses can be formed and imaged by opsonization with trastuzumab or T7 AbLec. **g**, Representative images of macrophages bound to trastuzumab-opsonized or T7 AbLec-opsonized HER2^+^GD2^+^ silica beads showing Siglec-7, beads and membrane-tethered mCherry (CAAX–mCh) to quantify membrane thickness. Arrows indicate examples of synapses at the cell–bead interface. Images are representative of *n* = 22 (HER2 tras), *n* = 37 (HER2 + GD2 tras) or *n* = 34 (HER2 + GD2 T7 AbLec) synapses quantified across *n* = 3 (HER2 tras) or *n* = 4 (HER2 + GD2 tras, HER2 + GD2 T7 AbLec) biological replicates. Scale bar, 10 μm. Images of the same synapses at 5 μm resolution are shown in Extended Data Fig. 8i. In merged fluorescence images, Siglec-7–GFP signal is shown in green, CAAX–mCh signal is shown in red and bead signal is shown in teal. **h**, Enrichment of Siglec-7 to bound beads was quantified as the ratio of GFP fluorescence at the synapse (for example, arrows in **g**) compared to the adjacent membrane cortex for the indicated macrophage–bead co-cultures. Data are mean ± s.d. of *n* = 22 (HER2 tras), *n* = 37 (HER2 + GD2 tras) or *n* = 34 (HER2 + GD2 T7 AbLec) synapses quantified across *n* = 3 (HER2 tras) or *n* = 4 (HER2 + GD2 tras, HER2 + GD2 T7 AbLec) biological replicates. **i**, We tested the ability of T7 AbLec treatment to synergize with CD47 blockade in ADCP assays with primary human macrophages. **j**, Macrophage ADCP of SK-BR-3 cells treated with trastuzumab or T7 AbLec alone and in combination with a CD47 antagonist antibody. Data are mean ± s.d. of *n* = 3 biological replicates. *P* values were determined by Tukey-corrected one-way ANOVA (**b**–**d**, **h**, **j**); ns, not significant (*P* > 0.05); *P < 0.05; **P < 0.01; ***P < 0.001; ****P < 0.0001. Source data

**Extended Data Fig. 7:**
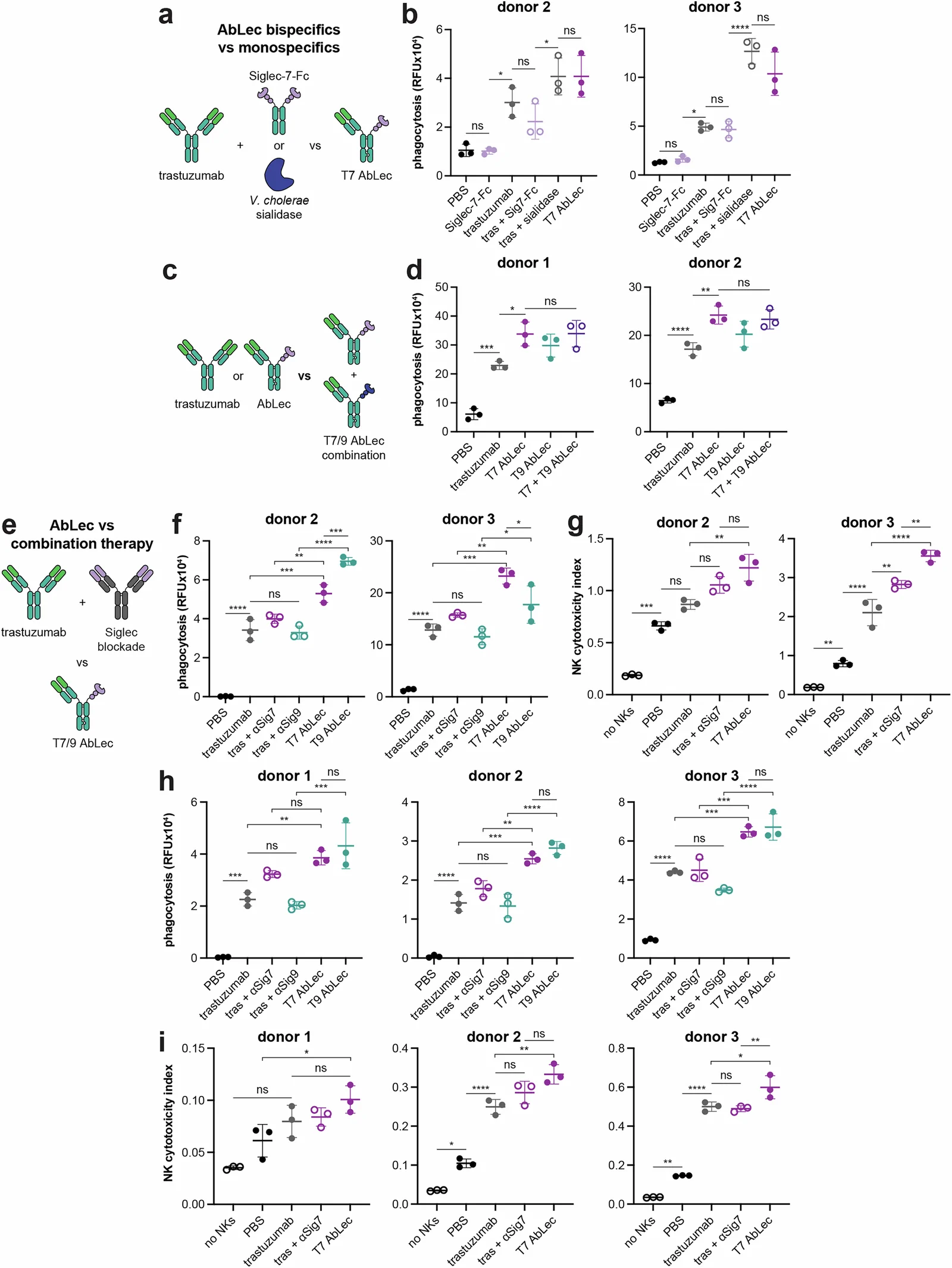
AbLecs outperform combinations of monospecific immunotherapies. (**a**) We compared combination of trastuzumab with Siglec-7-Fc or sialidase to T7 AbLec treatment in ADCP assays to test whether the chimeric AbLec architecture was required for efficacy. (**b**) Macrophage ADCP of K562-HER2 cells treated with Siglec-7-Fc, trastuzumab, T7 AbLec, or combinations of trastuzumab with Siglec-7-Fc or sialidase. Data are mean ± s.d. of n = 3 biological replicates. (**c**) We asked whether combination of two AbLecs targeting distinct glyco-immune checkpoints could further enhance immune cell activation. (**d**) Macrophage ADCP of SK-BR-3 cells treated with equimolar concentrations of trastuzumab, T7 AbLec, T9 AbLec, or the combination of T7 and T9 AbLecs. Data are mean ± s.d. of n = 3 biological replicates. (**e**) We compared combinations of trastuzumab with Siglec-7/9 blocking antibodies (αSig7 or αSig9, respectively) to T7 or T9 AbLec treatment alone in ADCC and ADCP assays. (**f**) Macrophage ADCP of SK-BR-3 cells treated with trastuzumab, T7/9 AbLec, or combinations of trastuzumab with Siglec-7/9 blocking antibodies (αSig7 or αSig9, respectively). Data are mean ± s.d. of n = 3 biological replicates. (**g**) NK cell ADCC of SK-BR-3 cells treated with trastuzumab, T7 AbLec, or combination of trastuzumab with Siglec-7 blocking antibody (αSig7). Data are mean ± s.d. of n = 3 biological replicates. (**h**) Macrophage ADCP of K562-HER2 cells treated with trastuzumab, T7/9 AbLec, or combinations of trastuzumab with Siglec-7/9 blocking antibodies (αSig7 or αSig9, respectively). Data are mean ± s.d. of n = 3 biological replicates. (**i**) NK cell ADCC of K562-HER2 cells treated with trastuzumab, T7 AbLec, or combination of trastuzumab with Siglec-7 blocking antibody (αSig7). Data are mean ± s.d. of n = 3 biological replicates. *P*-values were determined by Tukey-corrected one-way ANOVA (**b**, **d**, **f-i**); ns p>0.05, *p < 0.05, **p < 0.01, ***p < 0.001, ****p<0.0001. Source data

We were curious to know whether the chimeric AbLec architecture was required to potentiate killing of cancer cells. To determine whether this was the case, we compared ADCP of K562-HER2 cells treated with T7 AbLec or the combination of trastuzumab and the Siglec-7-Fc decoy receptor (Fig. 3a and Extended Data Fig. 7a). We found that the combination of trastuzumab and Siglec-7-Fc did not enhance killing of K562-HER2 cells compared to trastuzumab alone, as expected given the low binding affinity of the decoy receptor (Fig. 3b and Extended Data Fig. 7b). However, as before, treatment with T7 AbLec elicited enhanced cancer cell phagocytosis (Fig. 3b and Extended Data Fig. 7b). From these results, we conclude that the chimeric AbLec architecture is required to recruit lectin decoy receptor domains to cancer cell surfaces at therapeutically relevant concentrations and potentiate their destruction.

Combinations of monospecific therapies can result in enhanced therapeutic efficacy and combinatorial treatment strategies are now thought to be required for durable responses in most patients with cancer^64^. We hoped that the bispecific AbLec architecture would elicit tumour-targeted antibody effector functions and glyco-immune checkpoint blockade at least as effectively as combination therapy. Therefore, we sought to benchmark the efficacy of AbLecs compared to combinations of trastuzumab with existing Siglec blockade approaches. First, we compared AbLec efficacy to a combination of trastuzumab with sialidase as a model of enzymatic sialoside degradation, an approach to target the Siglec–sialoglycan axis that is currently being investigated in clinical trials^41^ (Extended Data Fig. 7a). There was no significant difference in ADCP elicited by T7 AbLec treatment compared to the combination of trastuzumab with sialidase (Fig. 3b and Extended Data Fig. 7b). The combination of T7 and T9 AbLecs also did not further enhance ADCP compared to T7 or T9 treatment alone (Extended Data Fig. 7c,d). These data indicate that AbLec-mediated Siglec blockade is as effective as sialic acid degradation in its ability to potentiate antibody effector functions.

In addition, we compared AbLec treatment to combinations of trastuzumab with Siglec-blocking antibodies^20^ (Fig. 3a and Extended Data Fig. 7e). Notably, AbLecs elicited enhanced ADCP (Fig. 3c and Extended Data Fig. 7f) and ADCC (Fig. 3d and Extended Data Fig. 7g) of SK-BR-3 cells compared to the combination of trastuzumab with Siglec-7 or Siglec-9 antagonist antibodies^20^. We observed the same trends in ADCP and ADCC assays with K562-HER2 cells (Extended Data Fig. 7h,i). These data suggest that blockade of Siglec engagement locally, at the immune synapse, could be more effective than systemic Siglec antagonism.

Overall, our findings support the paradigm that the chimeric AbLec architecture results in a gain-of-function: the ability to block inhibitory signalling through glyco-immune checkpoints. AbLecs are more effective glyco-immune checkpoint antagonists than Siglec-blocking antibodies, and as effective as enzymatic sialoglycan degradation, both of which are therapeutic strategies under investigation in clinical trials^40,41^.

### Siglec recruitment to immunological synapses inhibits phagocytosis

**Extended Data Fig. 8:**
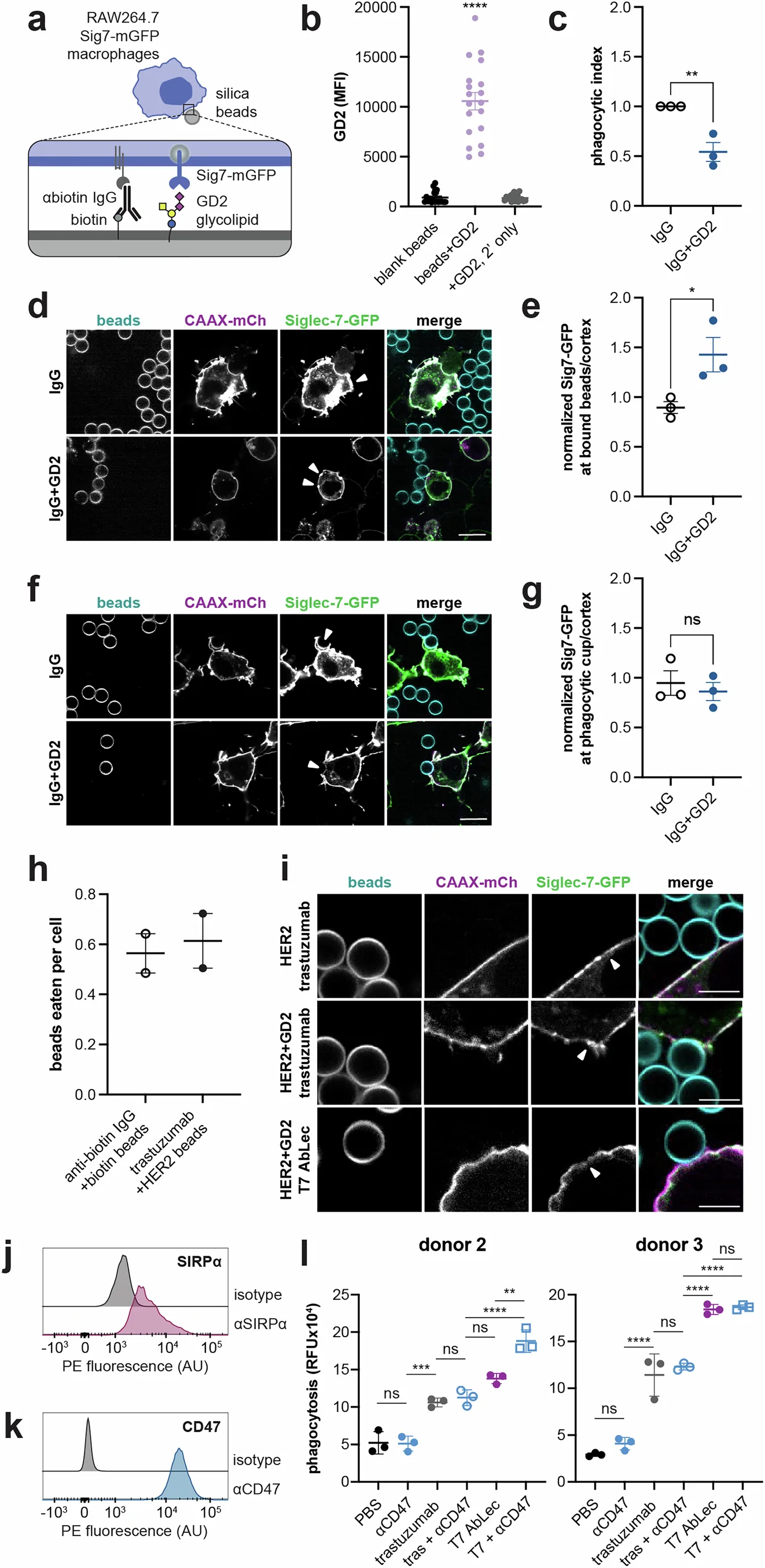
AbLecs prevent Siglec engagement at phagocytic synapses and synergize with blockade of established immune checkpoints. (**a**) RAW264.7 macrophages were engineered to express a Siglec-7–mGFP fusion (RAW264.7-Siglec-7–mGFP) and co-cultured with silica beads functionalized with biotinylated lipids and the GD2 glycolipid as a model Siglec-7 ligand. Immune synapses can be formed and imaged via opsonization with an anti-biotin antibody. (**b**) Flow cytometry analysis of GD2 on silica beads detected using an anti-GD2 antibody and an anti-human secondary antibody. Data are mean ± s.d. of n = 19 biological replicates. (**c**) RAW264.7-Siglec-7–mGFP phagocytosis of biotinylated silica beads with or without the Siglec-7 ligand GD2 opsonized with an anti-biotin antibody (IgG). Each data point represents the mean number of beads eaten per cell across at least 100 RAW264.7-Siglec-7–mGFP macrophages, normalized to the average phagocytosis of beads lacking GD2. Data are mean ± s.d. of n = 3 biological replicates. (**d**) Representative images of macrophages bound to anti-biotin antibody (IgG)-opsonized biotinylated silica beads ± GD2 showing Siglec-7, beads, and membrane-tethered mCherry (CAAX–mCh). Arrows indicate examples of synapses at the cell/bead interface. Images are representative of n = 17 (IgG) or n = 23 (IgG+GD2) bound bead synapses captured across n = 3 biological replicates, scale bar represents 10 μm. In merged fluorescence images, Siglec-7–GFP signal is shown in green, CAAX–mCh signal is shown in red, and bead signal is shown in teal. (**e**) Enrichment of Siglec-7 to bound beads was quantified as the ratio of GFP fluorescence to CAAX–mCh fluorescence at the synapse (for example, arrows in **d**) compared to the adjacent membrane cortex for the indicated macrophage/bead co-cultures. Data are mean ± s.d. of n = 17 (IgG) or n = 23 (IgG+GD2) bound bead synapses quantified across n = 3 biological replicates. (**f**) Representative images of phagocytic synapses with biotinylated silica beads ± GD2 showing Siglec-7, beads, and CAAX–mCh opsonized with an anti-biotin antibody (IgG). Arrows indicate examples of phagocytic cups at the cell/bead interface. Images are representative of n = 24 (IgG) or n = 35 (IgG+GD2) phagocytic synapses imaged across n = 3 biological replicates, scale bar represents 10 μm. In merged fluorescence images, Siglec-7–GFP signal is shown in green, CAAX–mCh signal is shown in red, and bead signal is shown in teal. (**g**) Enrichment of Siglec-7 to phagocytic synapses was quantified as the ratio of Siglec-7–GFP fluorescence to CAAX–mCh fluorescence at the phagocytic cup (for example, arrows in **f**) compared to the adjacent membrane cortex for the indicated macrophage/bead co-cultures. Data are mean ± s.d. of n = 24 (IgG) or n = 35 (IgG+GD2) phagocytic synapses captured across n = 3 biological replicates. (**h**) RAW264.7-Siglec-7–mGFP phagocytosis of biotinylated silica beads opsonized with anti-biotin antibody (IgG) compared to HER2+ silica beads opsonized with trastuzumab. Each data point represents the mean number of beads eaten per cell across at least 100 RAW264.7-Siglec-7–mGFP macrophages. Data are mean ± s.e. of n = 2 biological replicates. (**i**) Representative images of macrophages bound to trastuzumab-opsonized or T7 AbLec-opsonized HER2+ silica beads ± GD2 showing Siglec-7, beads, and CAAX–mCh. Arrows indicate examples of synapses at the cell/bead interface. Images are representative of n = 22 (HER2 tras), n = 37 (HER2 + GD2 tras), or n = 34 (HER2 + GD2 T7 AbLec) bound bead synapses quantified across n = 3 (HER2 tras) or n = 4 (HER2 + GD2 tras, HER2 + GD2 T7 AbLec), scale bar represents 5 μm. Images of the same synapses at 10 μm resolution are shown in Fig. 3g. In merged fluorescence images, Siglec-7-GFP signal is shown in green, CAAX–mCh signal is shown in red, and bead signal is shown in teal. (**j**) Primary human macrophages used in *in vitro* phagocytosis assays express SIRPα by flow cytometry. Histogram is representative of biological replicates from n = 2 donors. (**k**) SK-BR-3 cells express CD47 by flow cytometry. Histogram is representative of n = 2 biological replicates. (**l**) Macrophage ADCP of SK-BR-3 cells treated with αCD47 antagonist antibody, trastuzumab, and T7 AbLec alone and in various combinations. Data are mean ± s.d. of n = 3 biological replicates. *P*-values were determined by Tukey-corrected one-way ANOVA (**b**, **l**) or unpaired, two-tailed student’s t test (**c**, **e**, **g**); ns p>0.05, *p < 0.05, **p < 0.01, ***p < 0.001, ****p<0.0001. Source data

Our surprising finding that AbLecs were more effective Siglec antagonists than Siglec-blocking antibodies suggested that the proximity of the tumour-targeting and Siglec blockade elements may be important. Therefore, we sought to better understand how Siglec–sialoglycan interactions at the immune synapses formed by AbLec opsonization impact macrophage activation. We overexpressed a Siglec-7–mGFP fusion protein in RAW264.7 macrophages, which allowed us to visualize Siglec localization on the cell surface (Extended Data Fig. 8a). We incubated these macrophages with silica beads coated in a supported lipid bilayer that mimiced the surface of a cancer cell and could be functionalized with defined molecular signals (Extended Data Fig. 8a). To activate engulfment, we introduced an anti-biotin antibody, which forms immune complexes with biotinylated lipids in the supported lipid bilayer. We could further functionalize the bilayer with the GD2 glycolipid, a high-affinity ligand for the Siglec-7 receptor^23^ (Extended Data Fig. 8b). Addition of GD2 suppressed bead engulfment, consistent with the results of our ADCP experiments with human primary macrophages and cancer cells (Extended Data Fig. 8c).

With this system in hand, we investigated how Siglec localization was influenced by the presence of its ligand, GD2. We observed significant enrichment of Siglec-7 at immune synapses formed between macrophages and bound beads bearing GD2 (Extended Data Fig. 8d,e). By contrast, there was no enrichment of Siglec-7 at the phagocytic cups of macrophages that had initiated bead engulfment in both the presence and absence of GD2 (Extended Data Fig. 8f,g). Taken together, our results show that ligands like GD2 position Siglecs at the immunological synapse, where they act to inhibit phagocytosis at the earliest stages of target cell encounter.

### AbLecs exclude Siglecs from phagocytic synapses and synergize with CD47 blockade

With these insights into how Siglecs exert their inhibitory functions at the immunological synapse, we next investigated the effect of AbLec treatment on Siglec localization. We suspected that the AbLec decoy receptor arm could block Siglec recruitment to synapses formed by the trastuzumab antibody arm by occupying Siglec ligands in close physical proximity (Fig. 3e). To test this hypothesis, we created target cell mimics by functionalizing silica beads with the HER2 antigen and GD2 as a ligand for Siglec-7, opsonized with trastuzumab or T7 AbLec, and incubated with RAW264.7-Siglec-7–mGFP macrophages (Fig. 3f and Extended Data Fig. 8h). As expected, Siglec-7 was recruited to synapses formed between macrophages and trastuzumab-treated beads bearing the Siglec-7 ligand GD2 (Fig. 3g,h and Extended Data Fig. 8i). By contrast, AbLec treatment completely prevented Siglec-7 accumulation at these synapses; we observed no significant difference in Siglec recruitment to AbLec-treated beads bearing GD2 compared to trastuzumab-treated beads that lacked Sig7L (Fig. 3g,h and Extended Data Fig. 8i). This evidence supports our hypothesis that the ability of tumour-targeted AbLecs to enhance immune cell activation is mediated through exclusion of inhibitory Siglec receptors from the immunological synapse.

Recruitment of SIRPα to phagocytic synapses upon binding to its ligand, the ‘don’t eat me’ signal CD47, similarly suppresses phagocytosis^65,66^. As a result, we wondered whether Siglec–sialoglycan and CD47–SIRPα immune checkpoints are redundant or distinct axes of inhibition in macrophages. We tested this hypothesis by investigating whether trastuzumab-hybrid AbLecs synergize with blockade of CD47, in light of recent results showing that CD47 blockade synergizes with trastuzumab^67,68^. If Siglec–sialoglycan checkpoints are a distinct axis of immune suppression, we would expect the combination of trastuzumab-hybrid AbLecs with CD47 blockade to further enhance ADCP of HER2^+^ cancer cells (Fig. 3i). Indeed, the combination of a CD47 antagonist antibody and T7 AbLec increased phagocytosis of cancer cells compared to T7 AbLec treatment alone in two of three donors tested (Fig. 3j and Extended Data Fig. 8j–l). Across all three donors, the combination of αCD47 and T7 AbLec outperformed the combination of αCD47 and trastuzumab (Fig. 3j and Extended Data Fig. 8j–l). The combination of αCD47 with T7 or T9 AbLec also enhanced PMN cytotoxicity over αCD47 and trastuzumab (Extended Data Fig. 5e). Our findings are especially encouraging in light of recent clinical results in gastric cancer showing that CD47 blockade significantly increased response rates when added to the standard of care treatment regimen, which includes trastuzumab^68^. Furthermore, we observed synergy of T7 AbLec and CD47 blockade using a concentration of αCD47 that was ineffective as a monotherapy (Fig. 3j, Extended Data Fig. 5e and Extended Data Fig. 8j–l). This is important because the high dose of the αCD47 antibody magrolimab required for single-agent efficacy is thought to be the major driver of its on-target, off-tumour toxicity, which ultimately resulted in the termination of multiple clinical programmes (for example, NCT05079230 (ref. ^69^), NCT06046482). Our data suggests that combinations of AbLecs with low-dose CD47 blockade could reduce off-tumour toxicity while preserving on-tumour efficacy. In sum, these results indicate that Siglec-7 is a distinct axis of inhibition in macrophages and that AbLecs have the potential to synergize with cancer immunotherapies in clinical development, extending their therapeutic benefits to more patients.

### AbLecs amplify pro-inflammatory signalling in macrophages

**Extended Data Fig. 9:**
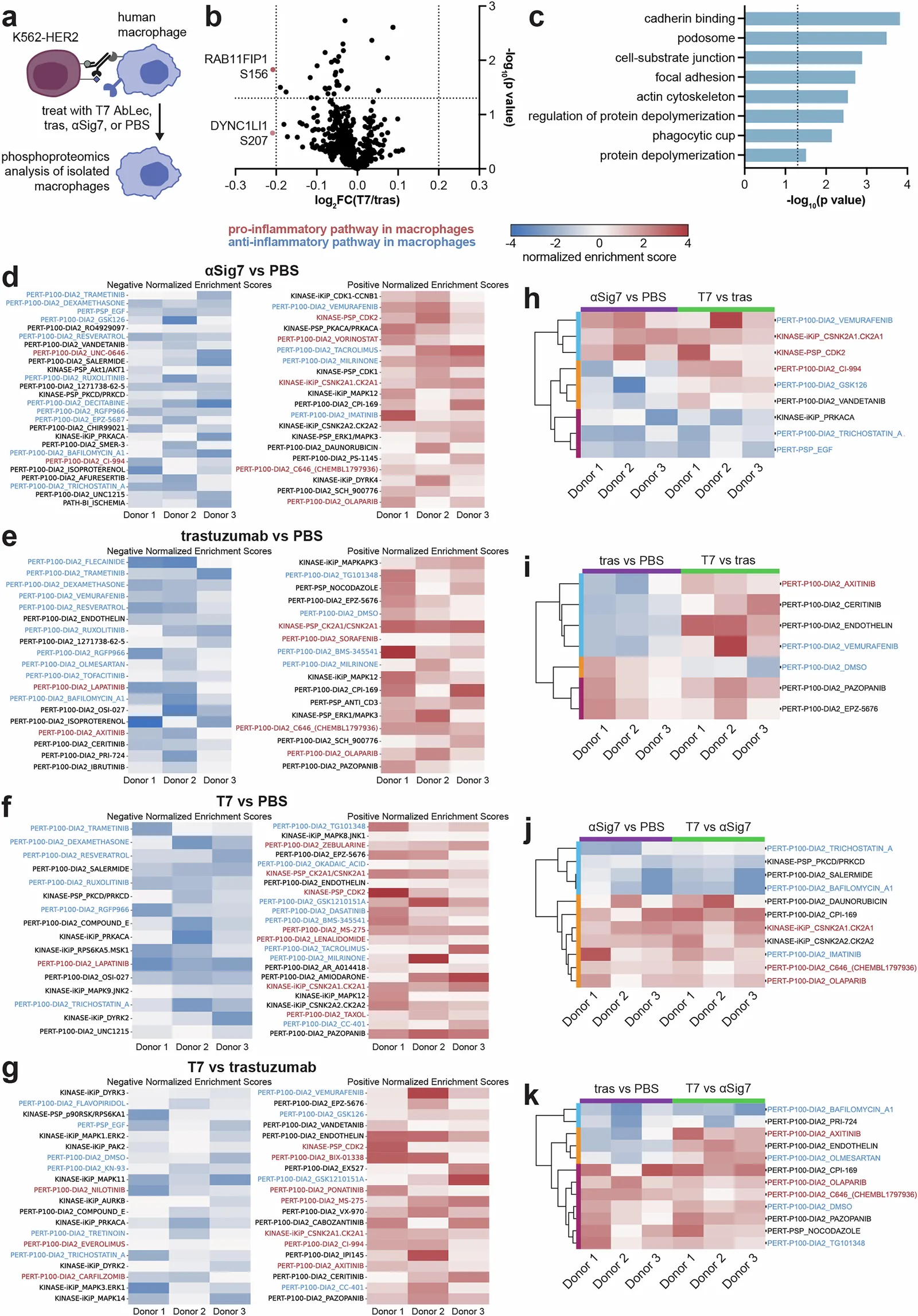
AbLecs amplify pro-inflammatory signaling in macrophages. (**a**) Primary human macrophages from n = 3 donors were co-cultured with K562-HER2 cells and treated with T7 AbLec, trastuzumab, Siglec-7 blocking antibody (αSig7), or vehicle for 6 hr. Following incubation, macrophages were isolated and lysed, lysates were trypsin digested, and phosphopeptides were enriched before TMT-labeling and LC-MS/MS analysis. (**b**) Volcano plots of significance vs. fold-change for phosphopeptides isolated from macrophage lysates following treatment with T7 AbLec or trastuzumab. Horizontal dashed line denotes the significance threshold (p = 0.05) and vertical dashed lines indicate fold-change cutoffs. Each datapoint represents the average fold-change from n = 2 biological replicates. (**c**) Gene Ontology (GO) term analysis of phosphosites enriched in macrophages treated with T7 AbLec vs trastuzumab. GO terms with statistically significant enrichment (p-value < 0.05, dashed line) are plotted. (**d-k**) PTM Signature Enrichment Analysis (PTM-SEA)^73^ analysis was used to identify pro-inflammatory and anti-inflammatory signatures activated in macrophages treated with Siglec-7 blocking antibody (αSig7), trastuzumab, or T7 across n = 3 donors. Pairwise comparisons of treatments (**d-g**) or pairwise comparisons of pairwise comparisons (**h-k**) are shown. Positive or negative normalized enrichment scores indicate whether the signature is upregulated or downregulated in the pairwise comparison. Signatures with names written in red have been shown to be pro-inflammatory in macrophages, while those with names written in blue have been shown to be anti-inflammatory in macrophages^76,145–200^. *P*-values were determined by unpaired, two-tailed student’s t test with a 5% false discovery rate; values are plotted in (**b**, **c**). Source data

Our discovery that AbLecs exclude Siglecs from the immunological synapse spurred us to investigate how AbLec treatment impacts immune cell signalling. To this end, we performed global phosphoproteomic analysis of primary human macrophages following co-culture with K562-HER2 cells and treatment with Siglec-7 blocking antibody (αSig7), trastuzumab, T7 AbLec or vehicle (Extended Data Fig. 9a). We initially compared phosphopeptide abundances in T7 AbLec-treated and trastuzumab-treated macrophages and found that AbLec treatment decreased phosphorylation of two proteins associated with the Rab11a complex that mediates endosomal recycling (Extended Data Fig. 9b). The Rab11 GTPase family governs a decision point at which endosomes can either mature to ultimately become lysosomes or recycle to the plasma membrane^70^. This is relevant because effective phagocytosis requires that phagosome maturation proceeds through successive fusion with early and late endosomes as well as lysosomes^71^. Our data suggest that AbLec treatment downregulates the activation of proteins involved in endosomal recycling, allowing more endosomes to mature and facilitating the increased phagocytosis that we observe in our ADCP assays. Indeed, previous work has shown that downregulation of Rab11a increases macrophage phagocytosis^72^. Gene Ontology term analysis provided further support for the idea that T7 AbLec treatment amplifies phagocytic signalling compared to trastuzumab. In AbLec-treated compared to trastuzumab-treated macrophages, we observed changes in the abundances of phosphopeptides involved in multiple cellular structures and processes related to phagocytosis, including cell–cell adhesion by cadherins and focal adhesions, the podosome, the phagocytic cup and rearrangement of the actin cytoskeleton (Extended Data Fig. 9c). These results provide insights into the cellular signalling underlying the ability of T7 AbLec to elicit enhanced ADCP compared to trastuzumab in our functional assays (for example, Fig. 2c).

For a more global understanding of the signalling pathways influenced by AbLec treatment, we leveraged post-translational modification signature enrichment analysis (PTM-SEA)^73^. PTM-SEA allowed us to identify kinase activities, signalling pathways and perturbations (for example, drug treatments) that match the dynamics of fold changes between treatment conditions for all identified phosphopeptides in our datasets (Extended Data Fig. 9d–g). We prioritized signatures that were consistently upregulated or downregulated for each comparison in assays with macrophages from three donors, to increase the likelihood that they represent the effects of treatment with αSig7, trastuzumab or T7 AbLec. For many of these signatures, we found evidence in the literature of their pro-inflammatory or anti-inflammatory effects in macrophages, which helped us better understand the overall impact of each treatment on macrophage phenotype (Extended Data Fig. 9d–g). We found the greatest number of pro-inflammatory signatures upregulated in T7 AbLec-treated macrophages compared to vehicle-treated controls (Extended Data Fig. 9d–f) and enrichment of multiple pro-inflammatory signatures in the T7 AbLec condition compared to trastuzumab (Extended Data Fig. 9g). Of particular interest was cyclin-dependent kinase 2 (CDK2) activity, which was upregulated in comparisons of T7 AbLec to vehicle and to trastuzumab (Extended Data Fig. 9f,g). CDK2 signalling supports macrophage activation^74–76^, and there is evidence that its kinase activity is negatively regulated by Siglec-E in murine immune cells^77^. CDK2 signalling was upregulated in comparisons of αSig7 to vehicle but not trastuzumab to vehicle, consistent with the idea that upregulation of CDK2 activity is a result of Siglec-7 blockade (Extended Data Fig. 9d,e). These data are consistent with our findings that T7 AbLec amplifies pro-inflammatory signalling by blocking Siglec-7 engagement.

Finally, we focused on signatures that were modulated in T7 AbLec-treated macrophages compared to trastuzumab-treated or αSig7-treated macrophages to understand signalling that contributes to the enhanced function of the AbLec over these controls observed in our ADCP assays. However, when we compared these signatures to those that were also present in our analyses of trastuzumab or αSig7-treated macrophages, we found some similarities but also differences (Extended Data Fig. 9h–k). Therefore, the signalling pathways activated by AbLec treatment may not be fully explained by the trastuzumab or Siglec blockade components alone and instead point to synergistic effects of their combination. Collectively, our phosphoproteomic analysis provides additional evidence that AbLec treatment enhances macrophage activation by amplifying pro-inflammatory signalling through a dual Siglec-dependent and FcγR-dependent mechanism of action.

### AbLecs can be designed to target a variety of cancers

**Fig. 4:**
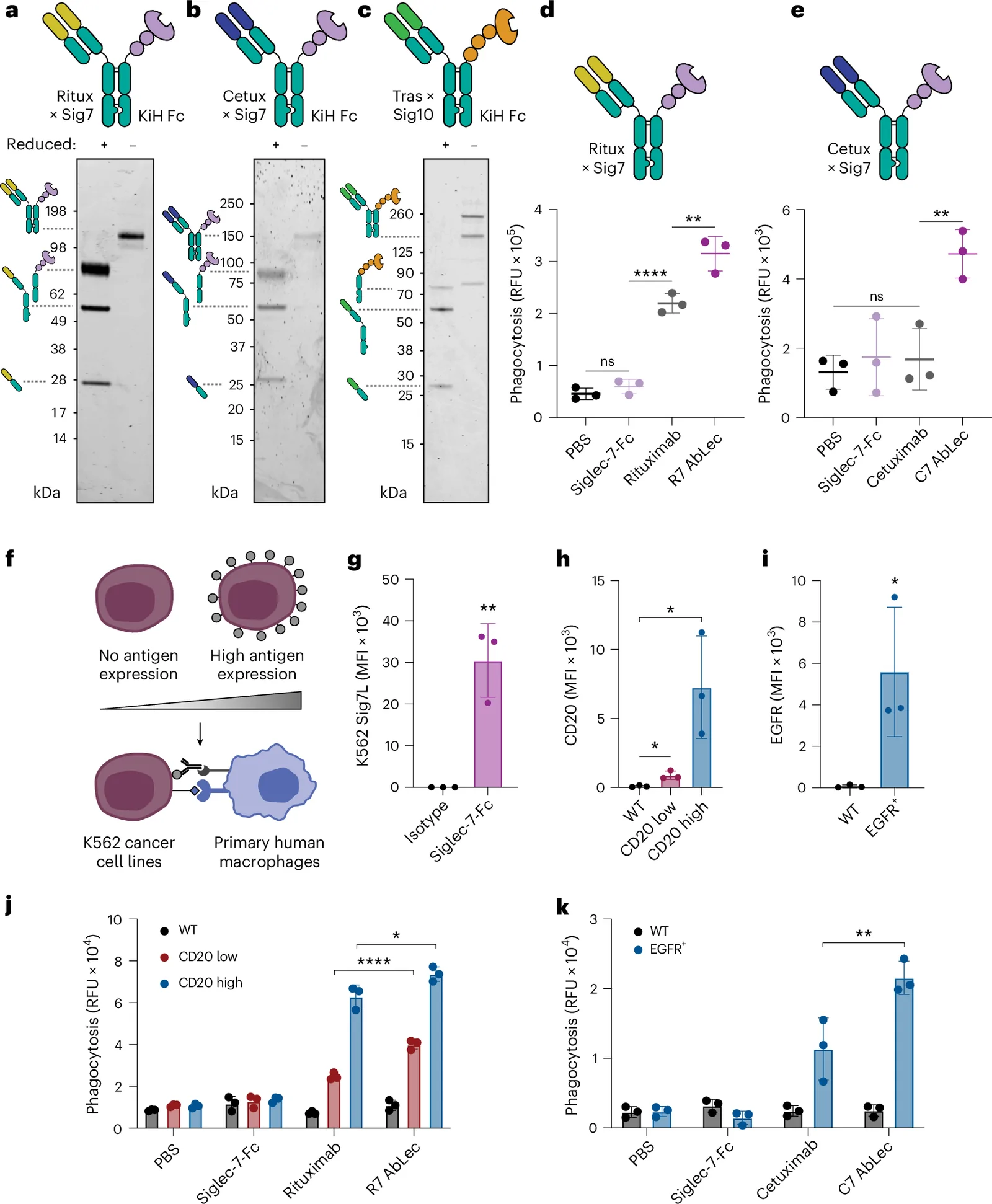
AbLecs can target multiple tumour-associated antigens at varying expression levels. **a**–**c**, Reducing (+) and non-reducing (−) SDS–PAGE analysis of purified rituximab (αCD20) × Siglec-7 (R7) AbLec (**a**), cetuximab (αEGFR) × Siglec-7 (C7) AbLec (**b**) and trastuzumab (αHER2) × Siglec-10 (T10) AbLec (**c**). Gels are representative of *n* = 3 replicates. **d**, Macrophage ADCP of Ramos cells treated with R7 AbLec, rituximab or Siglec-7-Fc. Data are mean ± s.d. of *n* = 3 biological replicates. **e**, Macrophage ADCP of K562-EGFR cells treated with C7 AbLec, cetuximab or Siglec-7-Fc. Data are mean ± s.d. of *n* = 3 biological replicates. **f**, We engineered K562 cell lines expressing high and low levels of tumour-associated antigens (CD20, EGFR). WT K562s are antigen-negative. These cell lines were used to interrogate AbLec efficacy in response to varying antigen densities. **g**, Sig7L expression measured by Siglec-7-Fc staining of K562 cells compared to hIgG1 isotype control. Data are mean ± s.d. of *n* = 3 biological replicates. **h**, CD20 expression on WT, CD20 low and CD20 high K562 cells measured by rituximab staining compared to hIgG1 isotype antibody by flow cytometry. Data are mean ± s.d. of *n* = 3 biological replicates. **i**, EGFR expression on K562 WT and K562-EGFR cells measured by cetuximab staining compared to hIgG1 isotype antibody by flow cytometry. Data are mean ± s.d. of *n* = 3 biological replicates. **j**, Macrophage ADCP of WT, CD20 low and CD20 high K562 cells treated with R7 AbLec, rituximab or Siglec-7-Fc. Data are mean ± s.d. of *n* = 3 biological replicates. **k**, Macrophage ADCP of K562 WT and K562-EGFR cells treated with C7 AbLec, cetuximab or Siglec-7-Fc. Data are mean ± s.d. of *n* = 3 biological replicates. *P* values were determined by Tukey-corrected one-way ANOVA (**d**, **e**, **j** and **k**) or two-tailed unpaired *t*-test (**g**–**i**); ns, not significant (*P* > 0.05); *P < 0.05; **P < 0.01; ***P < 0.001; ****P < 0.0001. Source data

As a feature of their modular chimeric architecture, AbLecs can be readily developed to target diverse cancers and glyco-immune checkpoints. To demonstrate this modularity, we constructed tumour-targeting AbLecs with components derived from rituximab × Siglec-7 (R7) and cetuximab × Siglec-7 (C7), designed to block Sig7L on CD20^+^ and EGFR^+^ cancer cells, respectively (Fig. 4a,b). In addition, we generated a trastuzumab × Siglec-10 AbLec intended to block engagement of the Siglec-10 receptor on HER2+ tumour cells, as Siglec-10 was recently shown to mediate immune evasion in breast, ovarian and colorectal cancers^15,34^ (Fig. 4c).

**Extended Data Fig. 10:**
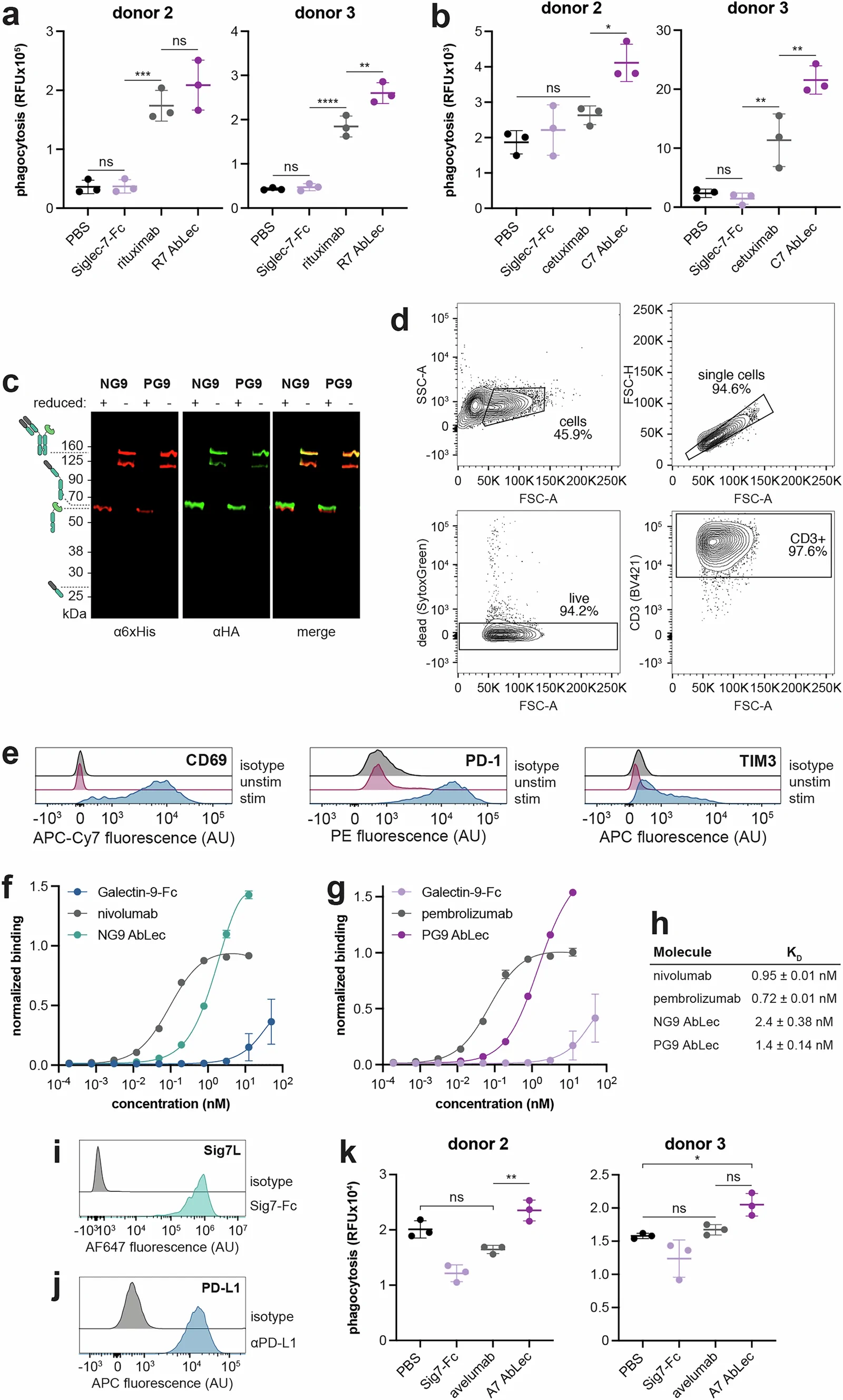
The chimeric AbLec architecture enables targeting of diverse cancers and multiple therapeutic mechanisms of action. (**a**) Macrophage ADCP of Ramos cells treated with R7 AbLec, rituximab, or Siglec-7-Fc. Data are mean ± s.d. of n = 3 biological replicates. (**b**) Macrophage ADCP of K562-EGFR cells treated with C7 AbLec, cetuximab, or Siglec-7-Fc. Data are mean ± s.d. of n = 3 biological replicates. (**c**) Western blot analysis of NG9 and PG9 AbLecs revealed that they are composed of Galectin-9-Fc chains (α6xHis) and nivolumab/pembrolizumab heavy + light chains (αHA), respectively. Blots are representative of n = 3 biological replicates. (**d**) Human T cells were defined as CD3+ by flow cytometry. (**e**) After 3 days of stimulation with αCD3/αCD28 antibodies, primary human CD3+ T cells upregulated CD69, PD-1, and TIM-3 by flow cytometry compared to unstimulated T cell and isotype controls. Histograms are representative of n = 3 biological replicates. (**f, g**) Binding of Galectin-9-Fc, NG9 AbLec and nivolumab (**f**), or PG9 AbLec and pembrolizumab (**g**) to activated T cells was quantified via flow cytometry. Data are mean ± s.d. of n = 3 biological replicates, normalized to staining with 50 nM nivolumab or pembrolizumab, respectively. (**h**) Apparent dissociation constants (K_D_) ± 95% confidence intervals for bivalent nivolumab, pembrolizumab, NG9 and PG9 AbLecs determined by fitting experimental data from (**f, g**) to one-site total binding curves. (**i, j**) MDA-MB-231 cells express Sig7L (**i**) and PD-L1 (**j**) by flow cytometry. Histograms are representative of n = 2 biological replicates. (**k**) Macrophage phagocytosis of SK-BR-3 cells treated with A7 AbLec, avelumab (αPD-L1), or Siglec-7-Fc. Data are mean ± s.d. of n = 3 biological replicates. *P*-values were determined by Tukey-corrected one-way ANOVA (**a**, **b**, **k**); ns p>0.05, *p < 0.05, **p < 0.01, ***p < 0.001, ****p<0.0001. Source data

Functional characterization of R7 and C7 AbLecs demonstrated the utility of the AbLec platform for combination tumour-targeting and glyco-immune checkpoint blockade in diverse tumour types. Across *n* = 3 donors, R7 C7 AbLec treatment significantly enhanced phagocytosis of CD20^+^ Ramos cells or K562-EGFR cells, respectively, compared to the parent antibody or Siglec-7-Fc alone (Fig. 4d,e and Extended Data Fig. 10a,b). Notably, and consistent with our findings with T7/9 AbLecs, R7 and C7 AbLecs selectively target antigen-expressing cells and spare antigen-negative cells (Fig. 4f–k). As a result of their antigen-dependent activity, which we observed in the context of three tumour-associated antigens, we expect AbLecs to exhibit safety profiles driven by the expression patterns of the targeted antigen, which are typically more favourable than those of systemic immune checkpoint blockade^42,44^. These data further show that AbLecs can be readily redesigned to target a variety of cancer antigens.

Low or heterogeneous expression of tumour-associated antigens on cancer cell surfaces has been associated with resistance to immunotherapies, including monoclonal antibodies^78,79^, immune checkpoint blockade^80^ and CAR-T cells^81,82^. We reasoned that the enhanced effector functions elicited by AbLecs might be able to overcome weak FcγR signalling potentiated by cancer cells with low antigen density. We created K562 lines expressing high and low levels of the CD20 antigen, while the expression of Sig7L was constant (Fig. 4f–h). In assays with primary human macrophages, we found that R7 AbLec treatment enhanced ADCP of tumour cells expressing very low levels of the targeted CD20 antigen compared to the parent monoclonal antibody, rituximab (Fig. 4f–h,j). Our results show that the improved efficacy of AbLecs compared to the parent monoclonal antibody is maintained in cancers with low antigen expression, a current area of unmet medical need.

### The AbLec platform is extensible to additional checkpoints and therapeutic mechanisms of action

**Fig. 5:**
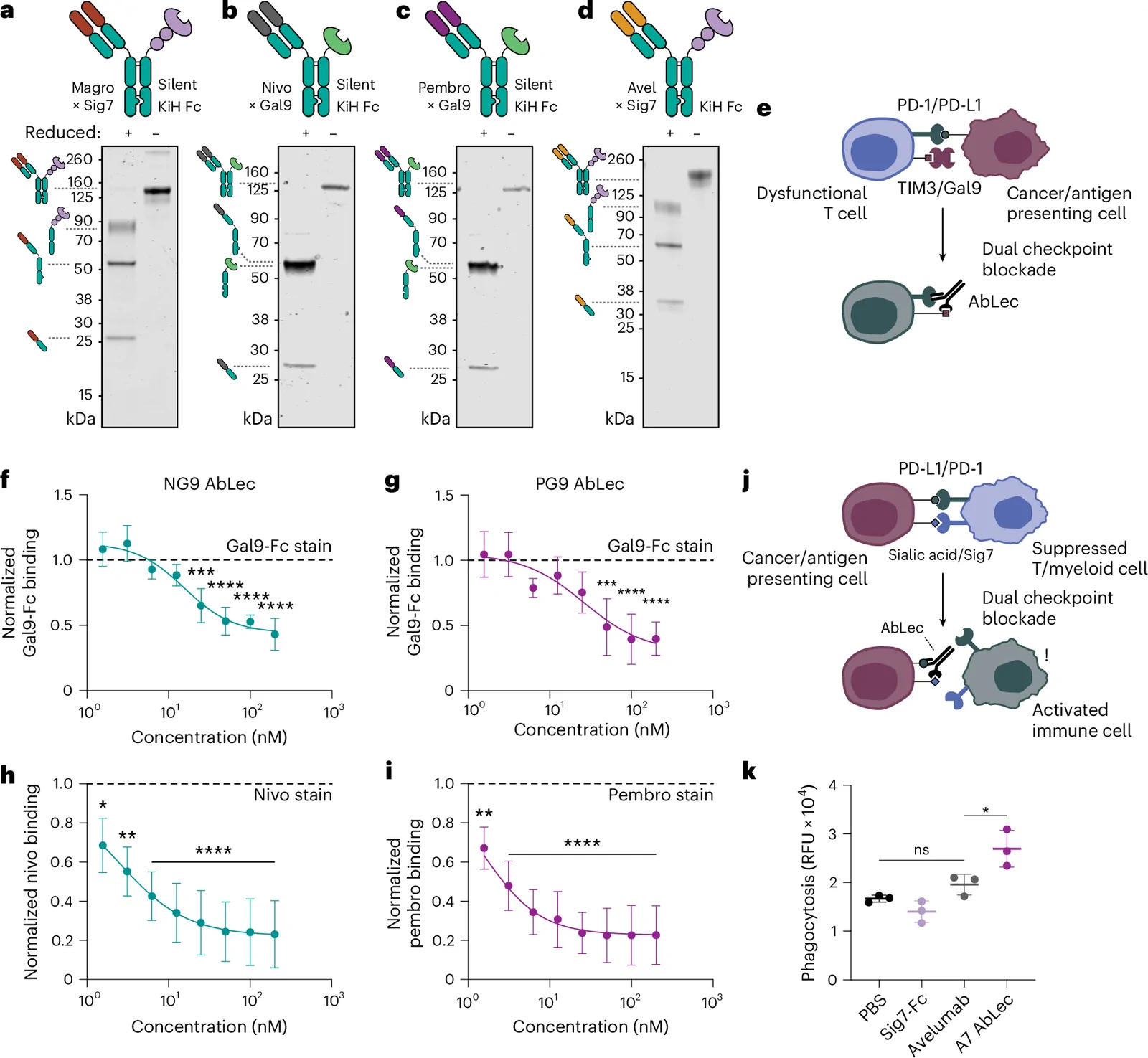
AbLecs for dual blockade of protein-based and glycan-based immune checkpoints. **a**–**d**, Reducing (+) and non-reducing (−) SDS–PAGE analysis of purified magrolimab (αCD47) × Siglec-7 (M7) AbLec (**a**), nivolumab (αPD-1) × galectin-9 (NG9) AbLec (**b**), pembrolizumab (αPD-1) × galectin-9 (PG9) AbLec (**c**) and avelumab (αPD-L1) × Siglec-7 (A7) AbLec (**d**). Gels are representative of *n* = 3 replicates. **e**, We developed dual checkpoint blockade AbLecs to simultaneously block the PD-1/PD-L1 and TIM-3/galectin-9 checkpoints on T cells. **f**,**g**, NG9 (**f**) or PG9 (**g**) AbLec treatment blocks galectin-9 receptor binding to activated T cells in competitive binding assays with dye-labelled galectin-9-Fc. Experimental data were fit using a four-parameter [inhibitor] vs response curve. Data are mean ± s.d. of *n* = 4 biological replicates, normalized to galectin-9-Fc staining without NG9 or PG9 AbLec. **h**,**i**, NG9 (**h**) or PG9 (**i**) AbLec treatment blocks dye-labelled nivolumab or pembrolizumab binding to activated T cells, respectively. Experimental data were fit using a four-parameter [inhibitor] vs response curve. Data are mean ± s.d. of *n* = 4 biological replicates, normalized to nivolumab or pembrolizumab staining without AbLec. **j**, We developed dual checkpoint blockade AbLecs to simultaneously block the PD-1/PD-L1 and Siglec-7 checkpoints that negatively regulate activation of both T cells and myeloid cells. **k**, Macrophage phagocytosis of MDA-MB-231 cells treated with A7 AbLec, avelumab (αPD-L1) or Siglec-7-Fc. Data are mean ± s.d. of *n* = 3 biological replicates. *P* values were determined by Tukey-corrected one-way ANOVA (**f**, **g**, **h**, **i** and **k**); ns, not significant (*P* > 0.05); *P < 0.05; **P < 0.01; ***P < 0.001; ****P < 0.0001. Source data

Numerous inhibitory receptors on immune cells regulate their activation. Often, multiple immune checkpoints are misregulated in tumours, contributing to disease progression and resistance to existing immunotherapies^64^. To address this challenge, we developed dual checkpoint blockade AbLecs designed to block engagement of both protein-based and glycan-based immune checkpoints (Fig. 5a–d). As a first example, we designed an AbLec for dual blockade of CD47 and Siglec-7 ligands on cancer cells by combining the αCD47 antibody magrolimab^69^ with a Siglec-7 decoy receptor domain (Fig. 5a). This molecule offers an alternative strategy for dual targeting of CD47 and Sig7L that could be combined with FDA-approved tumour-targeting antibodies. For example, addition of CD47 blockade to either trastuzumab or rituximab therapy improved efficacy in mouse and human cancers^67,68,83,84^. Our results and others’ have shown that a combination of CD47 and Siglec-7 blockade has the potential to even further amplify anticancer immune responses^23,25^ (Fig. 3i,j).

Cytotoxic T cells are among the most important effectors of antitumor immunity, as evidenced by the clinical success of checkpoint inhibitors (for example, PD-1/PD-L1 antagonists^85^) and T cell therapies (for example, TIL^86^ and CAR-T cell therapies^87^). However, inefficient CD8^+^ T cell priming, which requires CD4^+^ T cells^88,89^, and/or exhaustion have been implicated as key mechanisms of resistance^90,91^, necessitating new approaches to maintain T cell fitness. Glyco-immune checkpoints are known to restrain both CD4^+^ and CD8^+^ T cell function^19,21,46,92–96^, but it has been challenging to date to target their glycan ligands directly. As a proof-of-concept, we generated nivolumab × galectin-9 and pembrolizumab × galectin-9 AbLecs (NG9 and PG9 AbLecs, respectively) designed to simultaneously block the PD-1/PD-L1 and TIM-3/galectin-9 T cell checkpoints^46,92,95^ (Fig. 5a,b,e and Extended Data Fig. 10c). Nivolumab and pembrolizumab are PD-1 antagonist antibodies approved to treat diverse cancers^85,97–104^. The galectin-9 lectin is a ligand for the TIM-3 checkpoint receptor, and binding of galectin-9 to TIM-3 contributes to CD8^+^ T cell exhaustion and apoptosis^46,92,95^. Moreover, the PD-1 and TIM-3 T cell checkpoints synergize to suppress type 1 immune responses^46,92,95^, which generate T_H_1 CD4^+^ and CD8^+^ T cell populations that are critical for productive anticancer immunity. Both galectin-9 and TIM-3 antagonist antibodies are cancer immunotherapy approaches being investigated in clinical trials^39,105–107^, but these monospecific therapies cannot ensure effective blockade of both PD-1 and TIM-3 engagement on the same T cell. By contrast, both NG9 and PG9 AbLecs bound to activated human T cells at nanomolar concentrations (Extended Data Fig. 10d–h). Furthermore, NG9 and PG9 AbLecs exhibited distinct binding profiles compared to parent monoclonal antibody and galectin-9-Fc controls (Extended Data Fig. 10f,g), indicating contributions from both αPD-1 and galectin-9 domains to overall AbLec binding. NG9 and PG9 AbLecs blocked binding of fluorescently labelled galectin-9-Fc and their parent nivolumab and pembrolizumab antibodies, respectively, indicating that they occupy the same binding sites on activated human T cells (Fig. 5f–i). Therefore, NG9 and PG9 AbLecs can simultaneously block the PD-1/PD-L1 and TIM-3/galectin-9 immune checkpoints that restrain T cell activation.

Siglecs and sialoglycans are upregulated in patients who do not respond to PD-1 blockade^30^, indicating that this axis is a mechanism of resistance to drugs currently used to treat nearly half of all cancer patients^1^. To address this mechanism of resistance, we used the binding domain from the FDA-approved αPD-L1 antibody avelumab^108–110^ to engineer an avelumab × Siglec-7 AbLec for dual blockade of PD-L1 and Sig7L expressed on cancer or antigen-presenting cells (Fig. 5d). The A7 AbLec enhanced phagocytosis of MDA-MB-231 cells expressing PD-L1 and Sig7L compared to the avelumab antibody alone (Fig. 5j,k and Extended Data Fig. 10i–k). Taken together, dual blockade AbLecs target orthogonal immunosuppressive pathways in cancer^20,23,25,45^ and can be used alone or in combination with existing clinical-stage immunotherapies to amplify antitumor immune responses.

## Discussion

In this work, we establish AbLecs as a class of antibody chimeras targeting glyco-immune checkpoints for cancer immunotherapy. AbLecs combine antibodies against antigens of interest (for example, tumour-associated antigens, immune checkpoint receptors or ligands) with glycan-binding domains from immunomodulatory lectin receptors. We show that AbLecs bind to cell surfaces at therapeutically relevant concentrations and selectively block binding of the targeted lectin receptor. AbLecs enhanced antibody-mediated phagocytosis and cytotoxicity of cancer cells, including those with low expression of the targeted antigen, and reduced tumour burden in a humanized animal model. Synergy with blockade of established checkpoints demonstrated the potential for AbLecs to expand the subset of patients that benefit from cancer immunotherapy. Overall, AbLecs offer a modular molecular architecture for glycan blockade as an immunotherapeutic mechanism of action.

Importantly, this study could not fully explore the therapeutic possibilities enabled by the AbLec platform. There are many human cancers whose progression is influenced by Siglecs or galectins and that have targetable surface antigens for which additional AbLecs could be developed (for example, colorectal^28,34,96^, prostate^33,111,112^ and pancreatic^18,113^ cancers, glioblastoma^25,30^, melanoma^19,39^). Furthermore, it will be important to test AbLec efficacy in additional animal models of cancer and assess their in vivo synergy with approved immunotherapies (for example, PD-1/PD-L1 or CTLA-4 checkpoint inhibitors). Collectively, these future studies promise to identify the full spectrum of cancers that could benefit from AbLec treatment.

We found that T7 and T9 AbLecs were more effective Siglec antagonists than Siglec-blocking antibodies and as effective as sialoside degradation. Further investigation revealed that their potent antagonist activity is mediated by the exclusion of Siglec receptors from the immunological synapse. We suspect that this is a result of the chimeric AbLec architecture, which allows the Siglec decoy receptor arm to occupy Siglec ligands in close physical proximity to synapses formed by the trastuzumab antibody arm. Our data suggest that targeted lectin blockade mediated by AbLecs is at least as effective as systemic glyco-immune checkpoint blockade with the potential for reduced toxicity.

Notably, opportunities to influence organization of the immunological synapse for immunotherapy are not restricted to Siglecs or other glyco-immune checkpoints. Our work adds to a growing body of evidence that the recruitment of checkpoint receptors and/or tyrosine phosphatases to synapses restrains inflammatory signalling and immune cell activation^59,65,66,114^. By contrast, therapies such as AbLecs that mediate their exclusion reverse these effects^115^. These insights promise to inform the design of next-generation immune checkpoint inhibitors with improved efficacy.

Tumours substantially remodel their cell surface glycosylation. It is now clear that a key role for these remodelled glycans is to facilitate immune evasion and tumour progression by engaging multiple broad classes of lectin receptors^10–23,25,28–30,33,45,46,96,113,116–122^. Our chimeric AbLec molecules result in a gain-of-function: potent blockade of glyco-immune checkpoints that represent important mechanisms of immune suppression and therapeutic resistance in cancer. The AbLec approach is extensible to multiple cancers and mechanisms of action, with the potential to expand the efficacy of cancer immunotherapy.

## Methods

### Cell lines

All cell lines were purchased from the American Type Culture Collection (ATCC) unless otherwise noted. SK-BR-3, HCC-1954, K562 and Ramos cells were cultured in RPMI + 10% FBS without antibiotic selection. HER2^+^ B16D5 cells were a gift from the Weiner laboratory (Lombardi Cancer Center) and were cultured in DMEM with 10% FBS, 4 mM L-glutamine, 1 mM sodium pyruvate and without antibiotic selection. MDA-MB-231 cells were cultured in DMEM with 10% FBS, 4 mM L-glutamine, 1 mM sodium pyruvate and without antibiotic selection. Expi293F cells were a gift from the Kim lab (Stanford University) and were cultured according to the manufacturer’s protocol (Thermo Fisher Scientific). K562-EGFR cells were established by transducing K562 cells (ATCC) with pre-packaged lentiviral particles encoding EGFR (G&P Biosciences) according to the manufacturer’s protocol and selected for EGFR expression by culture in 1 µg ml^−1^ puromycin (InVivoGen). K562-CD20 cells were established by transducing K562 cells (ATCC) with pre-packaged lentiviral particles encoding CD20 (G&P Biosciences) according to the manufacturer’s protocol and sorted for high and low CD20-expressing cells using rituximab and a BV421-labelled anti-human secondary (Jackson ImmunoResearch). Lentiviral vector encoding *HER2/neu* was a gift from M.-C. Hung (Addgene plasmid, 16257). The stable K562-HER2 line was generated as previously described^123^, and HER2 expression was verified by flow cytometry. RAW264.7-Siglec-7–mGFP cells were established by transducing RAW264.7 cells (ATCC) with lentiviral particles encoding Siglec-7–mGFP. Lentivirus encoding Siglec-7–mGFP was produced in HEK293T cells transfected with pMD2.G (a gift from D. Trono; Addgene, 12259), pCMV-dR8.2 (Addgene, 8455) and a Siglec-7–mGFP fusion construct (Origene, RC206995L2) cloned into a lentiviral vector derived from pHRSIN-CSGW (Addgene, 113014) using lipofectamine LTX (Invitrogen, 15338–100). Media containing lentivirus was collected 72 h post transfection, filtered through a 0.45 μm filter and concentrated using LentiX (Takara Biosciences). After addition of the concentrated virus, cells were centrifuged at 2,000*g* for 45 min at 37 °C. RAW264.7-Siglec-7–mGFP cells were analysed a minimum of 60 h later and maintained for a maximum of 1 week. Cell lines were not independently authenticated aside from the identity provided by the ATCC. Cell lines were cultivated in a humidified incubator at 5% CO_2_ and 37 °C, except for Expi293F cells, which were cultivated at 8% CO_2_ and 37 °C, and tested negative for mycoplasma quarterly using a PCR-based assay.

### Plasmids and protein sequences

All antibody and AbLec plasmids were generated by Twist Bioscience and inserted into the pTwist CMV BetaGlobin vector using the XhoI and NheI cut sites, unless otherwise specified below. DNA and amino acid sequences are listed in Supplementary Table 1. Trastuzumab was expressed from a pCDNA3.1 vector described in our previous work^16^. The rituximab and cetuximab antibody variable sequences were generated by IDT and cloned into the variable regions of the VRC01 antibody plasmid vector (a generous gift from Peter Kim’s lab at Stanford) by using the In-Fusion cloning kit (Takara) according to the manufacturer’s protocol. The EndoFree Plasmid Maxi Kit (Qiagen) was used to prepare DNA for transfection.

### Protein expression and purification

Antibodies (trastuzumab, rituximab, cetuximab) and AbLecs were expressed by transient transfection in Expi293F cells (Thermo Fisher Scientific) according to the manufacturer’s protocol. For antibodies, a 1:1 heavy to light chain plasmid ratio by weight was used. The trastuzumab antibody’s heavy and light chains were co-expressed from a single plasmid. For AbLecs, a 2:1:1 ratio of lectin to heavy chain to light chain was used. After 7 days of expression, proteins were collected from the supernatant by pelleting cells at 500*g* for 5 min, followed by clarification with a spin at 4,000*g* for 40 min, and 0.2 μm filtration. Antibodies were purified by manual gravity column (Bio-Rad) using protein A agarose (Fisher Scientific). In brief, the clarified supernatant was passed over the column twice; bound protein was treated with sialidase on the resin with 0.25–0.5 column volumes of 100 nM *Vibrio cholerae* sialidase in PBS for 0.5–2 h at room temperature (21 °C), washed with 20 column volumes of PBS and eluted twice with 5 ml 100 mM glycine buffer pH 2.8 into tubes pre-equilibrated with 150 μl of 1 M Tris pH 8. AbLecs were purified by manual gravity column using nickel-NTA agarose resin (Qiagen). In brief, AbLec supernatant was incubated with PBS-equilibrated resin for ~1 h at 4 °C. Resin and supernatant were then loaded onto a chromatography column, and bound protein was treated with sialidase on the resin with 0.25–0.5 column volumes of 100 nM *V.* *cholerae* sialidase in PBS for 0.5–2 h at room temperature, then washed with 20 column volumes of PBS + 20 mM imidazole and eluted twice with five column volumes of PBS + 250 mM imidazole. Antibodies and AbLecs were buffer-exchanged into PBS using PD-10 desalting columns (GE). Purified avelumab monoclonal antibody was purchased from Selleck Chemicals, and purified anti-GD2, nivolumab and pembrolizumab antibodies were purchased from BioXCell.

### AbLec gel characterization

For SDS–PAGE gels, 1 μg of protein with SDS loading dye (non-reducing conditions), or SDS dye + 1 M β-mercaptoethanol, heated at 95 °C for 5 min (reducing conditions), was loaded onto a Criterion XT 4–12% Bis-Tris Protein Gel, 18-well (Bio-Rad) and run with XT-MES buffer at 180 V for 40–60 min. Total protein content was visualized using Aquastain (Bulldog Bio) with staining for 10 min followed by a 10 min destain in water. For western blots, 0.2 μg of protein was loaded onto an SDS–PAGE gel and run as described above and then transferred to a nitrocellulose membrane using the Trans-Blot Turbo RTA Midi Nitrocellulose Transfer Kit (Bio-Rad) at 25 V for 14 min. The membrane was blocked in FACS buffer for 0.5–1 h at room temperature, then stained with anti-HA Tag Polyclonal Antibody (clone SG77, Thermo Fisher Scientific; 1:500–1:5,000) and anti-6×His antibody (clone J099B12, BioLegend; 1:2,000–1:5,000) for 1 h in FACS buffer with shaking at room temperature. The membrane was washed three times in PBST (PBS + 0.1% Tween), followed by staining with secondary antibodies IRDye 800CW Goat anti-Mouse or Goat anti-Rabbit and IRDye 680RD Goat anti-Rabbit or Goat anti-Mouse (all secondaries; LI-COR; 1:15,000) in PBST for 0.5–1 h with shaking at room temperature, followed by three washes in PBST and one wash with PBS before imaging. All gels and blots were imaged on an Odyssey CLx Imaging System (LI-COR), and images were analysed using Image Studio Lite (v.5.2.5).

### AbLec MS characterization

A total of 5 μg of each AbLec was digested with either trypsin or chymotrypsin for proteomic analysis. Samples were incubated for 30 min at 55 °C with 5 mM Tris(2-carboxyethyl)fosfine (TCEP), followed by a 30 min incubation at room temperature in the dark with 20 mM 2-chloroacetamide, and then quenched for 15 min at room temperature using an additional 2.5 mM TCEP. A final concentration of 1% sodium deoxycholate was added, followed by trypsin or chymotrypsin (Promega) at a 1:20 w/w ratio, and digestions proceeded at 37 °C overnight. The following morning, a 2% formic acid v/v was added twice to precipitate the SDC and quench the digestion. Samples were desalted over a reversed-phase solid-phase extraction (Strata-X) cartridge (Phenomenex), following the vendor-supplied protocol. Samples were dried using vacuum centrifugation and then resuspended in 0.1% formic acid in water at a concentration of 0.5 µg µl^−1^ for MS analysis.

Approximately 1 µg of peptide was injected per analysis, wherein peptides were separated over a 25 cm Aurora Series Gen2 reverse-phase liquid chromatography column (75 μm inner diameter packed with 1.7 μm FSC C18 particles; Ion Opticks). The mobile phases (A, water with 0.1% formic acid; B, acetonitrile with 0.1% formic acid) were driven and controlled by a Vanquish Neo UHPLC system (Thermo Fisher Scientific). Gradient elution was performed at 300 nl min^−1^. Mobile phase B was held at 0% and set to 2.2% at 0.1 min, followed by an increase to 25% at 85 min, a ramp to 99% B at 85 min and washed using consecutive iterations of 99% to 2% B for 5 min, for a total analysis time of 90 min. Flow was then ramped back to 0% B to re-equilibrate the column at 0% B. Eluted peptides were analysed on an Orbitrap Ascend Tribrid MS system^124^ (Thermo Fisher Scientific). Precursors were ionized using a Nanospray Flex ionization source (Thermo Fisher Scientific) held at +2.0 kV compared to ground. The inlet capillary temperature was held at 275 °C. Survey scans of peptide precursors were collected in the Orbitrap from 375–1600 *m*/*z* with an MS1 AGC target of 10% (400,000 charges), a maximum injection time of 251 ms and a resolution of 120,000 at 200 *m*/*z*. Monoisotopic precursor selection was enabled for peptide isotopic distributions, precursors of *z* = 2–6 were selected for data-dependent tandem mass spectrometry (MS/MS) scans for 3 s of cycle time and dynamic exclusion was set to 30 s with a ±10 ppm window set around the precursor monoisotope. An isolation window of 0.7 *m*/*z* was used to select precursor ions with the quadrupole. MS/MS scans were collected using higher-energy collisional dissociation at 30 normalized collision energy with an AGC target of 200% (100,000 charges) and a maximum injection time of 59 ms. Mass analysis was performed in the Orbitrap with a resolution of 30,000 at 200 *m*/*z* and scan range set to auto calculation.

Raw data were processed using Byonic (v.5.5.2). Oxidation of methionine (+15.994915) was set as a common^125^ variable modification, protein amino-terminal acetylation (+42.010565) and asparagine deamination (+0.984016) were specified as rare variable modifications and carbamidomethylation of cysteine (+57.021464) was set as a fixed modification. Up to three common and two rare modifications were permitted. A precursor ion search tolerance of 10 ppm and a product ion mass tolerance of 20 ppm were used for searches, and three missed cleavages were allowed for full trypsin specificity (carboxy-terminal of RK) and chymotrypsin (C-terminal of FLWY). Peptide spectral matches were made against custom FASTA sequence files that contained appropriate combinations of Siglec-7 and Siglec-9 holes, and antibody knobs and light chains. Peptides were filtered to a 1% false discovery rate, and a 1% protein false discovery rate was applied according to the target-decoy method. All peptide identifications were manually inspected, and sequences coverages were calculated only from validated peptide identifications. Sequence coverage percentages are derived from the proportion of amino acids explained by peptide identifications relative to the total number of amino acids.

### AbLec thermal stability measurements

T7/9 AbLecs, trastuzumab and Siglec-7/9-Fcs were normalized to equal concentrations between 0.1 and 1 mg ml^−1^ in PBS.

For SYPRO Orange assays, 20 µl of protein was mixed with SYPRO Orange Dye at a final dye concentration of 5× (diluted from 5,000× stock) in a PCR plate and sealed with an optically clear adhesive cover. Reactions were run and imaged using a CFX384 real-time PCR system (Bio-Rad). Following an initial 2 min incubation at 25 °C, the temperature was increased in 0.5 °C increments to 95 °C. Fluorescence was imaged following a 1 min incubation–equilibration period at each temperature. Melting curves were plotted as the first derivative of SYPRO Orange fluorescence with respect to temperature, and melting temperatures were determined as the lowest temperature at which a local maximum in this derivative occurred.

Nano differential scanning fluorimetry (nanoDSF) assays were performed using a Prometheus Panta instrument (NanoTemper Technologies). Protein samples were loaded into Prometheus Standard Capillaries (NanoTemper Technologies), and a nanoDSF thermal gradient was run from 25 °C to 95 °C with a step size of 0.5 °C min^−1^. Melting curves were plotted as the first derivative of the ratio of fluorescence emission at 350 nm/330 nm with respect to temperature. Melting temperatures were determined as the lowest temperature at which a local minimum or maximum in this derivative occurred.

### Lectin flow cytometry

Cells were pelleted by centrifugation, washed once with PBS and resuspended in FACS buffer (PBS with 0.5% BSA). Siglec-Fc or human IgG1 isotype Fc (R&D Systems) precomplexes with Alexa Fluor 647 (AF647)-labelled donkey anti-human IgG antibody (Jackson ImmunoResearch) were prepared at equimolar concentrations (7.5 nM) in FACS buffer. Biotin-labelled MALII lectin (Vector Labs, 10 μg ml^−1^) was precomplexed with AF647-Streptavidin (Thermo Fisher Scientific, 1 μg ml^−1^) in FACS buffer. Staining was performed for 30 min on ice at 1 × 10^6^ cells per ml. Cells were subsequently washed twice with ice-cold FACS buffer, resuspended in FACS buffer containing 100 nM SytoxGreen or 1 μM SytoxBlue (Thermo Fisher Scientific) and analysed by flow cytometry (BD LSR II or BD Symphony).

### Antibody flow cytometry

Cells were pelleted by centrifugation, washed once with PBS and resuspended in FACS buffer, as above. To quantify HER2, GD2, CD20 or EGFR expression, cells were stained with 7.5 nM antibody (trastuzumab, anti-GD2, rituximab or cetuximab, respectively) or a human IgG1 isotype control in FACS buffer for 30 min on ice at 1 × 10^6^ cells per ml. Cells were subsequently washed twice with ice-cold FACS buffer, resuspended in FACS buffer with 7.5 nM AF647-labelled donkey anti-human IgG secondary antibody (Jackson ImmunoResearch) and stained for 30 min on ice. Following incubation with the secondary antibody, cells were washed twice with ice-cold FACS buffer, resuspended in FACS buffer containing 100 nM SytoxGreen or 1 μM SytoxBlue (Thermo Fisher Scientific) and analysed by flow cytometry (BD LSR II or BD Symphony). For all other flow experiments, cells were stained with 10 μg ml^−1^ dye-labelled antibodies or the appropriate isotype controls in FACS buffer for 30 min at 4 °C. All antibody clones used are listed in Supplementary Table 2. Following incubation, cells were washed twice with ice-cold FACS buffer, resuspended in FACS buffer containing 100 nM SytoxGreen or 1 μM SytoxBlue (Thermo Fisher Scientific) and analysed by flow cytometry (BD LSR II or BD Symphony).

### AbLec and antibody binding isotherms

K562-HER2, SK-BR-3 or activated primary human CD3^+^ T cells were isolated from the cell culture supernatant or by dissociation with TrypLE (Gibco), respectively, washed with 1× PBS and resuspended in FACS buffer. A total of 60,000 cells were then distributed into wells of a 96-well V-bottom plate (Corning). Various concentrations of trastuzumab, nivolumab, pembrolizumab, Siglec-Fcs (R&D Systems), Galectin-9-Fc (Sino Biological) or AbLecs were added to the cells in equal volumes and incubated with cells for 1 h at 4 °C with periodic pipet mixing. Cells were washed three times in FACS buffer (PBS + 0.5% BSA), pelleting by centrifugation at 300*g* for 5 min at 4 °C between washes. Cells were resuspended in 4 μg ml^−1^ AF647-labelled donkey anti-human secondary antibody (Jackson ImmunoResearch) in FACS buffer for 30 min at 4 °C. Cells were further washed twice and resuspended in FACS buffer, and fluorescence was analysed by flow cytometry (BD LSR II or BD Symphony). Gating was performed using FlowJo (v.10.0) software (Tree Star) to eliminate debris and isolate single cells. Mean fluorescence intensity (MFI) of the cell populations was normalized to the MFI of cells stained with 50 nM trastuzumab from each experimental replicate. MFI values were fit to a one-site total binding curve using GraphPad Prism (v.10.4), which calculated the apparent *K*_D_ values as the antibody concentration needed to achieve a half-maximum binding.

### Computational modelling of AbLec binding and Siglec receptor competition

*MVsim*^55^, a multivalent binding simulation application (v.1.1 with MATLAB 2021a), was used to model the binding of trastuzumab, T7 AbLec, T7A mutant AbLec and Siglec-7-Fc to K562-HER2 cells. *MVsim* models the effects of molecular valency, topology, affinity and kinetics on the binding dynamics of multivalent and multispecific macromolecular receptor–ligand systems. Here, *MVsim* was used to create a modelling routine that simulated, through the microstate binding model^55^, the interaction of antibodies and antibody-like chimeras to surfaces populated with specific targeted ligands/antigens. The designed sequences and structures of the biomolecules were used to parameterize the model with the binding valencies. Here, the trastuzumab, T7A AbLec and Siglec-7-Fc reference biomolecules, as well as the T7 AbLec, were modelled as topologically related, Fc domain-driven, immunoglobulin dimers, each with a valency of two. The model was further parameterized with monovalent binding affinities that were experimentally estimated here (as described in the previous section, ‘AbLec and antibody binding isotherms’) or informed by the supporting literature. Affinities were parameterized as follows: trastuzumab monovalent *K*_D_ = 11.2 nM and Siglec-7-Fc monovalent *K*_D_ = 200 µM^126,127^.

The parameterized model was then used to fit the flow cytometry-based experimental binding data for the avidities (*K*_D,eff_) and effective concentration ([*C*_eff_]) parameters that derive from the spatial, nearest-neighbour proximity of pairs of targeted cell surface ligands or antigens. This was done by using a 2:3 HER2:Siglec-7 ratio and fitting for a global maximum MFI across all binding curves to constrain experimental binding curves that did not reach saturation (for example, Siglec-7-Fc). All simulations also included a 270 s dissociation step after equilibrium to mimic the delays arising from sample preparation and data collection by flow cytometry. The parameterized model was then further used to simulate competition experiments between Siglec-7-Fc and T7 AbLec on K562-HER2 cells. The setup of the competitive binding model is described in Supplementary Fig. 1d,e, and the ligand concentrations (0–100 nM AbLec and 200 nM Siglec-7-Fc) mirrored the experimental conditions to which the model was compared.

### Expression and dye labelling of lectin-Fc and antibody reagents

Siglec-Fc decoy receptors were expressed in Expi293F cells that stably express the human formylglycine-generating enzyme for aldehyde tagging. Siglec-Fc constructs were expressed according to the Expi293F manufacturer’s protocol (Thermo Fisher Scientific) and purified by manual gravity column (Bio-Rad) using protein A agarose (Fisher Scientific) as described for antibodies above, with the modification that transfected cells, supernatant and purified Siglec-Fcs were stored in the dark. Siglecs were then buffer-exchanged into acidic buffer, concentrated and conjugated with HIPS-azide as we previously described^16^. Siglec-Fc-azide was then buffer-exchanged into PBS. Then, 100× molar equivalents of DBCO-AF647 (Click Chemistry Tools, 1302-1) in dimethylsulfoxide (DMSO) were added, and the reaction was mixed at 500 rpm in the dark for 2 h at room temperature. Siglecs were buffer-exchanged into PBS by 6× centrifugation on Amicon columns (30 kDa MWCO), and AF647 addition was confirmed by using a NanoDrop spectrophotometer at 650 nm for the AF647 dye (extinction coefficient, 239,000) and at 280 nm for the protein (using extinction coefficients calculated for each protein by ExPASy)^128^.

Dye-labelled galectin-9-Fc (Sino Biological), nivolumab (BioXCell) and pembrolizumab (BioXCell) were generated by site-specific conjugation. In brief, oYo-Link Azide reagent (Alpha Thera) was mixed with 1.5 molar equivalents of DBCO-Alexa Fluor 647 (Vector Laboratories) and incubated in the dark at 4 °C with end-over-end rotation. The next day, 100 μg galectin-9-Fc or antibody was added to clear PCR tubes (<150 μl total reaction volume) or 1.5 ml microcentrifuge tubes (150 μl to 1 ml total reaction volume). Reaction tubes were incubated on their sides on ice 1–4 inches (~2.5–10 cm) below a 365–385 nM UV lamp for 2 h. AF647-labelled protein was purified from free dye/oYo-Link using 40 kDa molecular weight cutoff Zeba spin columns (Thermo Fisher Scientific) or 100 kDa molecular weight cutoff Amicon columns (Sigma-Aldrich). AF647 addition was confirmed by using a NanoDrop spectrophotometer at 650 nm for the AF647 dye (extinction coefficient 239,000) and at 280 nm for the protein (using extinction coefficients calculated for each protein by ExPASy)^128^.

### Competitive binding assays

K562-HER2 or activated primary human T cells were isolated from the cell culture supernatant, washed with 1× PBS and resuspended in FACS buffer. Cells were aliquoted for sialidase treatment with 100 nM *V.* *cholerae* sialidase at 37 °C for 30 min in FACS buffer. The *V.* *cholerae* sialidase cleaves all linkages (α2,3; α2,6; and α2,8) of terminal sialic acid, and thus can effectively degrade Siglec ligands^12,16^. Expression and purification of *V.* *cholerae* sialidase was performed as previously reported^12^. A total of 60,000 untreated or sialidase-treated cells were then distributed into wells of a 96-well V-bottom plate (Corning). Various concentrations of antibodies or AbLecs were combined with 200 nM Siglec-7/9-Fc-AF647, 0.3 μg ml^−1^ MEM-59-AF647, 25 nM Galectin-9-Fc-AF647, 0.1 nM nivolumab-AF647 or 0.1 nM pembrolizumab-AF647, added to the cells in equal volumes and incubated with cells for 2–3 h at 4 °C with periodic pipet mixing. Cells were washed three times and resuspended in FACS buffer, pelleting by centrifugation at 300*g* for 5 min at 4 °C between washes, and fluorescence was analysed by flow cytometry (BD LSR II or BD Symphony). Gating was performed using FlowJo (v.10.0) software (Tree Star) to eliminate debris and isolate single cells. The MFI of the cell populations was background-subtracted and normalized to the MFI of cells stained with 200 nM Siglec-7/9-Fc-AF647, 0.3 μg ml^−1^ MEM-59-AF647, 25 nM Galectin-9-Fc-AF647, 0.1 nM nivolumab-AF647 or 0.1 nM pembrolizumab-AF647 without antibody or AbLecs from each experimental replicate.

### Cell growth and toxicity assays

SK-BR-3 and K562-HER2 cells were isolated from the cell culture supernatant or lifted with trypsin (Gibco), washed with 8 ml culture media, pelleted by centrifugation at 300*g* and resuspended in phenol-red-free culture medium containing 50 nM Sytox green cell dead stain (Thermo Fisher Scientific) for K562-HER2 cells or 5 nM Sytox red dead cell stain (Thermo Fisher Scientific) for SK-BR-3 cells to measure cytotoxicity. Cells were plated onto a flat-bottomed 96-well plate (10,000 cells per well, 95 μl); then, 5 μl of AbLec, Siglec or antibody in PBS was added and mixed, followed by centrifugation at 30*g* for 1 min. Images were acquired using an IncuCyte S3 Live-Cell Analysis System (Sartorius) every 2 h for 3 days. SK-BR-3 cells were analysed by phase with segmentation adjustment = 1 and a minimum area of 200 μm^2^; cell death was quantified by red fluorescence using a threshold of 0.3 red calibrated unit (RCU), with an edge sensitivity of −50 and areas between 50 and 1,000 μm. K562-HER2 phase segmentation adjustment was 0.2, with no hole-fill and a minimum area of 60 μm. Cell death was quantified by green fluorescence using a threshold of 2 RCU, with an edge sensitivity of −45 and areas between 50 and 800 μm with eccentricity and integrated intensities less than 0.95 and 40,000, respectively.

### Isolation and differentiation of human macrophages

LRS chambers were obtained from anonymous healthy donors through the Stanford Blood Center. PBMCs were isolated using Ficoll-Paque (Sigma-Aldrich) density gradient centrifugation. Isolated PBMCs were extracted from the PBS/Ficoll interface, washed once with PBS and resuspended in serum-free RPMI. Monocytes were isolated by plating ~1 × 10^8^ PBMCs in a T75 flask containing serum-free RPMI for 1–2 h, followed by three washes with PBS +Ca +Mg to remove non-adherent cells. The media was then replaced with IMDM with 10% Human AB Serum (Gemini) and incubated for 7–9 days to induce macrophage differentiation before use in phagocytosis or flow cytometry experiments.

### Phagocytosis assays

Macrophages were washed with PBS and lifted by 30 min incubation at 37 °C with 10 ml TrypLE (Thermo Fisher Scientific). Macrophages were pelleted by centrifugation at 300*g* for 5 min and resuspended in IncuCyte medium (phenol-red-free RPMI + 10% HI FBS). Macrophages (10,000 cells in 100 μl) were added to a 96-well flat-bottom plate (Corning) and incubated in a humidified incubator for 1 h at 5% CO_2_ and 37 °C. Meanwhile, target cells were washed once with PBS, then treated with 1:80,000 diluted pHrodo red succinimidyl ester dye (Thermo Fisher Scientific) in PBS at 37 °C for 30 min, washed once with PBS and resuspended in IncuCyte medium. Finally, 10 μl of 20× antibody or AbLec stocks in PBS were added to the macrophages, followed by the addition of pHrodo red-stained target cells (20,000 cells in 90 μl). Antibodies, Siglec-Fcs and AbLecs were added at 25 nM, Siglec-7 and Siglec-9 blocking antibodies (1E8 and mAbA clones, respectively^20^; Creative Biolabs) were used at 12.5–25 μg ml^−1^, *V.* *cholerae* sialidase was used at 100 nM and human IgG1 isotype antibody (BioXCell), rat IgG2a isotype antibody (BioXCell) and CD47 blocking antibody (clone B6H12, BioXCell)^23^ were added at 10 μg ml^−1^ unless otherwise noted. For FcγR blocking experiments, macrophages were pre-treated during the 1 h incubation in the 96-well assay plate with human IgG1 isotype antibody (BioXCell), which binds and occupies human FcγRs, or rat IgG2a isotype antibody (BioXCell) as a control with minimal binding to human FcγRs^129^. For Siglec-7/9 blocking experiments, macrophages were pre-treated during the 1 h incubation in the 96-well assay plate with Siglec-7 and Siglec-9 blocking antibodies (1E8 and mAbA clones, respectively^20^; Creative Biolabs). Cells were plated by gentle centrifugation (50*g*, 2 min). Two images per well were acquired using an IncuCyte S3 Live-Cell Analysis System (Sartorius) at 1 h intervals until the maximum signal was reached. The quantification of pHrodo red fluorescence was empirically optimized for phagocytosis of each cell line based on their background fluorescence and size. K562 and MDA-MB-231 cells were analysed with a threshold of 0.8, an edge sensitivity of −70 and the area was gated between 100 and 2,000 μm^2^ with integrated intensities between 300 and 2,000 RCU × μm^2^ per image. HCC-1954 were analysed with a threshold of 1.5, an edge sensitivity of −45 and an area between 30 and 2,000 μm^2^. SK-BR-3 analysis had a threshold of 1, an edge sensitivity of −55, a minimum integrated intensity of 60 and a maximum area and eccentricity of 3,000 and 0.96, respectively. Ramos and Raji analysis used a threshold of 1.5, an edge sensitivity of −45 and areas between 100 and 2,000 μm^2^. The total red object integrated intensity (RCU × µm^2^ per image) was quantified for each image, and the maximum total red object integrated intensity value is reported for each treatment condition.

### Acquisition of IncuCyte images

For phagocytosis and cell growth assays, images were obtained over time using an Incucyte S3 Live-Cell Analysis System (Essen BioScience) within a Thermo Fisher Scientific tissue culture incubator maintained at 37 °C with 5% CO_2_. Data were acquired from a ×10 objective lens in phase contrast, a green fluorescence channel (excitation, 460 ± 20; emission, 524 ± 20; acquisition time, 300 ms) and from a red fluorescence channel (excitation, 585 ± 20; emission, 665 ± 40; acquisition time, 400 ms). Two images per well were acquired at intervals.

### Isolation of human NK cells

PBMCs were isolated from LRS chambers as described above, and cryostocks were prepared at 2–4 × 10^7^ cells in 90% heat-inactivated FBS + 10% DMSO and stored in liquid nitrogen vapour until use. The day before use, stocks were thawed and NK cells were isolated using an EasySep NK isolation kit (StemCell Technologies). Isolated NK cells were cultured with 0.5 µg ml^−1^ recombinant IL-2 (BioLegend) in RPMI + 10% heat-inactivated FBS for 24 h before use in NK cell cytotoxicity or flow cytometry experiments.

### NK cell killing assays

Target cells were lifted with TrypLE (Thermo Fisher Scientific) and stained with CellTracker Deep Red dye (Thermo Fisher Scientific) according to the manufacturer’s protocol. NK cells and target cells were mixed at an effector to target ratio of 4:1 (SK-BR-3 cells) or 2:1 (K562 cells), and Sytox Green (Thermo Fisher Scientific) was added at 100 nM. Antibodies, Siglec decoy receptors and AbLecs were added at 25 nM, Siglec-7 blocking antibody (clone 1E8, Creative BioLabs)^20^ was used at 12.5–25 μg ml^−1^, CD16-blocking antibody (clone 3G8, BioLegend) was added at 10 μg ml^−1^ and *V.* *cholerae* sialidase was used at 100 nM unless otherwise noted. For FcγR and Siglec-7 blocking experiments, NK cells were pre-treated with CD16-blocking antibody (clone 3G8, BioLegend) or Siglec-7 blocking antibody (clone 1E8, Creative BioLabs)^20^, respectively, before the addition of target cells and other treatments. After a 5–6 h incubation at 5% CO_2_ and 37 °C, cell death was analysed by flow cytometry by selecting the CellTracker Deep Red^+^ cells and quantifying the per cent dead as Sytox Green ± total CellTracker Deep Red^+^ cells. NK cytotoxicity index was then calculated as the ratio of dead to alive cells in the assay: % dead / (100 − % dead).

### Isolation of human PMNs

PMNs were isolated from the peripheral blood of healthy donors by density gradient centrifugation using Polymorphprep (Progen), as previously described^130^. Isolated PMNs were >82% pure by flow cytometry.

### PMN cytotoxicity assays

PMN-mediated cytotoxicity was analysed in chromium-51 [^51^Cr] release assays as previously described^131^. AbLecs and antibodies were added at varying concentrations. The CD47 blocking antibody hu5F9-IgG2σ was added at 20 µg ml^−1^. Effector cells and ^51^Cr-labelled target cells were added at a ratio of 40:1. After 3 h incubation at 5% CO_2_ and 37 °C, ^51^Cr release was measured in counts per minute (cpm) in a MikroBetaTrilux 1450 liquid scintillation and luminescence counter (PerkinElmer). Maximal ^51^Cr release was achieved by the addition of 2% v/v Triton-X 100 solution, while basal ^51^Cr release was measured in the absence of antibodies. Specific tumour cell lysis in % was calculated as follows:

$$\begin{array}{c}\mathrm{Cytotoxicity}\,( \% )=\\ (\mathrm{experimental}\,\mathrm{cpm}-\mathrm{basal}\,\mathrm{cpm})\,/\,(\mathrm{maximal}\,\mathrm{cpm}-\mathrm{basal}\,\mathrm{cpm})\,\times \,100\end{array}\,$$

### Mice

Wild-type C57BL/6 mice were purchased from The Jackson Laboratories and housed at the Comparative Bioscience Center at The Rockefeller University or in Stanford University vivaria. *SigE*^−/−^ mice were developed previously^20^. *SigE*^−/−^ Sig7/9 hFcγR mice were generated by crossing previously developed *SigE*^−/−^ Sig7/9^20^ and hFcγR^63^ mouse lines. *SigE*^−/−^ and *SigE*^−/−^ Sig7/9 hFcγR mice were bred and maintained at the Comparative Bioscience Center at The Rockefeller University or in Stanford University vivaria. Genotypes were confirmed using PCR in-house, as previously described^20,63^, or with validated qPCR probes (Transnetyx). The effects of these genetic modifications on Siglec-E/7/9 and human and mouse Fc gamma receptors have been extensively characterized^20,63^. All experiments carried out at The Rockefeller University were performed in compliance with federal laws and institutional guidelines and have been approved by The Rockefeller University Institutional Animal Care and Use Committee. Experiments involving animals carried out at Stanford University were similarly performed in compliance with federal laws as well as institutional guidelines and were approved by the Administrative Panel on Lab Animal Care (APLAC) under protocol number 31511. Husbandry was performed in accordance with the Guide for the Care and Use of Laboratory Animals and the Public Health Service Policy on Humane Care and Use of Laboratory Animals. Animals were housed under the following conditions: 12 h light, 12 h dark cycle; ambient temperature of 20–22 °C; and relative humidity of between 30 and 70%. All studies were performed on age-matched and sex-matched female and male mice.

### In vivo pharmacokinetics

Groups of two 8–12-month-old *SigE*^−/−^ Sig7/9 hFcγR mice were dosed with 1.5 nmol (~ 10 mg kg^−1^) trastuzumab or T7/9 AbLec intraperitoneally at *t* = 0. Blood samples were collected at *t* = 6, 24, 48, 72 and 96 h following injection. Serum was isolated by centrifugation at 4,000*g* for 10 min at 4 °C, and antibody or AbLec concentration in serum was assessed with western blot. For western blots, 0.5 μl of each serum sample was mixed with non-reducing SDS loading dye (non-reducing conditions), loaded onto a Criterion XT 4–12% Bis-Tris Protein Gel, 18-well (Bio-Rad) and run with XT-MES buffer at 180 V for 40–60 min. Proteins were then transferred to a nitrocellulose membrane using the Trans-Blot Turbo RTA Midi Nitrocellulose Transfer Kit (Bio-Rad) at 25 V for 14 min. The membrane was blocked in FACS buffer for 0.5–1 h at room temperature, stained with IRDye 800CW Goat anti-Human antibody (LI-COR; 1:10,000) in PBST for 0.5–1 h with shaking at room temperature, followed by three washes in PBST and one wash with PBS before imaging on an Odyssey CLx Imaging System (LI-COR). Antibody and AbLec concentrations in serum were quantified by densitometry and normalized to the average densitometry at *t* = 6 h. All serum samples from an individual mouse were analysed on the same blot to ensure accurate quantification.

### Lung colonization models

B16D5-HER2 lung colonization models were established by intravenously inoculating each mouse in the lateral tail vein with 0.3 or 0.5 × 10^6^ cells suspended in 200 μl PBS. For survival studies, groups of four or five 8–12-week-old wild-type and *SigE*^−/−^ mice were monitored for 30 days. For therapeutic efficacy studies, treatment groups of seven or eight 8–12-month-old *SigE*^−/−^ Sig7/9 hFcγR mice were randomized and received intraperitoneal injections of 2.25 nmol (~ 15 mg kg^−1^) of trastuzumab or T7/9 AbLecs every other day after tumour inoculation, and animal weights were measured every 3–4 days. On day 14, lungs were dissected, fixed with Fekete’s buffer (70% ethanol, 4% glacial acetic acid, 1.2% formalin in MilliQ water), and surface metastatic foci were quantified using a dissecting microscope.

### Macrophage phosphoproteomics analysis

Macrophage–cancer cell co-cultures in RPMI + 10% heat-inactivated FBS were prepared by adding 4 × 10^7^ K562-HER2 cells directly to a T75 flask containing macrophages on day 7–9 of differentiation from monocytes for a final effector to target cell ratio of ~2:1. Co-cultures were treated with PBS or equimolar (25 nM) quantities of Siglec-7 blocking antibody (1E8 clone^20^; Creative Biolabs), trastuzumab or T7 AbLec and incubated in a humidified incubator for 6 h at 5% CO_2_ and 37 °C. Following incubation, T75 flasks were washed three times with ice-cold PBS to remove non-adherent K562-HER2 cells, and the remaining macrophages were lysed in ice-cold RIPA buffer (1 ml per T75 flask) (Thermo Fisher, 89900) supplemented with phosphatase inhibitor (1:100; Cell Signaling Technology, 5870S) and HALT protease inhibitor (1:100; Thermo Fisher, 78429), per the manufacturer’s protocol. Macrophage lysates were sonicated for 30 s at 50% pulse to improve lysis efficiency before clarification by centrifugation at 4 °C for 10 min at 20,000*g*. Protein concentration was quantified by BCA (Thermo Fisher, 23227).

Digestion was performed on 100 µg protein using a mini S-trap protocol provided by the manufacturer (Protifi)^132^. Here, proteins were adjusted to 5% SDS and reduced with 5 mM dithiothreitol for 10 min at 95 °C. Cysteines were alkylated using 30 mM iodoacetamide for 45 min each at room temperature in the dark. The lysate was then acidified with phosphoric acid, adjusted to approximately 80–90% methanol with 100 mM TEAB in 90% methanol and loaded onto the S-trap column. Following washing with 100 mM TEAB in 90% methanol, trypsin (Promega) was added to the S-trap at a 20:1 protein to protease ratio for 90 min at 47 °C. Peptides from each lysate were labelled with 10-plex TMT (Tandem Mass Tags, Thermo Fisher Scientific) for 2 h at room temperature using recently published protocols^133,134^. Samples from each donor were kept in separate TMT experiments so that each donor used eight channels of a single 10-plex kit. The labelling scheme was the same for each donor: PBS vehicle replicates in channels 126 C and 127 C; αSig7 replicates in 127 N and 128 N (donor 2); empty channels for 128 C and 129 N; trastuzumab replicates in 129 C and 130 C; and T7 AbLec replicates in 130 N and 131 N. A test mix was run for each combined TMT experiment (that is, one per donor) to confirm >99% labelling efficiency and even distribution of signal across all channels before quenching of the TMT labelling reaction (0.5 µl 50% hydroxylamine reacted for 15 min). Peptides from each channel were then combined before phosphopeptide enrichment, which was performed as previously described^135^. In brief, 100 µl magnetic titanium(IV) immobilized metal ion affinity chromatography (Ti(IV)-IMAC, ReSyn Biosciences) beads were washed three times with 1 ml 80% acetonitrile/6% TFA (all washes were 1 ml)^136^. Peptides were dissolved in 1 ml 80% acetonitrile/6% TFA and gently vortexed with the TI(IV)-IMAC beads for 45 min. Beads were then washed with three 80% acetonitrile/6% TFA, one 80% acetonitrile, one 0.5 M glycolic acid/80% acetonitrile and two 80% acetonitrile washes. Peptides were eluted with 500 µl 50% acetonitrile, 1% ammonium hydroxide. Eluates were dried down in a speed vac and further cleaned up on Strata-X SPE cartridges (Phenomenex) by conditioning the cartridge with 1 ml ACN followed by 1 ml 0.2% formic acid in water. Peptides were resuspended in 0.2% formic acid in water and then loaded onto the cartridge, followed by a 1 ml wash with 0.2% formic acid in water. Peptides were eluted with 400 µl of 0.2% formic acid in 80% ACN and dried via lyophilization.

All samples were resuspended in 0.2% formic acid in water before liquid chromatography–MS/MS analysis. Enriched phosphopeptides were resuspended in 15 µl total with 4 µl injected per analysis. Triplicate injections were collected for all samples. All phosphopeptide mixtures were separated over a 25 cm EASY-Spray reversed-phase liquid chromatography column (75 µm inner diameter packed with 2 μm, 100 Å, PepMap C18 particles; Thermo Fisher Scientific). The mobile phases (A, water with 0.2% formic acid; B, acetonitrile with 0.2% formic acid) were driven and controlled by a Dionex Ultimate 3000 RPLC nano system (Thermo Fisher Scientific). An integrated loading pump was used to load peptides onto a trap column (Acclaim PepMap 100 C18, 5 µm particles, 20 mm length; Thermo Fisher Scientific) at 8 µl min^−1^, which was put in line with the analytical column 4 min into the gradient for the total protein samples. The gradient increased from 0% to 5% B over the first 4 min of the analysis, followed by an increase from 5% to 25% B from 4 to 158 min, an increase from 25% to 90% B from 158 to 162 min, isocratic flow at 90% B from 162 to 168 min and a re-equilibration at 0% for 12 min for a total analysis time of 180 min. Eluted phosphopeptides were analysed on an Orbitrap Fusion Tribrid MS system (Thermo Fisher Scientific). Precursors were ionized using an EASY-Spray ionization source (Thermo Fisher Scientific) held at +2.2 kV compared to ground, and the column was held at 40 °C. The inlet capillary temperature was held at 275 °C. Survey scans of peptide precursors were collected in the Orbitrap from 350–1,350 Th with an AGC target of 1,000,000, a maximum injection time of 50 ms and a resolution of 60,000 at 200 *m*/*z*. Monoisotopic precursor selection was enabled for peptide isotopic distributions, precursors of *z* = 2–5 were selected for data-dependent MS/MS scans for 2 s of cycle time, and dynamic exclusion was set to 30 s with a ±10 ppm window set around the precursor monoisotope. An isolation window of 1 Th was used to select precursor ions with the quadrupole. MS/MS scans were collected using higher-energy collisional dissociation at 30 normalized collision energy with an AGC target of 100,000 and a maximum injection time of 118 ms. Mass analysis was performed in the Orbitrap with a resolution of 60,000 with a first mass set at 100 Th.

All data were searched with the Andromeda search engine^137^ in MaxQuant^138^ (v.2.4.2), using the entire human proteome downloaded from Uniprot^139^ (reviewed, 20,428 entries). All raw files were searched together, and technical injections per donor-specific TMT experiment were labelled as fractions. Cleavage specificity was set to Trypsin/P with three missed cleavages allowed and variable modifications of phosphorylation on serine/threonine/tyrosine, oxidation of methionine and acetylation of the protein N-terminus with four maximum modifications per peptide. The experiment type was set to Reporter ion MS2, and only the TMT channels used (as described above) were selected to be included. The reporter ion mass tolerance was set to 0.3 Da, and the minimum reporter PIF score was set to 0.75. Defaults were used for the remaining settings, including peptide spectral match and protein false discovery rate thresholds of 0.01 and 20 ppm, 4.5 ppm and 20 ppm for first search MS1 tolerance, main search MS1 tolerance and MS2 product ion tolerance, respectively. Match between runs was not enabled.

Quantified phosphosites were then processed in Perseus^140^. Contaminants and reverse hits were removed, and results were filtered for phosphosites that had localization probabilities of >0.75; signal in all relevant TMT channels was required. Significance testing was performed using a two-tailed paired-sample *t*-test for pairwise comparisons with appropriate multiple comparisons correction. Gene Ontology term enrichment was performed using g:Profiler^141^. After processing in Perseus, data from pairwise comparisons of treatment conditions were exported as text files for interrogation using PTM-SEA^73^. For each phosphopeptide, a protein-phosphosite identifier was created by concatenating the UniProt ID, amino acid and phosphorylation position in the format {UniProt-ID}-{Amino Acid}{Position}. This identifier, along with enrichment data, was then reformatted into GCT (v.1.3) files using Morpheus^142^, following the specifications outlined at CLUE (https://clue.io/connectopedia/gct_format). GCT files were uploaded to the PTM-SEA module on GenePattern, alongside a UniProt-centric pathway database downloaded from the PTM-SEA GitHub repository. PTM-SEA results were retrieved from the web server and compiled by donor, treatment, pathway ID and enrichment score. Subsequently, the compiled PTM-SEA results were analysed in Python using Jupyter Notebook. Data were visualized using seaborn’s clustermap function (metric = ‘cityblock’, method = ‘average’), based on pathway fold-enrichment across donors and conditions.

### Small unilamellar vesicle preparation

Small unilamellar vesicles (SUVs) were prepared to functionalize silica beads for immune synapse imaging assays. For IgG functionalized silica beads, the following chloroform-suspended lipids were mixed and desiccated overnight to remove chloroform: 98.8% POPC (Avanti, 850457), 1% biotinyl cap PE (Avanti, 870273), 0.1% PEG5000-PE (Avanti, 880230) and 0.1% atto647-DOPE (ATTO-TEC, AD 647–161).

For beads conjugated with recombinant HER2, the following chloroform-suspended lipids were mixed and desiccated overnight to remove chloroform: 97.8% POPC (Avanti, 850457), 2% DGS-NTA (Avanti, 790404), 0.1% PEG5000-PE (Avanti, 880230) and 0.1% atto390-DOPE (ATTO-TEC, AD 390–161).

In both cases, the lipid sheets were resuspended in PBS and stored under inert gas. The lipids were then broken into SUVs by several rounds of freeze–thaws. The lipids were then stored at −80 °C under inert gas. To remove aggregated lipids, the solution was diluted to 2 mM and filtered through a 0.22 μM filter (Millipore, SLLG013SL) immediately before use.

### Silica bead functionalization

Silica beads with a 5.01 μm diameter (10% solids; Bangs Labs, SS05003, lot 16595) were washed several times with PBS, mixed with 1 mM SUVs in PBS and incubated at room temperature for 30 min with end-over-end mixing to allow for bilayer formation. Beads were then washed with PBS to remove excess SUVs and incubated in 0.2% casein (Sigma-Aldrich, C5890) in PBS for 15 min before protein coupling.

For IgG conjugated beads, anti-biotin Alexa Fluor 647-IgG (Jackson ImmunoResearch Laboratories 200-602-211, lot 156182) was added at 500 μM to a 10× dilution of beads (1% solids), unless otherwise indicated and incubated for 30 min with end-over-end mixing. When indicated, 10 μM ganglioside GD2 (Cayman Chemical, 25487) was added to beads for 30 min and incubated with end-over-end mixing.

For HER2 conjugated beads, recombinant human HER2 (Sino Biological, 10004-H08H4) was added at 1000 nM. Proteins were coupled to the bilayer for 30 min at room temperature with end-over-end mixing. When indicated, 10 μM ganglioside GD2 (Cayman Chemical, 25487) was added to beads for 30 min and incubated with end-over-end mixing. Trastuzumab or T7 AbLec was then added to beads at 500 μM for 30 min and incubated with end-over-end mixing.

### Macrophage and immune synapse imaging assays

Approximately 50,000 RAW264.7-Siglec-7–mGFP macrophages were plated in one well of a 96-well glass-bottom MatriPlate (Brooks, MGB096-1-2-LG-L) between 12 and 24 h before the experiment.

For phagocytosis assays, ~8 × 10^5^ beads were added to wells and engulfment was allowed to proceed for 30 min. The cells were imaged using spinning disc microscopy (×40/0.95 NA Plan Apo air). Internalized particles were identified by their fluorescent supported lipid bilayer, and counted in ImageJ by a blinded analyst using the Blind-Analysis-Tools-1.0 ImageJ plug-in.

For immune synapse imaging assays, ~8 × 10^5^ beads were added to wells, and individual bead contacts were imaged through multiple z planes at various stages of phagocytosis using spinning disk confocal microscopy (×100/1.49 NA oil immersion objective). Siglec-7–mGFP enrichment at bead contacts compared to the cell cortex was determined using the line analyser tool in ImageJ and was normalized to a membrane-bound control protein (CAAX–mCherry).

### Isolation and activation of human T cells

PBMCs were isolated from LRS chambers, cryostocks were prepared at 2–4 × 10^7^ cells in 90% heat-inactivated FBS + 10% DMSO and stocks were stored in liquid nitrogen vapour until use, as described above. Human T cells were isolated from thawed cryostocks using an EasySep CD3^+^ T cell isolation kit (StemCell Technologies). T cells were cultured at a 1:2 cell to bead ratio with cultured αCD3/αCD28 activator beads (Thermo Fisher Scientific) and 50 ng ml^−1^ recombinant IL-2 (BioLegend) in RPMI + 10% heat-inactivated FBS for 3 days before use.

### Statistical analysis

Statistical analysis was performed in GraphPad Prism (v.10.4) and using Hierarch^143^ (v.1.1.6). Unless otherwise noted, the following statistical tests were used for data analysis. In binding assays, NK cell cytotoxicity experiments, phagocytosis experiments and Siglec expression analyses, ordinary one-way ANOVAs were performed with Tukey’s multiple comparisons test to compare treatment groups. Unpaired, two-tailed *t*-tests were performed to determine statistical significance when comparing two treatment conditions. Hierarch’s many-sample, multiple hypothesis test with Benjamini–Hochberg multiple comparisons correction was used to determine statistical significance from biological replicates collected across multiple donors. All results were confirmed with multiple biological replicates as indicated in the figure legends. All data were tested for outliers using GraphPad Outlier Calculator (http://graphpad.com/quickcalcs/Grubbs1.cfm), and significant outliers (*P* < 0.05) were removed.

### Reporting summary

Further information on research design is available in the Nature Portfolio Reporting Summary linked to this article.

## Online content

Any methods, additional references, Nature Portfolio reporting summaries, source data, extended data, supplementary information, acknowledgements, peer review information; details of author contributions and competing interests; and statements of data and code availability are available at https://doi.org/10.1038/s41587-025-02884-6.

## Supplementary information

Supplementary Information Supplementary Figures 1–2.

Reporting Summary

Supplementary Table 1 AbLec DNA and amino acid sequences.

Supplementary Table 2 LC-MS/MS sequencing of AbLecs.

Supplementary Table 3 Antibodies used in this study for flow cytometry.

Supplementary Video 1 Phase contrast and fluorescence microscopy images of primary human macrophage/SK-BR-3 cell co-cultures treated with Siglec-7-Fc from t = 0–5 h. SK-BR-3 cells were stained with the pHrodo red pH sensitive dye which fluoresces upon SK-BR-3 phagocytosis by macrophages. Images are representative of n = 3 biological replicates, scale bar represents 400 μm.

Supplementary Video 2 Phase contrast and fluorescence microscopy images of primary human macrophage/SK-BR-3 cell co-cultures treated with trastuzumab from t = 0–5 h. SK-BR-3 cells were stained with the pHrodo red pH sensitive dye which fluoresces upon SK-BR-3 phagocytosis by macrophages. Images are representative of n = 3 biological replicates, scale bar represents 400 μm.

Supplementary Video 3 Phase contrast and fluorescence microscopy images of primary human macrophage/SK-BR-3 cell co-cultures treated with T7 AbLec from t = 0–5 h. SK-BR-3 cells were stained with the pHrodo red pH sensitive dye which fluoresces upon SK-BR-3 phagocytosis by macrophages. Images are representative of n = 3 biological replicates, scale bar represents 400 μm.

## Source data

Source Data Fig. 1 Statistical source data

Source Data Fig. 2 Statistical source data

Source Data Fig. 3 Statistical source data

Source Data Fig. 4 Statistical source data

Source Data Fig. 5 Statistical source data

Source Data Extended Data Fig. 1 Statistical source data

Source Data Extended Data Fig. 2 Statistical source data

Source Data Extended Data Fig. 3 Statistical source data

Source Data Extended Data Fig. 4 Statistical source data

Source Data Extended Data Fig. 5 Statistical source data

Source Data Extended Data Fig. 6 Statistical source data

Source Data Extended Data Fig. 7 Statistical source data

Source Data Extended Data Fig. 8 Statistical source data

Source Data Extended Data Fig. 9 Statistical source data

Source Data Extended Data Fig. 10 Statistical source data

## Data Availability

Source data used to generate manuscript figures are provided with this paper or available from a public data repository. MS datasets have been deposited to the ProteomeXchange Consortium (http://proteomecentral.proteomexchange.org) through the PRIDE partner repository^144^ with the dataset identifier PXD063934. Plasmids used in this work are available from Addgene; details are listed in Supplementary Table 1. Any unique materials presented in the manuscript may be available from the authors upon reasonable request and through a materials transfer agreement. Stanford University will use the UBMTA or UBMTA-like terms for the transfer of materials described in this manuscript to the extent possible. Source data are provided with this paper.
