# Supplementary material for: Antibody-lectin chimeras for glyco-immune checkpoint blockade
Source: Nat Biotechnol. 2025 Dec 16;44(10):1657–71. doi: 10.1038/s41587-025-02884-6 (PMC13041563; doi:10.1038/s41587-025-02884-6)
Supplement: Supplementary file 1 [file 41587_2025_2884_MOESM1_ESM.pdf]

nature biotechnology

Supplementary information https://doi.org/10.1038/s41587-025-02884-6

# Antibody-lectin chimeras for glyco-immune checkpoint blockade

In the format provided by the authors and unedited

## Supplementary Information for

# Antibody-lectin chimeras for glyco-immune checkpoint blockade

Jessica C. Stark[1,2,3,†], Melissa A. Gray[4], Itziar Ibarlucea-Benitez[5], Marta Lustig[6], Annalise Bond[7], Brian Cho[1,3,*], Ishika Govil[2,3,*], Tran Luu[1,3,*], Megan J. Priestley[1,3,*], Tim S. Veth[8], Wesley J. Errington[9], Bence Bruncsics[10], Mikaela K. Ribi[4], Leo A. Williams[1,3], Casim A. Sarkar[9], Simon Wisnovsky[11], Nicholas M. Riley[8], Meghan A. Morrissey[7], Thomas Valerius[6], Jeffrey V. Ravetch[5], Carolyn R. Bertozzi[4,12,†]

[1]Department of Biological Engineering, MIT, Cambridge, MA, USA
[2]Department of Chemical Engineering, MIT, Cambridge, MA, USA
[3]Koch Institute for Integrative Cancer Research, MIT, Cambridge, MA, USA
[4]Department of Chemistry and Sarafan ChEM-H, Stanford University, Stanford, CA, USA
[5]Laboratory of Molecular Genetics and Immunology, The Rockefeller University, New York, NY, USA
[6]Division of Stem Cell Transplantation and Immunotherapy, Department of Medicine II, Christian Albrechts University Kiel and University Medical Center Schleswig-Holstein, Kiel, Germany
[7]Department of Molecular Cellular and Developmental Biology, University of California, Santa Barbara, CA, USA
[8]Department of Chemistry, University of Washington, Seattle, WA, USA
[9]Department of Biomedical Engineering, University of Minnesota, Minneapolis, MN, USA
[10]Department of Measurement and Information Systems, Budapest University of Technology and Economics, Budapest, Hungary
[11]Faculty of Pharmaceutical Sciences, University of British Columbia, Vancouver, BC, Canada
[12]Howard Hughes Medical Institute, Stanford, CA, USA
[*] authors contributed equally
[†] correspondence to: jcstark@mit.edu, bertozzi@stanford.edu

**Description:** Supplementary Figures 1-2.

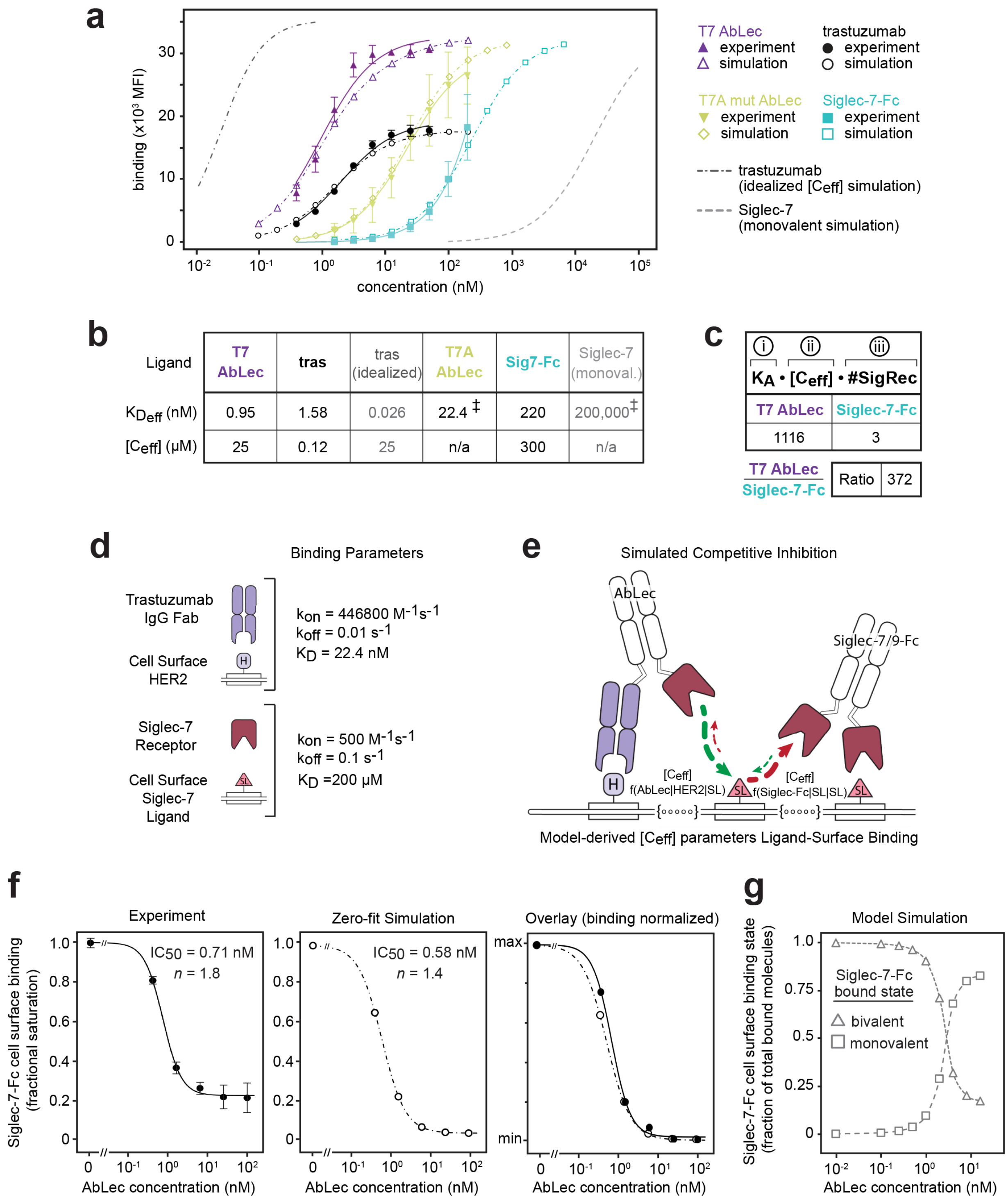


**Supplementary Figure 1. Modeling AbLec binding and Siglec receptor blockade.**

(**a**) Binding of Siglec-7-Fc, trastuzumab, T7 AbLec, and T7A AbLec to K562-HER2 cells was modeled using *MVsim* (Bruncsics, et al. *Nat Commun*, 2022). A modeling routine was constructed to simulate the enhancement to binding affinity ($K_{D,eff}$) that was achieved through the cooperative binding of a bivalent, monospecific antibody (trastuzumab; black traces); a bivalent, monospecific Siglec receptor (Siglec-7-Fc; blue traces); and a bivalent, bispecific AbLec (T7 AbLec; purple traces) to a surface consisting of both

HER2 and Siglec-7 ligands. The modeling routine was used to perform curve fitting via parameter estimation of avidities and effective concentrations that drive multivalent binding enhancements. These enhancements in cooperative avidity were assessed relative to the binding of a non-cooperative, monovalent reference (T7A AbLec; green traces). Additional simulations were performed to show relevant upper and lower bounds of binding, from a theoretical trastuzumab ligand with high, AbLec-like cooperativity (grey dash-dotted line) to a monomeric Siglec-7 receptor with experimentally indeterminable weak binding (grey dashed line). Experimental data are mean ± s.d. of n = 3 biological replicates.

(**b**) The *MVsim* model of T7 AbLec binding was used to estimate the effective concentrations ($[C_{eff}]$) that drive cooperative ligand binding to HER2 antigens and Siglec-7 ligands on K562-HER2 cells. The modeling routine in (**a**) treated the avidity enhancements relative to the monovalent reference as fitted parameters from which the spatial proximities of either two HER2 antigens, one HER2 antigen and one Siglec-7 ligand, or two Siglec-7 ligands at the K562-HER2 cell surface could be estimated. These parameter estimates are presented here as the avidity ($K_{D,eff}$) and the effective concentration of the second arm relative to its target after binding of the first arm ($[C_{eff}]$). Monovalent ligands (T7A AbLec and Siglec-7 monovalent) do not exhibit any avidity enhancements, so only their monovalent $K_D$ values are reported (‡). A higher $[C_{eff}]$ indicates a higher probability that an antibody or AbLec can engage in a cooperative, bivalent interaction at the cell surface due to proximity of its two antigens/ligands.

(**c**) The experimental and model-derived parameters of ligand-cell surface binding in (**a**) and (**b**) were used to compute a potency metric for Siglec-7 delivery, which is defined here as the product of: (**i**) the binding affinity of the first arm ($K_A = 1/K_D$), (**ii**) the effective concentration of the second arm relative to its target, and (**iii**) the number of Siglec-7 receptors recruited to the cell surface (1 per T7 AbLec molecule and 2 per Siglec-7-Fc molecule).

(**d, e**) Monovalent binding parameters and system setup for the AbLec/Siglec-7-Fc competition model. The base binding model was parameterized with kinetic, affinity, and $[C_{eff}]$ values derived from experimental data, model-guided fits, and the literature (Yamaji, et al. *J Biol Chem*, 2002; Attrill, et al. *J Biol Chem*, 2006). The concentrations of AbLec (0-100 nM) and Siglec-7-Fc (200 nM), and the simulated architectures of the ligands, cell surface, and treatment of the binding competition were taken directly from those used in the corresponding experiments (**Fig. 1i**).

(**f**) The experimental dose-response curve (**left**) compared to the model simulation with no additional fitted parameters (**middle**) for the fraction of Siglec-7-Fc surface binding as a function of T7 AbLec concentration. $IC_{50}$ is the T7 AbLec concentration at which half of the competable Siglec-7-Fc binding is lost; n is the Hill coefficient of the response curve. Overlay of the experimental and simulated dose-response curves (**right**) with each normalized to its respective minimum and maximum values show good agreement between experimental and simulated results. Experimental data are mean ± s.d. of n = 3 biological replicates.

(**g**) *MVsim* enables more detailed analysis of the microstates that comprise the overall binding signal by parsing Siglec-7-Fc species that are bivalently bound (trace with triangles) and monovalently bound (trace with squares) as a function of T7 AbLec concentration. Low nanomolar concentrations of T7 AbLec can effectively prevent both arms of a bivalently bound Siglec-7-Fc from engaging, resulting in monovalent Siglec-7-Fc species that are displaced or very weakly bound.

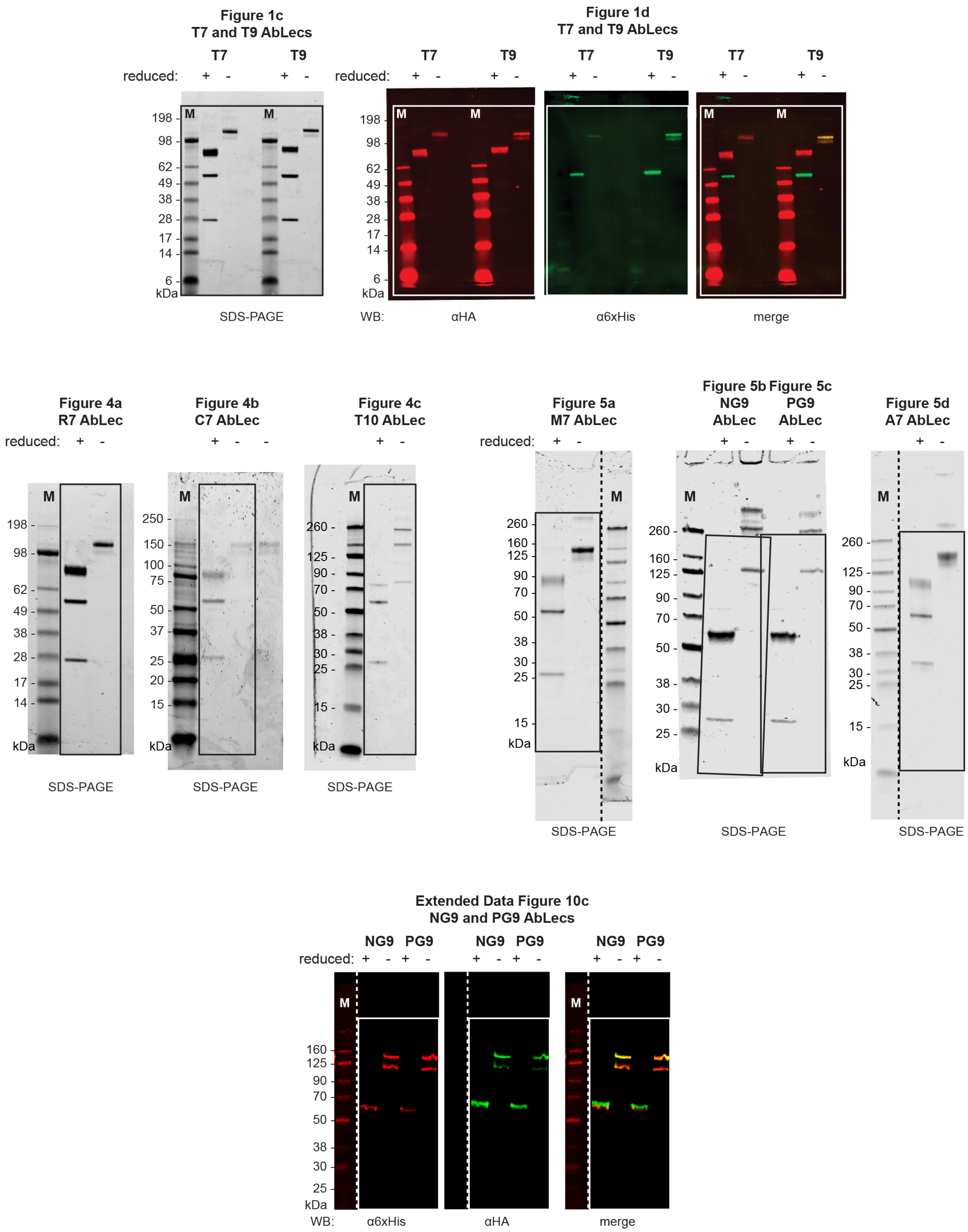


**Supplementary Figure 2.** Raw Western blot source images. Boxes indicate cropping for representative images. Dotted lines indicate molecular weight markers are from the same gel or blot. Corresponding Figures are indicated.
