# Supplementary material for: Antibody-lectin chimeras for glyco-immune checkpoint blockade
Source: Nat Biotechnol. 2025 Dec 16;44(10):1657–71. doi: 10.1038/s41587-025-02884-6 (PMC13041563; doi:10.1038/s41587-025-02884-6)
Supplement: Supplementary file 2 [file 41587_2025_2884_MOESM2_ESM.pdf]

nature portfolio

Corresponding author(s): Carolyn R. Bertozzi

Last updated by author(s): April 24, 2024

Reporting Summary

Nature Portfolio wishes to improve the reproducibility of the work that we publish. This form provides structure for consistency and transparency in reporting. For further information on Nature Portfolio policies, see our Editorial Policies and the Editorial Policy Checklist.

Statistics

For all statistical analyses, confirm that the following items are present in the figure legend, table legend, main text, or Methods section.

n/a	Confirmed	
☐	☒	The exact sample size (*n*) for each experimental group/condition, given as a discrete number and unit of measurement
☐	☒	A statement on whether measurements were taken from distinct samples or whether the same sample was measured repeatedly
☐	☒	The statistical test(s) used AND whether they are one- or two-sided<br>*Only common tests should be described solely by name; describe more complex techniques in the Methods section.*
☒	☐	A description of all covariates tested
☐	☒	A description of any assumptions or corrections, such as tests of normality and adjustment for multiple comparisons
☐	☒	A full description of the statistical parameters including central tendency (e.g. means) or other basic estimates (e.g. regression coefficient) AND variation (e.g. standard deviation) or associated estimates of uncertainty (e.g. confidence intervals)
☐	☒	For null hypothesis testing, the test statistic (e.g. *F*, *t*, *r*) with confidence intervals, effect sizes, degrees of freedom and *P* value noted<br>*Give P values as exact values whenever suitable.*
☒	☐	For Bayesian analysis, information on the choice of priors and Markov chain Monte Carlo settings
☒	☐	For hierarchical and complex designs, identification of the appropriate level for tests and full reporting of outcomes
☒	☐	Estimates of effect sizes (e.g. Cohen's *d*, Pearson's *r*), indicating how they were calculated

Our web collection on statistics for biologists contains articles on many of the points above.

Software and code

Policy information about availability of computer code

Data collection	BD LSR II and Symphony instruments were used for flow cytometry analysis. An IncuCyte® S3 Live-Cell Analysis System (Sartorius) was used for in vitro phagocytosis assays. Mass spectrometry sequencing of AbLecs and macrophage phosphoproteomics were performed via LC separation using a Dionex Ultimate 3000 RPLC nano system (Thermo Fisher Scientific) followed by MS/MS analysis using an Orbitrap Fusion Tribrid MS system (Thermo Fisher Scientific). Thermal shift assays were run on a Prometheus Panta nanoDSF instrument (NanoTemper Technologies).
Data analysis	FlowJo v10.0 (Tree Star) was used for flow cytometry analysis. GraphPad Prism (v10.4) was used for statistical analysis and curve-fitting. Gel and Western blot images were analyzed using Image Studio Lite version 5.2.5. IncuCyte image analysis software was used for analysis of time-lapse microscopy images. Mass spectrometry data were processed using Byonic in MaxQuant version 2.4.2.

For manuscripts utilizing custom algorithms or software that are central to the research but not yet described in published literature, software must be made available to editors and reviewers. We strongly encourage code deposition in a community repository (e.g. GitHub). See the Nature Portfolio guidelines for submitting code & software for further information.

Data

Policy information about availability of data

All manuscripts must include a data availability statement. This statement should provide the following information, where applicable:

- Accession codes, unique identifiers, or web links for publicly available datasets
- A description of any restrictions on data availability
- For clinical datasets or third party data, please ensure that the statement adheres to our policy

All primary data for main text and supplementary figures are available from the authors upon reasonable request. Any unique materials presented in the manuscript may be available from the authors upon reasonable request and through a materials transfer agreement. Plasmids encoding AbLec genes are available through Addgene and their DNA and amino acid sequences are listed in Supplemental Table 1.

Research involving human participants, their data, or biological material

Policy information about studies with human participants or human data. See also policy information about sex, gender (identity/presentation), and sexual orientation and race, ethnicity and racism.

Reporting on sex and gender	n/a
Reporting on race, ethnicity, or other socially relevant groupings	n/a
Population characteristics	n/a
Recruitment	n/a
Ethics oversight	n/a

Note that full information on the approval of the study protocol must also be provided in the manuscript.

Field-specific reporting

Please select the one below that is the best fit for your research. If you are not sure, read the appropriate sections before making your selection.

☒ Life sciences ☐ Behavioural & social sciences ☐ Ecological, evolutionary & environmental sciences

For a reference copy of the document with all sections, see nature.com/documents/nr-reporting-summary-flat.pdf

Life sciences study design

All studies must disclose on these points even when the disclosure is negative.

Sample size	Sample sizes for in vitro immune killing assays were designed according to standards in the literature. An independent statistical method was not used to determine sample size. In our experience with in vitro ADCC/ADCP assays, we have found that assaying immune cells from 3 donors is sufficient to reflect variability among donors for studies of antibody efficacy.<br>In survival studies, we expect a 50% difference between means of control and experimental groups, with a standard deviation of 25%. Given a power of 0.85 and p value of 0.05, we required 5 animals per group.<br>For lung colonization experiments, we expect a 25% difference between the means of control and experimental groups, with a standard deviation of 15%. Given a power of 0.85 and a p value of 0.05, we required 8 mice per group.
Data exclusions	All data were tested for outliers using GraphPad Outlier Calculator (http://graphpad.com/quickcalcs/Grubbs1.cfm) and significant outliers ($p<0.05$) were removed. This exclusion criteria was pre-established.
Replication	All studies were performed with multiple biological replicates, as described in the Figures, Figure Legends, and Methods. In vitro ADCC/ADCP assays were replicated using immune cells from multiple human/mouse donors, as described in the Figures, Figure Legends, and Methods.
Randomization	No randomization was performed.
Blinding	Lungs were randomized and identified only by mouse ID number (i.e., no annotation of treatment group) during quantification of metastatic foci.

Reporting for specific materials, systems and methods

We require information from authors about some types of materials, experimental systems and methods used in many studies. Here, indicate whether each material, system or method listed is relevant to your study. If you are not sure if a list item applies to your research, read the appropriate section before selecting a response.

Materials & experimental systems

n/a	Involved in the study
☐	☒ Antibodies
☐	☒ Eukaryotic cell lines
☒	☐ Palaeontology and archaeology
☐	☒ Animals and other organisms
☒	☐ Clinical data
☒	☐ Dual use research of concern
☒	☐ Plants

Methods

n/a	Involved in the study
☒	☐ ChIP-seq
☐	☒ Flow cytometry
☒	☐ MRI-based neuroimaging

Antibodies

Antibodies used

Antibody clones, suppliers, and concentrations used are provided in the Methods section and in Supplemental Table 2.

Western blot
anti-HA Tag Polyclonal Antibody, clone SG77, Thermo Fisher Scientific #715500, 1:500
anti-6xHis antibody, clone J099B12, BioLegend #652502, 1:2000
IRDye® 800CW Goat anti-Mouse, LI-COR #926-32210, 1:15,000
IRDye® 680RD Goat anti-Rabbit, LI-COR, #926-68071 1:15,000

Flow cytometry
Siglec-7-Fc, R&D Systems #1138-SL-050, 7.5 nM
Siglec-9-Fc, R&D Systems #1139-SL-050, 7.5 nM
human IgG1 isotype Fc, R&D Systems #110-HG-100, 7.5 nM
trastuzumab, Selleck Chem #A2007 or expressed in house, 7.5 nM
rituximab, Selleck Chem #A2009 or expressed in house, 7.5 nM
cetuximab, Selleck Chem #A2000 or expressed in house, 7.5 nM
anti-GD2, BioXCell #BE0318, 7.5 nM
hIgG1 isotype control, BioXCell #BE0297, 7.5 nM
Alexa Fluor 647 (AF647)-labeled donkey anti-human IgG antibody, Jackson ImmunoResearch #709-605-098, 7.5 nM
Biotin-labeled MALII lectin, Vector Labs #B-1265, 10 µg/mL
AF647-Streptavidin, Thermo Fisher Scientific #S21374, 1 µg/mL
CD11b, clone M1/70, BioLegend #101228, 10 µg/mL
FcγRI (CD64), clone 10.1, BioLegend #305006, 10 µg/mL
FcγRIIa (CD32a), clone IV.3, STEMCELL Technologies #60012FI, 10 µg/mL
FcγRIIb (CD32b), clone 2B6, expressed in house, 10 µg/mL
FcγRIII (CD16), clone 3G8, BioLegend #302006, 10 µg/mL
SIRPα, clone 15-414, BioLegend #372103, 10 µg/mL
CD47, clone CC2C6, BioLegend #323108, 10 µg/mL
CD3, clone OKT3, BioLegend #317344, 10 µg/mL
CD69, clone FN50, BioLegend #310914, 10 µg/mL
PD-1, clone EH12.2H7, BioLegend #329906, 10 µg/mL
TIM-3, clone F38-2E2, BioLegend #345011, 10 µg/mL
PD-L1, clone MIH2, BioLegend #393609, 10 µg/mL

Immune cell assays
Siglec-7 antagonist antibody, clone 1E8, Creative Biolabs #HPAB-2548LY, 12.5-25 µg/mL
Siglec-9 antagonist antibody, clone mAbA, Creative Biolabs #HPAB-M0648-YC, 12.5-25 µg/mL
human IgG1 isotype antibody, BioXCell #BE0297, 10 µg/mL
rat IgG2a isotype antibody, BioXCell #BE0089, 10 µg/mL
CD47 antagonist antibody, clone B6H12, BioXCell #BE0019-1, 10 µg/mL
CD16 blocking antibody, clone 3G8, BioLegend #302002, 10 µg/mL
anti-biotin AlexaFluor647-IgG, Jackson ImmunoResearch Laboratories #200-602-211, 0.5 mM
Galectin-9-Fc, Sino Biological #11147-H01H, 25 nM
nivolumab, BioXCell #SIM0003, 0.1 nM
pembrolizumab, BioXCell #SIM0010, 0.1 nM

Validation

Beyond validation performed by the manufacturer, we validated antibody-specific staining in our lab using isotype and/or unstained controls for all antibodies used in this study.

Western blot
anti-HA Tag Polyclonal Antibody, Thermo Fisher Scientific #715500, was validated here: https://www.thermofisher.com/antibody/product/HA-Tag-Antibody-clone-SG77-Polyclonal/71-5500
anti-6xHis antibody, BioLegend #652502, was validated here: https://www.biolegend.com/en-us/products/purified-anti-his-tag-antibody-7841
IRDye® 800CW Goat anti-Mouse, LI-COR #926-32210, was validated here: https://www.licor.com/bio/reagents/irdye-800cw-goat-anti-mouse-igg-secondary-antibody

IRDye® 680RD Goat anti-Rabbit, LI-COR #926-68071, was validated here: https://www.licor.com/bio/reagents/irdye-680rd-goat-anti-rabbit-igg-secondary-antibody

Flow cytometry
Siglec-7-Fc, R&D Systems #1138-SL-050, was validated here: https://www.rndsystems.com/products/recombinant-human-siglec-7-cd328-fc-chimera-protein-cf_1138-sl
Siglec-9-Fc, R&D Systems #1139-SL-050, was validated here: https://www.rndsystems.com/products/recombinant-human-siglec-9-fc-chimera-protein-cf_1139-sl
human IgG1 isotype Fc, R&D Systems #110-HG-100, was validated here: https://www.rndsystems.com/products/recombinant-human-igg1-fc-protein-cf_110-hg
trastuzumab, Selleck Chem #A2007 or expressed in house, was validated here: https://www.selleckchem.com/products/trastuzumab.html
rituximab, Selleck Chem #A2009 or expressed in house, was validated here: https://www.selleckchem.com/products/rituximab.html
cetuximab, Selleck Chem #A2000 or expressed in house, was validated here: https://www.selleckchem.com/products/cetuximab.html
anti-GD2, BioXCell #BE0318, was validated here: https://bioxcell.com/invivomab-anti-human-ganglioside-gd2-be0318
hIgG1 isotype control, BioXCell #BE0297, was validated here: https://bioxcell.com/invivomab-human-igg1-isotype-control-be0297
Alexa Fluor 647 (AF647)-labeled donkey anti-human IgG antibody, Jackson ImmunoResearch #709-605-098, was validated here: https://www.jacksonimmuno.com/catalog/products/709-605-098
Biotin-labeled MALII lectin, Vector Labs #B-1265, was validated here: https://vectorlabs.com/products/biotinylated-maackia-amurensis-lectin-ii
AF647-Streptavidin, Thermo Fisher Scientific #S21374, was validated here: https://www.thermofisher.com/order/catalog/product/S21374
CD11b, clone M1/70, BioLegend #101228, was validated here: https://www.biolegend.com/fr-fr/sean-tuckers-tests/percp-cyanine5-5-anti-mouse-human-cd11b-antibody-4257
FcγRI (CD64), clone 10.1, BioLegend #305006, was validated here:
FcγRIIa (CD32a), clone IV.3, STEMCELL Technologies #60012FI, was validated here: https://www.stemcell.com/products/anti-human-cd32-antibody-clone-iv-3.html
FcγRIII (CD16), clone 3G8, BioLegend #302006, was validated here: https://www.biolegend.com/fr-fr/products/fitc-anti-human-cd16-antibody-567
SIRPα, clone 15-414, BioLegend #372103, was validated here: https://www.biolegend.com/fr-fr/products/pe-anti-human-cd172a-sirpalpha-antibody-14133
CD47, clone CC2C6, BioLegend #323108, was validated here: https://www.biolegend.com/fr-fr/products/pe-anti-human-cd47-antibody-3708
CD3, clone OKT3, BioLegend #317344, was validated here: https://www.biolegend.com/fr-fr/products/brilliant-violet-421-anti-human-cd3-antibody-11976
CD69, clone FN50, BioLegend #310914, was validated here: https://www.biolegend.com/fr-fr/products/apc-cyanine7-anti-human-cd69-antibody-1917
PD-1, clone EH12.2H7, BioLegend #329906, was validated here: https://www.biolegend.com/fr-fr/products/pe-anti-human-cd279-pd-1-antibody-4412
TIM-3, clone F38-2E2, BioLegend #345011, was validated here: https://www.biolegend.com/fr-fr/products/apc-anti-human-cd366-tim-3-antibody-8302
PD-L1, clone MIH2, BioLegend #393609, was validated here: https://www.biolegend.com/fr-fr/products/apc-anti-human-cd366-tim-3-antibody-8302

Immune cell assays
Siglec-7 antagonist antibody, clone 1E8, Creative Biolabs #HPAB-2548LY, was validated here: https://pubmed.ncbi.nlm.nih.gov/34155121/
Siglec-9 antagonist antibody, clone mAbA, Creative Biolabs #HPAB-M0648-YC, was validated here: https://pubmed.ncbi.nlm.nih.gov/34155121/
human IgG1 isotype antibody, BioXCell #BE0297, was validated here: https://bioxcell.com/invivomab-human-igg1-isotype-control-be0297
rat IgG2a isotype antibody, BioXCell #BE0089, was validated here: https://bioxcell.com/invivomab-rat-igg2a-isotype-control-anti-trinitrophenol-be0089
CD47 antagonist antibody, clone B6H12, BioXCell #BE0019-1, was validated here: https://bioxcell.com/invivomab-anti-human-cd47-be0019-1
CD16 blocking antibody, clone 3G8, BioLegend #302002, was validated here: https://www.biolegend.com/fr-fr/products/purified-anti-human-cd16-antibody-571
anti-biotin AlexaFluor647-IgG, Jackson ImmunoResearch Laboratories #200-602-211, was validated here: https://www.jacksonimmuno.com/catalog/products/200-602-211
Galectin-9-Fc, Sino Biological #11147-H01H, was validated here: https://www.sinobiological.com/recombinant-proteins/human-galectin-9-11147-h01h
nivolumab, BioXCell #SIM0003, was validated here: https://bioxcell.com/invivosim-anti-human-pd-1-nivolumab-biosimilar-sim0003
pembrolizumab, BioXCell #SIM0010, was validated here: https://bioxcell.com/invivosim-anti-human-pd-1-pembrolizumab-biosimilar-sim0010

Eukaryotic cell lines

Policy information about cell lines and Sex and Gender in Research

Cell line source(s)

SK-BR-3, HCC-1954, K562, MDA-MB-231, and Ramos cells were purchased from the American Type Culture Collection (ATCC). HER2+ B16D5 cells were a gift from the Weiner laboratory (Lombardi Cancer Center). Expi293F cells were a gift from the Kim lab (Stanford).

Authentication

Cell lines were not independently authenticated beyond the identity provided by ATCC or collaborators.

Mycoplasma contamination	Cell lines tested negative in quarterly mycoplasma detection assays using the ATCC universal mycoplasma detection kit.
Commonly misidentified lines (See ICLAC register)	None of the cell lines used in this study are listed in the database of commonly misidentified cell lines.

Animals and other research organisms

Policy information about studies involving animals; ARRIVE guidelines recommended for reporting animal research, and Sex and Gender in Research

Laboratory animals	SigE-/- mice used in this study were developed previously (Ibarlucea-Benitez, PNAS, 2021). SigE-/- Sig7/9 hFcγR mice were generated by crossing previously developed SigE-/- Sig7/9 (Ibarlucea-Benitez, PNAS, 2021) and hFcγR (Smith, PNAS, 2012) mouse lines. Genotypes were confirmed using PCR in house, as previously described or with validated qPCR probes (Transnetyx). The effects of these genetic modifications on Siglec-E/7/9 and human and mouse Fc gamma receptors have been extensively characterized. Mice were bred and maintained at the Comparative Bioscience Center at The Rockefeller University or in Stanford University vivaria.
Wild animals	This study did not involve wild animals.
Reporting on sex	All studies were performed using age- and sex-matched female and male mice. For survival studies, groups of n = 4-5 8-12 week old WT and SigE-/- mice were used. For therapeutic efficacy studies, treatment groups of n = 7-8 8-12 month old SigE-/- Sig7/9 hFcγR mice were used. For PK studies, groups of n = 2 8-12 month old SigE-/- Sig7/9 hFcγR mice were used.
Field-collected samples	This study did not involve field-collected samples.
Ethics oversight	All experiments carried out at The Rockefeller University were performed in compliance with federal laws and institutional guidelines and have been approved by The Rockefeller University Institutional Animal Care and Use Committee. Experiments involving animals carried out at Stanford University were similarly performed in compliance with federal laws as well as institutional guidelines and were approved by the Administrative Panel on Laboratory Animal Care (APLAC) under protocol number 31511.

Note that full information on the approval of the study protocol must also be provided in the manuscript.

Plants

Seed stocks	n/a
Novel plant genotypes	n/a
Authentication	n/a

Flow Cytometry

Plots

Confirm that:

- [x] The axis labels state the marker and fluorochrome used (e.g. CD4-FITC).
- [x] The axis scales are clearly visible. Include numbers along axes only for bottom left plot of group (a 'group' is an analysis of identical markers).
- [x] All plots are contour plots with outliers or pseudocolor plots.
- [x] A numerical value for number of cells or percentage (with statistics) is provided.

Methodology

Sample preparation	Lectin flow cytometry<br>Cells were pelleted by centrifugation, washed once with phosphate-buffered saline (PBS), and resuspended in FACS buffer (PBS with 0.5% bovine serum albumin). Siglec-Fc or human IgG1 isotype Fc (R&D Systems) precomplexes with Alexa Fluor 647 (AF647)-labeled donkey anti-human IgG antibody (Jackson ImmunoResearch) were prepared at equimolar concentrations (7.5 nM) in FACS buffer. Biotin-labeled MALII lectin (Vector Labs, 10 μg/mL) was precomplexed with AF647-Streptavidin (Thermo Fisher Scientific, 1 μg/mL) in FACS buffer. Staining was performed for 30 min on ice at 1 × 106 cells/mL. Cells were subsequently washed twice with ice-cold FACS buffer, resuspended in FACS buffer containing 100 nM SytoxGreen or 1 μM SytoxBlue (Thermo Fisher Scientific), and analyzed by flow cytometry (BD LSR II or BD Symphony).<br><br>Antibody flow cytometry

Cells were pelleted by centrifugation, washed once with phosphate-buffered saline (PBS), and resuspended in FACS buffer, as above. To quantify HER2, GD2, CD20, or EGFR expression, cells were stained with 7.5 nM antibody (trastuzumab, dinutuximab, rituximab, or cetuximab, respectively) or a human IgG1 isotype control in FACS buffer for 30 min on ice at 1 × 106 cells/mL. Cells were subsequently washed twice with ice-cold FACS buffer, resuspended in FACS buffer with 7.5 nM AF647-labeled donkey anti-human IgG secondary antibody (Jackson ImmunoResearch) and stained for 30 min on ice. Following incubation with the secondary antibody, cells were washed twice with ice-cold FACS buffer, resuspended in FACS buffer containing 100 nM SytoxGreen or 1 µM SytoxBlue (Thermo Fisher Scientific), and analyzed via flow cytometry (BD LSR II or BD Symphony). For all other flow experiments, cells were stained with 10 µg/mL dye-labeled antibodies or the appropriate isotype controls in FACS buffer for 30 min at 4 ºC. All antibody clones used are listed in Supplemental Table 2. Following incubation, cells were washed twice with ice-cold FACS buffer, resuspended in FACS buffer containing 100 nM SytoxGreen or 1 µM SytoxBlue (Thermo Fisher Scientific), and analyzed via flow cytometry (BD LSR II or BD Symphony).

Instrument

BD LSR II or BD Symphony

Software

Data analysis was performed using the FlowJo v.10.0 software (Tree Star)

Cell population abundance

n/a

Gating strategy

All samples are gated on live, single cells using gating strategies outlined in the Methods and Extended Data figures. Frequency of positive gates was determined using a fluorescence minus one, isotype, or other biologic control (e.g., sialidase treatment) as noted in the Methods.

☒ Tick this box to confirm that a figure exemplifying the gating strategy is provided in the Supplementary Information.
